# The Standard Model from LHC to future colliders

**DOI:** 10.1140/epjc/s10052-015-3759-0

**Published:** 2015-11-25

**Authors:** S. Forte, A. Nisati, G. Passarino, R. Tenchini, C. M. Carloni Calame, M. Chiesa, M. Cobal, G. Corcella, G. Degrassi, G. Ferrera, L. Magnea, F. Maltoni, G. Montagna, P. Nason, O. Nicrosini, C. Oleari, F. Piccinini, F. Riva, A. Vicini

**Affiliations:** Dipartimento di Fisica, Università di Milano, Via Celoria 16, 20133 Milan, Italy; INFN, Sezione di Roma, Piazzale Aldo Moro 2, 00185 Rome, Italy; Dipartimento di Fisica, Università di Torino, Via P. Giuria 1, 10125 Turin, Italy; INFN, Sezione di Pisa, Largo B. Pontecorvo 3, 56127 Pisa, Italy; Dipartimento di Fisica, Università di Pavia, via Bassi 6, 27100 Pavia, Italy; INFN, Sezione di Pavia, via Bassi 6, 27100 Pavia, Italy; Dipartimento di Chimica, Fisica e Ambiente, Università di Udine, Via delle Scienze, 206, 33100 Udine, Italy; INFN, Laboratori Nazionali di Frascati, Via E. Fermi 40, 00044 Frascati, Italy; Dipartimento di Matematica e Fisica, Università’ Roma Tre, Via della Vasca Navale 84, 00146 Rome, Italy; Centre for Cosmology, Particle Physics and Phenomenology (CP3), Université Catholique de Louvain, 1348 Louvain-la-Neuve, Belgium; Dipartimento di Fisica, Università di Milano-Bicocca, Piazza della Scienza 3, 20126 Milan, Italy; Institut de Théorie des Phénoménes Physiques, École Polytechnique Fédérale de Lausanne, 1015 Lausanne, Switzerland; INFN, Sezione di Milano, Via Celoria 16, 20133 Milan, Italy; INFN, Sezione di Torino, Via P. Giuria 1, 10125 Turin, Italy; INFN, Gruppo Collegato di Udine, Via delle Scienze, 206, 33100 Udine, Italy; INFN, Sezione di Roma Tre, Via della Vasca Navale 84, 00146 Rome, Italy; INFN, Sezione di Milano-Bicocca, Piazza della Scienza 3, 20126 Milan, Italy

## Abstract

This review summarizes the results of the activities which have taken place in 2014 within the Standard Model Working Group of the “What Next” Workshop organized by INFN, Italy. We present a framework, general questions, and some indications of possible answers on the main issue for Standard Model physics in the LHC era and in view of possible future accelerators.

## Synopsis

The goal of this review is to develop the community’s long-term physics aspirations. Its narrative aims to communicate the opportunities for discovery in physics with high-energy colliders: this area includes experiments on the Higgs boson, the top quark, the electroweak and strong interactions. It also encompasses direct searches for new particles and interactions at high energy. To address these questions a debate in the community is necessary, having in mind that several topics have overlapping boundaries. This document summarizes some aspects of our current, preliminary understanding, expectations, and recommendations.

Inevitably, the starting point is the need for a better understanding of the current “We haven’t seen anything (yet)” theoretical environment. Is the Standard Model (SM) with a $125~\text {GeV}$ Higgs the Final Theory, or indeed could it be? The associated problems are known. Some of them (neutrino masses, strong CP, gauge coupling unification, cosmological constant, hierarchy problem) in principle could not be problems at all, but just a theoretical prejudice. Others (e.g., dark matter) seem rather harder to put aside. Indeed, while some of these questions point to particular energy scales and types of experiments, there is no scientific reason to justify the belief that all the big problems have solutions, let alone ones we humans can find.

Since exploration of the TeV scale is still in a preliminary stage it would be advisable to keep options open, and avoid investing all resources on a single option, be it increasing precision of theory predictions (see Ref. [1] for an extensive compilation) and experimental results, or the search for new models (and the resolution of the issue of the relevance of naturalness), see Refs. [2, 3].

In order to set up a framework for addressing these issues, in this introduction we draw a very rough roadmap for future scenarios. The bulk of this document will then be devoted to a summary of what we believe to be the key measurements and some of the main tools which will be necessary in order to be able to interpret their results with the goal of answering some of the broader questions.

### Scenarios

It is useful to discuss possible scenarios separating three different timescales: a short one, which more or less coincides with the lifetime of LHC and its luminosity upgrade (HL-LHC), a medium one, related to an immediate successor of the LHC (such as the ILC), and a long timescale for future machines such as future circular colliders (such as a FCC).

#### Scenarios for LHC physics

In the short run, a possible scenario is that nothing but the Standard Model is seen at LHC energies, with no detection of dark matter: an uncomfortable situation, in view of the fact that dark matter is at least ten percent of the mass density of the Universe [4]. A minimal approach to this situation could be to simply ignore some of the problems (hierarchy, gauge coupling unification, strong CP, cosmological constant), and extend the SM in the minimal way that accommodates cosmological data. For instance, introduce real scalar dark matter, two right-handed neutrinos, and a real scalar inflaton. With the risk, however, of ending up with a Ptolemaic theory, in which new pieces of data require ever new epicycles.

A more agreeable scenario (obviously, a subjective point of view) is one in which nonstandard physics is detected in the Higgs sector (or, possibly less likely, in the flavor sector). This could possibly occur while looking at the Higgs width (possibly through interferometry  [5–10] beyond the narrow width approximation [6]), decays (including vector meson [11] and rare Dalitz [12]), and more generally anything that would use the Higgs as a probe for new physics (Higgs, top-Higgs anomalous production modes, with new loop contributions, associate productions, trilinear couplings).

It is likely that, if such a discovery (i.e., a discovery connected to Higgs interactions) will happen, it will be through the combination of electroweak precision data with Higgs physics [13]. This means, on the one hand, controlling the general features of electroweak symmetry breaking (EWSB), specifically through the determination of SM parameters (${M_{\mathrm{t}}}, {M_\mathrm{W}}, \alpha _{\mathrm{s}}$, etc.) from global fits but also through the study of processes which are directly sensitive to EWSB, such as ${\mathrm{V}}{}{}{\mathrm{V}}{}{}\,$-scattering [14]. On the other hand, it means developing predictions and tools [15, 16] to constrain the space of couplings of the effective field theory (EFT).

The most powerful strategy for looking for deviations from the SM [17] will require the determination of the Wilson coefficients for the most general set of $\mathrm {dim}=6$ operators (see Ref. [18]). With enough statistics these could be determined, and they will then be found to be either close to zero (their SM value), or not. In the former case, we would conclude that both next-to-leading (NLO) corrections (and the residual theoretical uncertainty at NNLO level) and the coefficients are small: so the SM is actually a minimum in our Lagrangian space or very close to it. This would be disappointing, but internally consistent, at least up to the Planck scale [2].

The latter case would be more interesting, but also problematic. Indeed, operators whose coefficients are found to be large would have to be treated beyond leading-order (LO). This means that we should move in the Lagrangian space and adopt a new renormalizable Lagrangian in which the Wilson coefficients become small; local operators would then be redefined with respect to the new Lagrangian. Of course there will be more Lagrangians projecting into the same set of operators but still we could see how our new choice handles the rest of the data. In principle, there will be a blurred arrow in our space of Lagrangians, and we should simply focus the arrow. This is the so-called inverse problem [19]: if LHC finds evidence for physics beyond the SM, how can one determine the underlying theory? It is worth noting that we will always have problems at the interpretation level of the results.

The main goal in the near future will be to identify the structure of the effective Lagrangian and to derive qualitative information on new physics; the question of the ultraviolet completion [20, 21] cannot be answered, or at least not in a unique way, unless there is sensitivity to operators with dimension greater than 6. The current goals are therefore rather less ambitious that the ultimate goa;l of understanding if the effective theory can be UV completed [22–25].

What might actually be needed is an overall roadmap to Higgs precision measurements. From the experiments we have some projections on which experimental precision is reachable in different channels in the next few years. The logical next step would be to determine what kind of theoretical precision we need for each channel to match this experimental precision, as a function of time. Based on this, we can then define the precision we need for each parameter measurements using EFT. This then determines in a very general way what kind of work is needed from the theory community.

#### Physics at the ILC

As a next step, ILC [26] plans to provide the next significant step in the precision study of Higgs boson properties [27]. LHC precision measurements in the 5–10 % range should be brought down to the level of $1\,\%$. But this means that the strategy discussed above [17] must be upgraded by the inclusion of higher order electroweak corrections.

This is not precision for precision’s sake, rather, the realization that precision measurements and accurate theory predictions represent a fundamental approach in the search for new physics beyond the SM. For instance, while a machine with limited precision may only claim a discovery of a SM-like Higgs boson, once greater precision is achieved, it may be possible to rule out the SM nature of the Higgs boson through the accurate determination of its couplings. A tantalizing example of such a situation is provided by the current status of the vacuum stability problem: the vacuum is at the verge or being stable or metastable, and a sub-percent change of $$\sim $$$$ 1~\text {GeV}$$ in either $${M_{\mathrm{t}}}$$ or $${M_{\mathrm{H}}}$$ is all it takes to tip the scales [28, 29].

This, however, raises new challenges. For example, the ILC plans to measure $\sigma _{{\mathrm{Z}}{}{}{\mathrm{H}}{}{}}$. Of course, this is a pseudo-observable: there are neither ${\mathrm{Z}}{}{}$ nor ${\mathrm{H}}{}{}$ particles in a detector. Precision physics thus raises the issue of how “unobservable” theoretical entities are defined, which is a very practical issue, given that an unobservable quantity is not uniquely defined (what is the up quark mass? or even the top quark mass?).

It is important however to understand that naturalness, which has been perhaps so far the main guiding principle, has largely lost this role [2, 30]. It is still well possible that naturalness can be relaxed to a sufficient extent that it still holds in some plausible sense – after all, the SM is a renormalizable theory, up to Landau poles it is completely fine and predictive, and it can thus stretched at will [31]. It is plausible to assume that Nature has a way, still hidden to us, to realize a deeper form of naturalness at a more fundamental level, but this gives no guidance on the relevant scale: we then have no alternative to looking for the smallest possible deviations.

#### The far future

Given that sufficiently precise measurements of the Higgs properties and the EWSB parameters are ideal probes for the new physics scale, a future circular collider (FCC) could be the best complementary machine to LHC. This includes the, partly complementary, ${\mathrm{e}}{}{}{\mathrm{e}}{}{}$, ${\mathrm{e}}{}{}{\mathrm{p}}{}{}$ and ${\mathrm{p}}{}{} {\mathrm{p}}{}{}$ options. At $\sqrt{s} = 500~\text {GeV}$ the luminosity of a FCC-*ee* [32] and ILC would be comparable; additional luminosity would improve the precision of Higgs couplings of only a factor $\sqrt{2}$. However, the opening of ${{\mathrm{e}}{}{}}^{+} {{\mathrm{e}}{}{}}^{-} \rightarrow \bar{{\mathrm{t}}{}{}}{}{} {\mathrm{t}}{}{} {\mathrm{H}}{}{}$ process allows the $\bar{{\mathrm{t}}{}{}}{}{} {\mathrm{t}}{}{} {\mathrm{H}}{}{}$ coupling to be measured, with a global fit, with a precision of $10\,\%$ at the FCC-*ee*. The potential of the ${\mathrm{e}}{}{}{\mathrm{p}}{}{}$ and ${\mathrm{p}}{}{} {\mathrm{p}}{}{}$ options has just started being explored.

#### A new frontier?

As we already mentioned several times, many of the currently outstanding problems – naturalness, the UV behavior of the Higgs sector – point to the possibility that electroweak symmetry breaking may be linked to the vacuum stability problem: is the Higgs potential at $M_{\mathrm {plank}}$ flat [28, 29], and if so, why? This then raises the question whether perhaps EWSB might be determined by Planck-scale physics, which, in turn, begs the question of the matching of the SM to gravity. Of course, BSM physics (needed for dark matter) could change the picture, by making the Higgs instability problem worse, or by curing it.

But the fact remains that we do not have a renormalizable quantum field theory of gravity. The ultimate theoretical frontier then would be understanding how to move beyond quantum field theory.

### Measurements and tools

In order to address the issues outlined in the previous section, a number of crucial measurements are necessary. Some of these have a clear time frame: for example, Higgs couplings will surely be extensively measured at the LHC, while double Higgs production (and trilinear Higgs couplings) will only be accurately measured at future accelerators. Other measurements will be performed with increasing precision on different timescales. Extracting from these measurements the information that we are after will in turn require the development of a set of analysis tools.

The main purpose of this note is to summarize the status and prospects for what we believe to be some of the most important directions of progress, both in terms of measurement and tools.

First, we will discuss crucial measurements. Specifically, we will address gauge boson mass measurement, that provide perhaps the most precise of SM standard candles. We will then discuss the mass of the top quark, which, being the heaviest fundamental field of the Standard Model Lagrangian provides a natural door to new physics. We will finally address the Higgs sector: on the one hand, by analyzing our current and expected future knowledge of the effective Lagrangian for the Higgs sector (up to dimension six operators), and then by discussing the implications for the stability of the electroweak vacuum, which is ultimately related to the way the Standard Model may open up to new physics.

We will then turn to some selected methods and tools: resummation techniques, which are expected to considerably improve the accuracy and widen the domain of applicability of perturbative QCD computations, and then the Monte Carlo tools which provide the backbone of data analysis, both for the strong and the electroweak interactions.

**Fig. 1:**
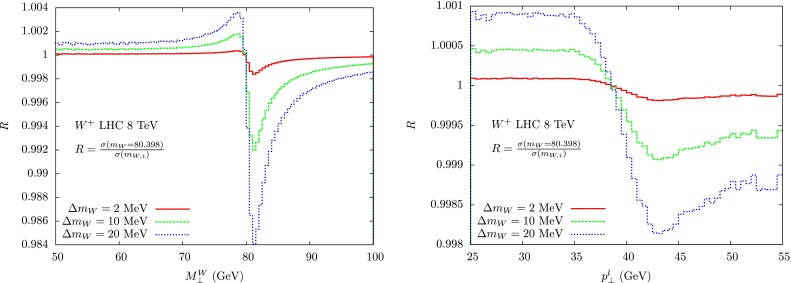
Ratio of lepton-pair transverse mass (*left*) and lepton transverse momentum (*right*) distributions which have been generated with different ${\mathrm{W}}{}{}$ boson masses

## The ${\mathrm{W}}{}{}$ and ${\mathrm{Z}}{}{}$ mass and electroweak precision physics

### Introduction

#### Relevance of a high-precision $m_{\scriptscriptstyle W}$ measurement

The ${\mathrm{W}}{}{}$ boson mass has been very precisely measured at the Tevatron CDF ($m_{\scriptscriptstyle W}=80.387\pm 0.019~\text {GeV}$) [33] and D0 ($m_{\scriptscriptstyle W}=80.375\pm 0.023~\text {GeV}$) [34] experiments, with a world average now equal to $m_{\scriptscriptstyle W}=80.385\pm 0.015~\text {GeV}$ [35]. There are prospects of a further reduction of the total error by the LHC experiments, and a value of 15 or even $10~\text {MeV}$ is presently discussed [36, 37]. These results offer the possibility of a high precision test of the gauge sector of the Standard Model (SM). The current best prediction in the SM is ($m_{\scriptscriptstyle W}=80.361\pm 0.010~\text {GeV}$) from muon decay [38]; it has been computed including the full 2-loop electroweak (EW) corrections to the muon decay amplitude [39] and partial three-loop [${\mathcal O}(\alpha \alpha _{s}^{2})$, ${\mathcal O}(\alpha _{t}^{2}\alpha _{s})$, ${\mathcal O}(\alpha _{t}^{3})$] and four-loop QCD corrections ${\mathcal O}(\alpha _{t}\alpha _{s}^{3})$, where $\alpha _{t}=\alpha m_{t}^{2}$ [40–42]. Alternatively, the value $m_{\scriptscriptstyle W}=80.358\pm 0.008~\text {GeV}$ is obtained from a global EW fit of the SM [13]. The error on this evaluation is mostly due to parametric uncertainties of the inputs of the calculation, the top mass value, the hadronic contribution to the running of the electromagnetic coupling, and also to missing higher-order corrections.

The comparison of an accurate experimental value with the predictions of different models might provide an indirect signal of physics beyond the SM [43]. The value $m_{\scriptscriptstyle W}^i$ computed in model *i* follows form the relation $\frac{G_\mu }{\sqrt{2}} = \frac{g^2}{8(m_{\scriptscriptstyle W}^i)^2} (1+\Delta r^i)$ where the radiative corrections to the muon decay amplitude are represented by the parameter $\Delta r^i=\Delta r^i(m_{\scriptscriptstyle W}^i,M_{\mathrm {SM}},M_{\mathrm {BSM}})$ and possibly offer sensitivity to new particles present in the considered extension *i* of the SM, whose mass scale is generically indicated with $M_{\mathrm {BSM}}$.

#### Physical observables

At hadron colliders, the ${\mathrm{W}}{}{}$ boson mass is extracted from the study of the charged-current (CC) Drell–Yan (DY) process,  (and also ). In the leptonic final state the neutrino is not measured, so that the invariant mass of the lepton pair can not be reconstructed. The value of $m_{\scriptscriptstyle W}$ is determined from the study of the lepton transverse momentum, of the missing transverse energy and of the lepton-pair transverse mass distributions. These observables have an enhanced sensitivity to $m_{\scriptscriptstyle W}$ because of their jacobian peak at the ${\mathrm{W}}{}{}$ resonance [44]. More precisely, it is the study of their shape, rather than the study of their absolute value, which provides informations about $m_{\scriptscriptstyle W}$. These observables are defined in terms of the components of the lepton momenta in the transverse plane. The main experimental uncertainties are related to the determination of the charged lepton energy or momentum on one side, and, on the other side, to the reconstruction of the missing transverse energy distribution, so that the neutrino transverse momentum can be inferred in an accurate way. The modeling of the lepton-pair transverse momentum distribution also plays a major role in the determination of the neutrino components. A systematic description of the size of the experimental uncertainties affecting the measurement and of their impact on the $m_{\scriptscriptstyle W}$ measurement can be found in [33, 34, 36, 37].

#### Sensitivity to $m_{\scriptscriptstyle W}$ of different observables

The sensitivity of the observables to the precise $m_{\scriptscriptstyle W}$ value can be assessed with a numerical study of their variation under a given shift of this input parameter. In Fig. 1 we show the ratio of two distributions obtained with $m_{{\scriptscriptstyle W}0}=80.398~\text {GeV}$ and shifted, $m_{{\scriptscriptstyle W},i }=m_{{\scriptscriptstyle W}0}+\Delta m_{\scriptscriptstyle W}$. The distortion of the shapes amounts to one to few parts per mill, depending if one considers the lepton transverse momentum or the lepton-pair transverse mass. We can rephrase this remark by saying that a measurement of $m_{\scriptscriptstyle W}$ at the $10~\text {MeV}$ level requires the control of the shape of the relevant distributions at the per mill level. The codes used to derive the results in Fig. 1 do not include the detector simulation; the conclusions about the sensitivity to $m_{\scriptscriptstyle W}$ should be considered as an upper limit, which can be reduced by additional experimental smearing effects.

The ${\mathrm{W}}{}{}$ boson mass is measured by means of a template fit approach: the distributions are computed with Montecarlo simulation codes for different values of $m_{\scriptscriptstyle W}$ and are compared with the corresponding data; the value which maximizes the agreement is chosen as the preferred value. The templates are theoretical objects, computed with some assumptions about input parameters, proton PDF choices and perturbative accuracy. The uncertainties affecting the templates, missing higher orders, PDF and input parameters uncertainties, have an impact on the result of the fit and should be treated as a theoretical systematic error.

### Available tools and sources of uncertainty

The DY reaction in LO is a purely EW process, which receives perturbative corrections due to the EW and to the QCD interactions; in higher orders also mixed QCD-EW contributions appear and are of phenomenological relevance. The observables under study have a different behaviour with respect to the perturbative corrections, so that in some cases a fixed-order prediction is not sufficient and the resummation to all orders of logarithmically-enhanced contributions becomes necessary. With the resummation, three different kinds of entangled ambiguities appear in the preparation of the templates: (1) missing higher-order logarithmically-enhanced terms in the resummed expression, (2) ambiguities of the matching between fixed-order and all-order results, (3) the interplay, in the region of low lepton-pair transverse momenta, of perturbative and non-perturbative QCD corrections. This latter source of uncertainty is also related to the non-perturbative effects parametrized, in the collinear limit, in the proton PDFs.

The $m_{\scriptscriptstyle W}$ value follows from the precise study of the shape of the observables; for this reason, the use of distributions normalized to their respective integrated cross sections removes an important class of uncertainties associated to the DY total rate determination.

#### EW radiative corrections

EW radiative corrections to CC and neutral-current (NC) DY are available with NLO-EW accuracy and are implemented in several public codes: WZGRAD [45, 46], RADY [47], SANC [48], HORACE [49, 50]. The effect of multiple photon emissions is accounted for in HORACE by a QED Parton Shower (PS), properly matched with the fixed-order calculation; higher-order universal effects, that can be reabsorbed in a redefinition of the tree-level couplings, are also available in the above codes and play an important role in the description of the NC invariant mass distribution.

Real-photon emissions from the final state leptons greatly modify the value of the measured lepton energies and momenta. The distortion of the jacobian peak is at the level of 5–18 %, depending on the observable, on the kind of lepton and on the procedure that recombines QED radiation that surrounds the lepton into an effective calorimetric object. The impact at ${\mathcal O}(\alpha )$ of this radiation can be estimated to yield a shift of $m_{\scriptscriptstyle W}$ of ${\mathcal O}(150 \,~\text {MeV})$ [33]. Additional radiation induces a further change in the result of ${\mathcal O}(10\,\%)$ of the ${\mathcal O}(\alpha )$ effect.

Subleading terms, i.e. not enhanced by a final state lepton mass logarithm, are exactly available as part of the ${\mathcal O}(\alpha )$ calculation and are partially available at ${\mathcal O}(\alpha ^2)$ thanks to the matching procedure between QED PS and exact ${\mathcal O}(\alpha )$ matrix elements. Their impact amounts to a few contributions, each yielding a shift of ${\mathcal O}(5 \,~\text {MeV})$. The residual uncertainty due to missing higher orders has been estimated to be smaller than $5~\text {MeV}$, in the framework of a purely EW analysis; it should be however kept in mind that the interplay of EW and QCD corrections leads, for some observables like e.g. the lepton tranvserse momentum distribution, to an increase of the purely EW estimate.

#### QCD radiative corrections

QCD corrections to lepton-pair production are available at fully differential level through ${\mathcal O}(\alpha _{\mathrm{s}}^2)$ and are implemented in the Montecarlo integrators FEWZ [51], DYNNLO [52] and SHERPA [53]. The gauge boson transverse momentum distribution is known with NNLL+NLO accuracy (and with NNLO accuracy on the total cross section) and is implemented in the Montecarlo integrator DYqT [54], without the description of the decay into leptons.^1^ The NNLL resummation, without the NNLO accuracy on the total cross section, is available in the integrator ResBos [55, 56]. The effects on the total cross section and on the gauge boson rapidity distribution of the logarithmic threshold corrections have been included up to N$^3$LO+NNLL accuracy [57, 58]. Standard tools for the experimental analyses are the Shower Montecarlo (SMC) event generators with NLO-QCD accuracy, like MC@NLO [59] or POWHEG [60] (more recently HERWIG [61] or SHERPA [62]). They have NLO-QCD accuracy on the total cross section, but only LO-QCD accuracy in the description of the lepton-pair transverse momentum. Recently, progresses have been made in the direction of a merging of NNLO-QCD matrix elements with a QCD PS, in SHERPA [53] or in NNLOPS [63] or in GENEVA [64].

The QCD corrections have important effects on the DY observables in terms of absolute normalization and in terms of shapes. The former can be mitigated by considering normalized distributions, while the latter are the most critical ingredient in the theoretical framework. Among the observables relevant for the $m_{\scriptscriptstyle W}$ measurement, the lepton transverse momentum distribution is a paradigmatic example: its prediction in fixed order is affected by the very large logarithmic corrections for small lepton-pair transverse momenta and only after their resummation a sensible description becomes possible. In this case, the evaluation of the QCD uncertainty on $m_{\scriptscriptstyle W}$ is possible with a joint systematic study of matching ambiguities, renormalization/factorization scale variations, of the effect of subleading logarithmic terms and of the modeling of the non-perturbative effects at very low transverse momenta [54, 65]. A very naive estimate of the combination of all these effects, in a simplified setup, might be translated into a shift of the measured $m_{\scriptscriptstyle W}$ by ${\mathcal O}(50{-}100)~\text {MeV}$, which would clearly be a dramatic conclusion of the uncertainty analysis. It has been proposed in [66] to consider ratios of ${\mathrm{W}}{}{}$ and ${\mathrm{Z}}{}{}$ observables, with an evident reduction of the scale uncertainties both in size and in shape. A study of the residual uncertainty on $m_{\scriptscriptstyle W}$ in this approach is in progress [67]. The published Tevatron results [33, 34] do not quote a comprehensive QCD uncertainty that includes perturbative effects; they rather use the generator ResBos with a fixed choice of the perturbative scales and of the proton PDF to describe the ${\mathrm{Z}}{}{}$ boson transverse momentum distribution; this analysis allows to fit the parameters of a model describing the non-perturbative low-transverse-momentum components of QCD radiation, which are then used to simulate the CC DY process; this approach assumes universality of these parameters and their independence on the process energy scale. In the Tevatron analyses the error assigned to the $p_\perp ^{{\mathrm{W}}{}{}}$ modeling is only due to a variation of the non-perturbative parameters in the range allowed by the fit of the ${\mathrm{Z}}{}{}$ boson data.

The impact of the different QCD uncertainties mentioned above is milder in the case of the lepton-pair transverse mass, because this observable is more stable with respect to the inclusion of higher-order QCD corrections. The shape distorsion observed when comparing its NLO- and NNLO-QCD determinations is minimal; the scale variations do not significantly modify the shape around the jacobian peak, and so the impact on the $m_{\scriptscriptstyle W}$ determination is limited.

#### Proton PDF uncertainty

The proton PDFs enter in the $m_{\scriptscriptstyle W}$ determination because they are needed to compute the templates used in the fit of the data. Different PDF set choices, or different replica choices within the same set, imply a change of the templates shape and in turn of the preferred $m_{\scriptscriptstyle W}$ value. The propagation of the PDF error is computed according to the prescription of each PDF collaboration, and eventually the different results can be combined with the PDF4LHC prescription [68, 69].

Neglecting all detector effects, which have an important impact on the acceptance determination, the PDF uncertainty on the $m_{\scriptscriptstyle W}$ extracted from the study of the normalized lepton-pair transverse mass distribution remains below the $10~\text {MeV}$ level [70, 71], whereas the spread in the case of the lepton transverse momentum distribution, again estimated at generator level, ranges between 6 and $18~\text {MeV}$, depending on the chosen PDF set, collider energy and final state [72]. A crucial role is played by the acceptance cuts, on the leptons but also an the lepton pair. At higher collider energies, the PDF uncertainty associated to the lepton-pair transverse mass remains stable, whereas the one on $m_{\scriptscriptstyle W}$ extracted from the lepton transverse momentum distribution increases for proton-proton collider energies between 8 and $100~\text {TeV}$ (cfr. Table 1); the application of a cut $p_\perp ^{{\mathrm{W}}{}{}}<15~\text {GeV}$ on the lepton-pair transverse momentum keeps the estimated uncertainty below the 1$5~\text {MeV}$ level [72].

**Table 1:** Estimate of the central values and of the PDF uncertainty on $m_{\scriptscriptstyle W}$, extracted from the normalized lepton transverse momentum distributions simulated with the NNPDF3.0_nlo_as_0118 PDF set and with the POWHEG NLO-QCD event generator matched with the PYTHIA 6.4.26 QCD Parton Shower. The fit interval is $p_\perp ^l \in [29,49]~\text {GeV}$. The templates used in the fit have been prepared with NNPDF2.3_nlo_as_0118

	Normalized distribution, additional cut $p_\perp ^{{\mathrm{W}}{}{}}<15~\text {GeV}$			
	8 $~\text {TeV}$	13 $~\text {TeV}$	33 $~\text {TeV}$	100 $~\text {TeV}$
$W^+$	$80.395 \pm 0.009$	$80.400 \pm 0.010$	$80.402 \pm 0.010$	$80.404 \pm 0.013$
$W^-$	$80.398 \pm 0.007$	$80.391 \pm 0.006$	$80.385 \pm 0.007$	$80.398 \pm 0.011$

#### Mixed QCD-EW radiative corrections

QCD corrections, via initial state radiation, modify the kinematics of the DY events, whereas the leading EW effects are due to a variation of the lepton spectra due to final state radiation. The interplay between these two groups of corrections is not trivial and strongly depends on the observable under study. The first perturbative order at which these mixed corrections appear is ${\mathcal O}(\alpha \alpha _{\mathrm{s}})$, but no exact calculation is available, so that one has to rely on some approximations. The NLO-QCD and NLO-EW exact matrix elements have been implemented in POWHEG and have been consistently matched with both QCD-PS and QED-PS for CC [73, 74] and NC [75] DY. In this approach all the QCD-LL (initial state collinear logarithms) and all the QED-LL (final state mass logarithms) corrections, in all possible combinations, are taken into account, including the leading ${\mathcal O}(\alpha \alpha _{\mathrm{s}})$ terms. The first terms that are beyond the accuracy of the code are of ${\mathcal O}(\alpha \alpha _{\mathrm{s}})$ and subleading in the expansion with respect to the EW logarithms. The role of the mixed corrections is particularly relevant in the prediction of the lepton transverse momentum distribution [74, 75]. For this quantity, as discussed in [76, 77], a naive factorization recipe to combine QCD and EW corrections, fails. The POWHEG implementation of the QCD-EW combination misses, on one hand, subleading effects of ${\mathcal O}(\alpha \alpha _{\mathrm{s}})$; it provides, on the other hand, an exact treatment of the kinematics of each radiated parton and thus gives the correct convolution of QCD and EW corrections including those effect that break the factorization *ansatz*. The study of the impact of different combinations of QCD and EW effects, with and without NLO accuracy, is in progress [78].

### Prospects of improvement

Let us briefly discuss the prospects for a high-precision measurement of $m_{\scriptscriptstyle W}$ at a high- energy/luminosity proton-proton collider in the next 10–20 years, under the assumption that progresses that today can be wished, or expected in the long term, will be available.

#### Montecarlo generators

1. Definition of a matching procedure that allows a Montecarlo event generator to reach NNLO-QCD accuracy on the DY total cross section and NNLL-QCD accuracy in the resummation of the logarithms of the lepton-pair transverse momentum (partial results are already available, by different groups).
2. Evaluation of the N$^3$LO-QCD corrections to the DY processes, as the first step towards the construction of an integrator code that reaches N$^3$LO accuracy on the total cross section and N$^3$LL accuracy in the resummation of the logarithms of the lepton-pair transverse momentum (the results in the soft limit are already available, by different groups). The formulation of an integrator with this accuracy on the lepton-pair transverse momentum is intertwined with the consistent definition of the non-perturbative contributions to the same observable. With a similar tool, and with the event generator of item (1), the evaluation of the ratio of ${\mathrm{W}}{}{}$–${\mathrm{Z}}{}{}$ observables should be sufficiently stable from the QCD point of view and the residual corresponding uncertainty on $m_{\scriptscriptstyle W}$ could fall down to the $5~\text {MeV}$ level; this estimate is, at the moment, a guess that can become more sound after the estimate with the presently available tools of the QCD uncertainty on $m_{\scriptscriptstyle W}$ extracted from ratios of ${\mathrm{W}}{}{}$ over ${\mathrm{Z}}{}{}$ observables.
3. Completion of the full calculation of the corrections at ${\mathcal O}(\alpha \alpha _{\mathrm{s}})$ to the DY processes, to fix the ambiguity affecting the combination of QCD-EW corrections at the first non trivial order (partial results in the ${\mathrm{W}}{}{}$ pole approximation are already available, matrix elements for different subprocesses that contribute at this order are available). The analysis of the purely EW effects on the $m_{\scriptscriptstyle W}$ determination indicates a residual uncertainty at the $5~\text {MeV}$ level, but suffers from being a LO-QCD study; the inclusion of the ${\mathcal O}(\alpha \alpha _{\mathrm{s}})$ corrections will make the conclusion more stable against QCD-scale variations.
4. Determination of proton PDFs which can be consistently matched with an ${\mathcal O}(\alpha \alpha _{\mathrm{s}})$ calculation (NLO accuracy mixed QCD-EW).
5. Completion of the calculation of the full set of ${\mathcal O}(\alpha ^2)$ corrections, to reduce the uncertainties in the calibration phase (${\mathrm{Z}}{}{}$ mass determination and precise understanding of the absolute lepton energy scale).

#### Uncertainty reduction with higher energy/luminosity

We compare the perspective at future colliders for a measurement of $m_{\scriptscriptstyle W}$ from the lepton transverse momentum and from the lepton-pair transverse mass. With the high luminosity projected at a high-energy (13, 33 or 100 TeV) hadron collider, and in particular with the high-luminosity programs planned at 13 TeV, the number of events useful for an accurate $m_{\scriptscriptstyle W}$ measurement will be extremely large, making the uncertainty of statistical nature negligible, compared to those of systematic origin (theoretical and experimental).

*Higher energy and PDFs*. The energy scale of the DY processes, relevant for the ${\mathrm{W}}{}{}$ mass measurement, is given by the masses of the ${\mathrm{W}}{}{}$ and ${\mathrm{Z}}{}{}$ gauge bosons. An increase of the center-of-mass energy of a hadron collider reduces the values of the partonic-*x*, the fraction of the hadron momenta carried by the colliding partons, relevant to produce a final state of given invariant mass, and modifies the so called parton-parton luminosity, i.e. the effective number of colliding partons, and eventually the cross section. The change of collider energies has thus an impact on the PDF uncertainty, because of the different partonic-*x* range probed. The PDF uncertainty on $m_{\scriptscriptstyle W}$ measured from the lepton-pair transverse mass distribution is already today at the $10~\text {MeV}$ level and is improving as long as LHC data become available, with some realistic chances that a contribution to the uncertainty on $m_{\scriptscriptstyle W}$ will become soon of the order of $5~\text {MeV}$ [43, 71]. A preliminary estimate, at generator level, of the PDF uncertainty associated to the lepton transverse momentum distribution, using only the PDF set NNPDF3.0, can be found in Table 1. These results assume the possibility of a cut on the lepton-pair transverse momentum; in the case that such an assumption could not be verified, a steeper growth of the uncertainty, up to ${\mathcal O}(25)~\text {MeV}$ at 100 TeV, would be observed.

It will require a global effort to reduce the present ${\mathcal O}(20)~\text {MeV}$ uncertainty down below the $10~\text {MeV}$ level, because of the contribution to the uncertainty of all the parton densities in a wide range of partonic *x*. The use of ratios of ${\mathrm{W}}{}{}$ over ${\mathrm{Z}}{}{}$ observables should partially reduce the PDF uncertainty, especially the one associated to gluon-induced subprocesses.

*Higher luminosity and neutrino momentum determination.* The very large number of collisions occuring at each bunch crossing in the collider will make the so-called pile-up phenomenon more and more pronounced with higher collider luminosity: the latter increases the hadronic activity in the transverse plane, making the reconstruction of the missing transverse momentum (and eventually of the neutrino transverse momentum) problematic. As a consequence, the uncertainty on the shape of the lepton-pair transverse mass will limit the possibility of a high-precision measurement.

### Conclusions

- The progress in the calculation of higher order QCD and EW corrections seems to offer some chances that adequate theoretical tools will become available to perform a $m_{\scriptscriptstyle W}$ measurement at the $10~\text {MeV}$ level.
- The lepton transverse momentum distribution has a very clean experimental definition and does not suffer from the pile-up problems that show-up with high-luminosity conditions, provided that appropriate lepton isolation criteria are validated and applied. On the other hand it is extremely sensitive to any detail of the QCD description, both in the perturbative regime and for what concerns the PDF uncertainties, which could jeopardise any hope of measuring $m_{\scriptscriptstyle W}$ at the $10~\text {MeV}$ level. The definition of ${\mathrm{W}}{}{}$ over ${\mathrm{Z}}{}{}$ ratios could be the clue to significantly reduce all common theoretical systematics, as demonstrated in [66]; this same approach could also help to mitigate the PDF uncertainty. The availability of predictions with N$^3$LO+N$^3$LL accuracy should make it possible to reduce the QCD systematic error below the $10~\text {MeV}$ level.
- The lepton-pair transverse mass distribution has a very mild dependence on the details of QCD corrections, so that it should be possible to make its theoretical prediction accurate enough, to contribute with a systematic error at the $10~\text {MeV}$ level. The PDF uncertainty on this observable is moderate and will benefit of the inclusion of more LHC data in the global PDF fit. On the other hand, the accuracy of the measurement will deteriorate in presence of higher luminosity conditions, mostly because of increasing pile-up effects that disturb the identification of the hard scattering process.

## Top quark physics

### Introduction

The top quark, discovered in 1995 [79, 80], is nowadays the heaviest among the known elementary particles. It plays a crucial role in the Standard Model phenomenology and the electroweak symmetry breaking: thanks to its large mass, it exhibits the largest Yukawa coupling with the Higgs boson and therefore it is very important in the precision tests of the electroweak interactions. The top-quark mass $m_{{\mathrm{t}}{}{}}$ is a fundamental parameter of the Standard Model: even before the Higgs boson discovery [81, 82], it was used, together with the ${\mathrm{W}}{}{}$ boson mass, to constrain the Higgs boson mass in the global fits. With few exceptions which will be discussed in the following, all measurements for both ${\mathrm{t}}{}{} \bar{{\mathrm{t}}{}{}}{}{}$ and single-top production are in agreement with the Standard Model expectations. Nevertheless, top quark phenomenology will remain one of the main fields of investigation in both theoretical and experimental particle physics, at any present and future facility, i.e., both lepton and hadron colliders, as well as linear and circular accelerators. Hereafter, we shall discuss the future perspectives regarding the measurement of the top-quark properties, taking particular care about its mass, couplings and final-state kinematic distributions.

### Top quark mass

The mass of the top quark ($m_{{\mathrm{t}}{}{}}$) is a fundamental physical quantity and its current world average is $m_{{\mathrm{t}}{}{}}=173.34\pm 0.27$ (stat) $\pm$ 0.71 (syst)  GeV[83]. Besides its role in the precision tests, it was found that, using updated values for Higgs and top masses and assuming that possible new physics interactions at the Planck scale do not affect the stability phase diagram and the electroweak vacuum lifetime (see, e.g., [84] for an alternative treatment of this point), the Standard Model vacuum lies on the border between stability and metastability regions [28]. This result implies that, if the central value of $m_{{\mathrm{t}}{}{}}$ had to shift or the uncertainty got reduced or enhanced, the vacuum may still sit on the border between stability and metastability zones, or be located completely inside one of them. Therefore, it is mandatory to measure the top mass with the highest possible precision and having all sources of errors under control. Moreover, a crucial assumption employed by the authors of [28] is that the measured mass corresponds to the top-quark pole mass. Nevertheless, as will be clarified later on, the connection between the top mass measured in current analyses of experimental data and the pole mass is not straightforward and, although the two values should be reasonably close, any effort to clarify the top mass interpretation is important in order to validate or modify the outcome of electroweak fits or the study in Ref. [28].

Furthermore, the top mass plays a role in inflationary universe theories and in the open issue regarding whether the inflaton can be the Higgs field or not. As discussed, e.g., in [85], in inflationary theories the running of the couplings is important and, once the Yukawa coupling is determined from the top mass, the spectral index crucially depends on both the top and Higgs masses.

The standard methods to measure the top mass at hadron colliders, where ${\mathrm{t}}{}{} \bar{{\mathrm{t}}{}{}}{}{}$ pairs are produced in ${\mathrm{q}}{}{} \bar{{\mathrm{q}}{}{}}{}{}$ (dominant at the Tevatron) or ${\mathrm{g}}{}{} {\mathrm{g}}{}{}$ (dominant at the LHC) annihilation, are based on the investigation of the properties of the final states in top decays (${\mathrm{t}}{}{} \rightarrow {\mathrm{b}}{}{} {\mathrm{W}}{}{}$), which, according to the ${\mathrm{W}}{}{}$ decay mode, are classified as all leptons, leptons+jets or all jets. In all cases, there are two b-tagged jets, whereas the ${\mathrm{W}}{}{}$ decay products are reconstructed as isolated leptons (muons or electrons) or as jets (for ${\mathrm{W}}{}{}\rightarrow {\mathrm{q}}{}{} \bar{{\mathrm{q}}{}{}}{}{} '$ processes). After requiring energy-momentum conservation and constraining the ${\mathrm{W}}{}{}$ mass, the final-state invariant-mass distribution exhibits a peak, which is interpreted as the production of a top quark.

The conventional likelihood-type techniques to reconstruct the top mass are the matrix-element and template methods. The matrix-element method compares the measured quantities with predictions obtained by convoluting the LO ${\mathrm{t}}{}{} \bar{{\mathrm{t}}{}{}}{}{}$ cross section with the detector response. The template method is based on investigating several distributions of observables depending on $m_{{\mathrm{t}}{}{}}$, under the assumption that the final state is ${\mathrm{W}}{}{}{\mathrm{b}}{}{} {\mathrm{W}}{}{}{\mathrm{b}}{}{}$ and the ${\mathrm{W}}{}{}$ mass is known; the data are then confronted with Monte Carlo templates and $m_{{\mathrm{t}}{}{}}$ is the value which minimizes the $\chi ^2$. Matrix-element and template methods are those used in the world average determination, based on the updated measurements from D0, CDF, ATLAS and CMS Collaborations. The projections for the LHC Run at $14~\text {TeV}$, with a ${\mathrm{t}}{}{} \bar{{\mathrm{t}}{}{}}{}{}$ cross section about 951 pb, according to the template/matrix-element methods [86], are quoted in Table 2.

**Table 2:** Estimated statistical and systematic uncertainties on the top mass measurement at the LHC, by using template and matrix-element methods, at 14 TeV and 100 and 300$~\mathrm{fb}^{-1}$integrated luminosity

$\mathcal{L}$ $(\mathrm{fb}^{-1})$	$\delta m_\mathrm{stat}$ (MeV)	$\delta m_\mathrm{sys}$ (MeV)
100	40	700
300	30	600

Other strategies which have been proposed are the so-called endpoint [87] and $J/\psi$ [88, 89] methods. In fact, in the dilepton channel, the endpoint of distributions like the *b*-jet+$\ell$ invariant mass $m_{b\ell }$ or the $\mu _{{\mathrm{b}}{}{} {\mathrm{b}}{}{}}$ and $\mu _{\ell \ell }$ variables, related to the ${\mathrm{b}}{}{} {\mathrm{b}}{}{}$ and $\ell \ell$ invariant masses as discussed in [90], after costraining the ${\mathrm{W}}{}{}$ and neutrino masses, are directly comparable with $m_{{\mathrm{t}}{}{}}$. Ref. [91] presents the projections for statistical and systematic errors on the top mass reconstruction by means of the endpoint method, as reported in Table 3.

**Table 3:** Projections of the expected uncertainties on the top mass, by using the endpoint method

$\sqrt{s}$ (TeV)	$\mathcal{L}$ $(\mathrm{fb}^{-1})$	$\delta m_\mathrm{stat}$ (MeV)	$\delta m_\mathrm{sys}$
13	30	400	$1~\text {GeV}$
14	300	100	$600~\text {MeV}$
14	3000	40	$500~\text {MeV}$

**Table 4:** As in Table 3, but using the $J/\psi$ method

$\sqrt{s}$ (TeV)	$\mathcal{L}$ $(\mathrm{fb}^{-1})$	$\delta m_\mathrm{stat}$	$\delta m_\mathrm{sys}$
13	30	$1~\text {GeV}$	$1.5~\text {GeV}$
14	300	$300~\text {MeV}$	$800~\text {MeV}$
14	3000	$100~\text {MeV}$	$600~\text {MeV}$

In all cases, the dominant uncertainties are the ones due to hadronization and jet energy scale.

The $$J/\psi $$ method relies instead on the fact that, although the $$B\rightarrow J/\psi $$ decay is a rare one, in the dilepton channel and exploiting the $$J/\psi \rightarrow {{\upmu }{}{} ^{+}} {{\upmu }{}{} ^{-}} $$ mode, the three-lepton invariant mass $$m_{3\ell }$$ as well as the $$m_{J/\psi \ell }$$ spectra allow a reliable fit of $$m_{{\mathrm{t}}{}{}}$$ at the LHC, especially in scenarios with both high energy and high luminosity. Table 4 contains the expectations for statistical and systematic uncertainties at $$13~\text {TeV}$$ ($${\mathcal L}=30$$$$~\mathrm{fb}^{-1}$$) and at $$14~\text {TeV}$$ ($${\mathcal L}=300$$ and 3000$$~\mathrm{fb}^{-1}$$), as presented in Ref. [91]. Given such numbers, calculating the overall uncertainty on $$m_{{\mathrm{t}}{}{}}$$ is straightforward. In all cases, the dominant source of theory error is the treatment of bottom-quark fragmentation in top decays, discussed in [92] in the framework of parton shower generators and in [93, 94] by using NLO QCD calculations. As far as possible future runs at 33 and $$100~\text {TeV}$$ are concerned, the total error on the recostruction of the top mass based on the $$J/\psi $$ method is predicted to be 1 and $$600~\text {GeV}$$, respectively [86].

Generally speaking, in most analyses the experimental results are compared with simulations based on Monte Carlo generators (an exception is the endpoint method) and, strictly speaking, the reconstructed top mass cannot be precisely identified with theoretical definitions like, e.g., the pole mass.

In fact, programs like HERWIG [95] or PYTHIA [96] are equivalent to LO QCD calculations, with the resummation of all leading (LL) and some next-to-leading soft/collinear logarithms (NLL) [97]. In order to fix a renormalization scheme and get the pole or $\overline{\mathrm{MS}}$ mass, one would need at least a complete NLO computation, while parton showers only contain the soft/collinear part of the NLO corrections. Furthermore, any observable yielded by such codes depends on parameters which are to be tuned to experimental data, in particular non-perturbative quantities, such as the shower cutoff or the parameters entering in the hadronization models, namely the cluster [98] (HERWIG) or string (PYTHIA) [99] models. In fact, in the non-perturbative phase of the event simulation, the ${\mathrm{b}}{}{}$ quark from top decay hadronizes, e.g., in a meson B$^{\pm ,0}$, by combining with a light (anti) quark $\bar{{\mathrm{q}}{}{}}{}{}$, which may come from final- as well as initial-state radiation. Since the b quark likely radiates gluons before hadronizing, the initial colour and part of the four-momentum of the top quark may well be transferred to light-flavored hadrons, rather than only B-hadrons. As a result, there is no unique way to assign the final-state particles to the initial (anti) top quark and this leads to another contribution to the uncertainty (about 300 MeV in the world average) on the top mass, when reconstructed from the invariant mass of the top-decay products.

Also, parton shower algorithms neglect the top width, $\Gamma _{{\mathrm{t}}{}{}}\simeq (2.0\pm 0.5)~\text {GeV}$, [4]) and top-production and decay phases are assumed to factorize. But $\Gamma _{{\mathrm{t}}{}{}} /m_{{\mathrm{t}}{}{}}\sim \mathcal{O}(10^{-2})$ and therefore, for a precise mass definition with an uncertainty below $1~\text {GeV}$, even width effects should be taken into account. Therefore, one often refers to the measured mass as a ‘Monte Carlo mass’, which must be related to a given theoretical definition. Since the top mass is extracted from final-state top-decay observables, relying on the on-shell kinematics of its decay products (leptons and jets), one should reasonably expect the measured mass to be close to the pole mass, which is a definition working well for an on-shell heavy quark.

In fact, calculations based on soft collinear effective theories (SCET) [100] have proved that, assuming that the Monte Carlo mass is the SCET jet mass evaluated at a scale of the order of the shower cutoff, i.e., $$Q_0\sim {\mathcal O}(1~\text {GeV})$$, it differs from the pole mass by an amount $$\sim $$$${\mathcal O}(\alpha _{\mathrm{s}}\Gamma )\sim 200~\text {MeV}$$. A foreseen investigation, which may help to shed light on this issue, is based on the simulation of fictitious top-flavoured hadrons, e.g., $$T^{\pm ,0}$$ mesons [101]. It is well known how to relate the mass of a meson to a quark mass in any renormalization scheme. Therefore, a comparison of final-state quantities with the top quark decaying before or afer hadronization, and the subsequent extraction of the top mass from their Mellin moments, can be a useful benchmark to address the nature of the reconstructed $$m_{{\mathrm{t}}{}{}}$$ and the uncertainty due to non-perturbative effects, such as colour reconnection. In standard top-quark events the top quark gets its colour from an initial-state quark or gluon and, after decaying, gives it to the bottom quark; on the contrary, if it forms $${\mathrm{t}}{}{} $$-hadrons, it is forced to create a colour-singlet.

More recently, in order to weaken the dependence on the shower algorithms and non-perturbative corrections, other methods have been proposed to measure the top mass at the LHC. One can use the total ${\mathrm{t}}{}{} \bar{{\mathrm{t}}{}{}}{}{}$ cross section, recently computed to NNLO+NNLL accuracy [102], and extract a quantity consistent with a theoretical mass definition, such as the pole mass [103]. However, this analysis, though theoretically well defined, still relies on the assumption that the mass in the Monte Carlo codes, used to determine the experimental acceptance, is the pole mass. Moreover, since the total cross section exhibits a quite weak dependence on the top mass, the resulting uncertainty is too large for this strategy to be really competitive. Nevertheless, the very fact that the mass determined from the cross section is in agreement with the value yielded by the template and matrix-element techniques, confirms the hint that the extracted top mass mimics the pole mass.

Another possible strategy consists of using the ${\mathrm{t}}{}{} \bar{{\mathrm{t}}{}{}}{}{}$ invariant mass in events with a hard jet (*j*), since it is an observable more sensitive to the top mass than the inclusive cross section [104]. The claim of the authors is that the unknown higher-order corrections to the ${\mathrm{t}}{}{} \bar{{\mathrm{t}}{}{}}{}{} j$ rate should contribute less than $1~\text {GeV}$ to the uncertainty on $m_{{\mathrm{t}}{}{}}$ and that the detector effects account for ${\mathcal O}(100~\mathrm{MeV})$. The ATLAS Collaboration has recently performed an analysis on the top mass extraction by using the ${\mathrm{t}}{}{} \bar{{\mathrm{t}}{}{}}{}{} j$ rate [105], along the lines of [104], where the calculation of the ${\mathrm{t}}{}{} \bar{{\mathrm{t}}{}{}}{}{} j$ cross section is performed at NLO, by using the pole top-quark mass. The result $m_t^\mathrm{pole}=[173.7\pm 1.5 (\mathrm{stat}) \pm 1.4 (\mathrm{syst})^{+1.0}_{-0.5}(\mathrm{theory})]~\text {GeV}$ is presently the most precise extraction of the pole mass.

One can also reconstruct the top mass by using the Mellin moments of lepton ($\ell ^\pm$) observables in the dilepton channel, such as $p_{{\mathrm{t}}{}{}}(\ell ^\pm )$, $p_{{\mathrm{t}}{}{}}(\ell ^+\ell ^-)$, $m_{\ell ^+\ell ^-}$, $E(\ell ^+)+E(\ell ^-)$ and $p_{{\mathrm{t}}{}{}}(\ell ^+)+p_{{\mathrm{t}}{}{}}(\ell ^-)$, which are typically linear functions of the top mass [106]. The advantage is that such observables exhibit very little dependence on showers and non-perturbative effects and do not require the reconstruction of the top quarks. The current estimate, relying on aMC@NLO [107] and MadSpin [108] for the top-quark spin correlations, is that $m_{{\mathrm{t}}{}{}}$ can be reconstructed with an error around $800~\text {MeV}$.

Future lepton facilities will be an excellent environment to measure the top mass, as it will be easier to identify top quark events than at hadron colliders. At the moment, we have several proposals for lepton colliders, mainly ${{\mathrm{e}}{}{}}^{+} {{\mathrm{e}}{}{}}^{-}$ machines: the International Linear Collider (ILC), the Compact Linear Collider (CLIC) as well as circular colliders (TLEP). The potential for top-quark physics at ILC and CLIC has been recently revisited [109], with simulations of the luminosity spectra and detector response. Top-quark analyses at both CLIC and ILC are affected by the background due to ${\upgamma }{}{} {\upgamma }{}{}$ annihilation into hadrons, which has to be reduced.

At ${{\mathrm{e}}{}{}}^{+} {{\mathrm{e}}{}{}}^{-}$ colliders, top-pair production near threshold is an interesting process, where two main contrasting effects play a role: because of the strong interaction, the ${\mathrm{t}}{}{}$ and the $\bar{{\mathrm{t}}{}{}}{}{}$ can form a Coulomb bound state, whereas the electroweak interaction smears out the peak of the cross section. The resonant cross section, computed up to NNLO accuracy [110] by using non relativistic QCD, is peaked at $\sqrt{s}\simeq 2m_{{\mathrm{t}}{}{}}$ and behaves like $\sigma _\mathrm{res}\sim \alpha _{\mathrm{s}}^3/(m_{{\mathrm{t}}{}{}}\Gamma _{{\mathrm{t}}{}{}})$; the NNNLO calculation is nowadays among the main challenges in perturbative QCD. The top mass can thus be reconstructed through a so-called threshold scan. Besides pole and $\overline{\mathrm{MS}}$ masses, a particularly suitable mass definition at threshold is the 1S mass [111] $m_{{\mathrm{t}}{}{}}^{1S}$, a short-distance mass defined as half the mass of a fictitious $^3S_1$ toponium ground state for stable top quarks.

In order to estimate the uncertainty on the measurement of the top mass at a lepton collider, a simulation scanning the range $$346~\text {GeV}<\sqrt{s}< 354~\text {GeV}$$ in steps of $$1~\text {GeV}$$, by using the TOPPIK program [111] and assuming an integrated luminosity $${\mathcal L}=300$$$$~\mathrm{fb}^{-1}$$was carried out in [112]. The overall uncertainty is gauged to be about $$100~\text {MeV}$$, after summing in quadrature the uncertainties due to statistics ($$30~\text {MeV}$$), luminosity ($$50~\text {MeV}$$), beam energy ($$35~\text {MeV}$$) and on the functional form of $$f(\sqrt{s_{\mathrm {res}}},m_t)$$ ($$80~\text {MeV}$$). The luminosity spectrum of the machine affects the (statistical) uncertainty of the measurement: passing from CLIC to ILC the uncertainty on the mass should improve by 10–20 %.

The theoretical error, due to missing higher orders and uncertainties on the quantities entering in the calculation, such as $\Gamma _{{\mathrm{t}}{}{}}$ and $\alpha _{\mathrm{s}}$, is predicted to be $3\,\%$ of the full uncertainty. Furthermore, a 2D template fit to the cross section can be performed as well, measuring simultaneously $m_{{\mathrm{t}}{}{}}$ and $\alpha _{\mathrm{s}}$. Through this method, one can reach an uncertainty on the pole $m_{{\mathrm{t}}{}{}}$ of $60~\text {MeV}$ and on the $1\mathrm {S}$ mass of $30~\text {MeV}$.

Above threshold, the top mass can still be determined by using final-state distributions, in the same manner as at hadron colliders: with $$\sqrt{s}= 500~\text {GeV}$$ and $${\mathcal L}=500$$$$~\mathrm{fb}^{-1}$$, current estimates foresee an uncertainty of $$80~\text {MeV}$$ [109].

### Top quark couplings

The determination of the coupling of the top quarks to ${\mathrm{W}}{}{}$, ${\mathrm{Z}}{}{}$ and Higgs bosons, as well as to photons and gluons, is certainly a challenge in top-quark phenomenology. In particular, possible direct measurements of the Yukawa coupling will be a crucial test of the Standard Model and will help to shed light on some new physics models.

The strong coupling constant $\alpha _{\mathrm{s}}$ can be extracted from the measurement of the ${\mathrm{t}}{}{} \bar{{\mathrm{t}}{}{}}{}{}$ and ${\mathrm{t}}{}{} \bar{{\mathrm{t}}{}{}}{}{} j$ cross sections. Ref. [103] compared the NNLO calculation [102] with the measured ${\mathrm{t}}{}{} \bar{{\mathrm{t}}{}{}}{}{}$ cross section in terms of $m_{{\mathrm{t}}{}{}}$ and $\alpha _{\mathrm{s}}(m_{{\mathrm{Z}}{}{}})$. Once the top pole mass in the computation is fixed to the world average, one can extract the strong coupling constant from the comparison, obtaining the value $\alpha _{\mathrm{s}}(m_{{\mathrm{Z}}{}{}})= 0.1151^{+0.0033}_{-0.0032}$, which is at present the first $\alpha _{\mathrm{s}}$ determination in top-quark events and within a NNLO analysis. The experimental (about $3.5\,\%$) and theory (about $5\,\%$) uncertainties are of similar order of magnitude and are not expected to change dramatically in the future LHC operation, namely centre-of-mass energy $13~\text {TeV}$ and luminosity 300 fb$^{-1}$. In future perspectives, at a linear collider, through a threshold scan of the total cross section, it will be possible to extract $\alpha _{\mathrm{s}}$ with an uncertainty smaller than $1\,\%$ and the width $\Gamma _{{\mathrm{t}}{}{}}$ with an accuracy of a few percent [112].

The coupling of the top quarks to ${\mathrm{W}}{}{}$ bosons can be measured through top decays and single-top production. The helicity fractions of ${\mathrm{W}}{}{}$ bosons in top decays have been calculated to NNLO accuracy in [113], and therefore the theory uncertainty is by far smaller than the experimental one. A higher level of precision of the measurement of such helicities, by exploiting the leptonic angular distributions, is thus mandatory in the next LHC operations, in order to test the Standard Model in the top-decay sector as well. As for single-top production, the LHC cross sections in the *s*- and ${\mathrm{t}}{}{}$-channel, as well as in the ${\mathrm{W}}{}{}{\mathrm{t}}{}{}$ associated-production mode, are in agreement with the Standard Model expectations, but are affected by rather large uncertainties (see, e.g., Refs. [114, 115] for the ${\mathrm{t}}{}{}$-channel case), with the systematic ones being even above $10\,\%$. Increasing the energy and the luminosity of the LHC will not improve too much the accuracy of this measurement, but nevertheless a precision of $5\,\%$ in the determination of the single-top cross section and of $2.5\,\%$ in the measurement of the CKM matrix element $V_{{\mathrm{t}}{}{} {\mathrm{b}}{}{}}$ is foreseen [116].

Future ${{\mathrm{e}}{}{}}^{+} {{\mathrm{e}}{}{}}^{-}$ colliders will be able to measure the ${\mathrm{t}}{}{} {\mathrm{W}}{}{}{\mathrm{b}}{}{}$ coupling with an accuracy about $2\,\%$, by scanning the centre-of-mass energy between $m_{{\mathrm{t}}{}{}}$ and $2m_{{\mathrm{t}}{}{}}$ [117]. Furthermore, a $\gamma e$ collider is predicted to have a precision reach for the ${\mathrm{t}}{}{} {\mathrm{W}}{}{}{\mathrm{b}}{}{}$ coupling between $10^{-1}$ and $10^{-2}$ [118], while an *ep* accelerator using the LHC facility at $1.3~\text {TeV}$ may aim at a sensitivity within $10^{-2}$ and $10^{-3}$ [119].

As for the top coupling to photons, although measurements of the top charge [120] and of the inclusive ${\mathrm{t}}{}{} \bar{{\mathrm{t}}{}{}}{}{} {\upgamma }{}{}$ cross section [121] are available, with the results being in agreement with the Standard Model predictions, it would be desirable determining the ${\mathrm{t}}{}{} \bar{{\mathrm{t}}{}{}}{}{} \gamma$ coupling with a higher level of precision. In fact, this process suffers from large QCD backgrounds, and it is therefore necessary to set strong cuts to suppress them; the NLO calculation for ${\mathrm{t}}{}{} \bar{{\mathrm{t}}{}{}}{}{} \gamma$ production [122] will help an improved measurement at the LHC. At $14~\text {TeV}$, with a luminosity of 300$~\mathrm{fb}^{-1}$, the coupling to photons is expected to be measured with a precision of $4\,\%$, whereas at 3000$~\mathrm{fb}^{-1}$ the expected accuracy is expected to be about $1\,\%$.

As for ${\mathrm{t}}{}{} \bar{{\mathrm{t}}{}{}}{}{} {\mathrm{Z}}{}{}$, improving the cross section measurement as well as detecting single tops in association with a ${\mathrm{Z}}{}{}$ are important challenges for the next LHC run. At 300$~\mathrm{fb}^{-1}$ the ${\mathrm{t}}{}{} \bar{{\mathrm{t}}{}{}}{}{} {\mathrm{Z}}{}{}$ axial coupling can be measured with an uncertainty of about $6\,\%$, but the vector one only with an accuracy of $50\,\%$; increasing the luminosity to 3000$~\mathrm{fb}^{-1}$should allow a determination of the vector coupling with an uncertainty of $17\,\%$ [123].

A linear collider will certainly be an ideal environment to test the coupling of top quarks with ${\upgamma }{}{}$ and ${\mathrm{Z}}{}{}$ bosons. As the ${{\mathrm{e}}{}{}}^{+} {{\mathrm{e}}{}{}}^{-} \rightarrow {\mathrm{t}}{}{} \bar{{\mathrm{t}}{}{}}{}{}$ process mixes photon and ${\mathrm{Z}}{}{}$ exchanges, having polarized beams will be fundamental to measure independently such couplings. Reference [124] studied the reach of the linear colliders ILC and CLIC, with polarizations of electrons and positrons equal to 80 and $30\,\%$, respectively, and $\sqrt{s}=500~\text {GeV}$, finding that the expected precision is at the level of permille, namely $2\times 10^{-3}$ for the coupling to photons and between $3\times 10^{-3}$ and $5\times 10^{-3}$ for ${\mathrm{t}}{}{} \bar{{\mathrm{t}}{}{}}{}{} {\mathrm{Z}}{}{}$. FCC-*ee* should be able to permit such measurements with an even better sensitivity, thanks to a higher luminosity; however, the absence of polarization will not allow to disentagle of the ${\upgamma }{}{}$ and ${\mathrm{Z}}{}{}$ contributions.

The determination of the Yukawa coupling of top quarks is clearly a crucial one, since the top-Higgs coupling provides the largest corrections to the Higgs mass at one loop, leading to the well known naturalness problem (see the discussion on the naturalness issue in Sect. 1). In order to extract the Yukawa coupling, one would need to measure the cross section of the process $pp\rightarrow {\mathrm{t}}{}{} \bar{{\mathrm{t}}{}{}}{}{} {\mathrm{H}}{}{}$: the LHC analyses at 7 and $8~\text {TeV}$ yielded upper limits on the ${\mathrm{t}}{}{} \bar{{\mathrm{t}}{}{}}{}{} {\mathrm{H}}{}{}$ cross section slightly above the Standard Model expectations [125, 126]. Measurements foreseen at 13 and $14~\text {TeV}$ should shed light on the observed excess: the expected accuracy on the ${\mathrm{t}}{}{} \bar{{\mathrm{t}}{}{}}{}{} {\mathrm{H}}{}{}$ cross section is $15\,\%$ at 300$~\mathrm{fb}^{-1}$ and $10\,\%$ at 3000$~\mathrm{fb}^{-1}$ [127].

Even better measurements of the Yukawa coupling are among the goals of lepton colliders: for an ILC of 1000 fb$^{-1}$, the foreseen accuracies are 10 % at $\sqrt{s}=500~\text {GeV}$ and $4\,\%$ at $1~\text {TeV}$, under the assumption that the polarization rates are $80\,\%$ for electrons and $30\,\%$ for positrons. As for CLIC, the note [128] investigates the potential for a direct measurement of the top Yukawa coupling. The relative uncertainty scales like $0.53\times \Delta \sigma /\sigma$, $\sigma$ being the cross section for ${\mathrm{t}}{}{} \bar{{\mathrm{t}}{}{}}{}{} {\mathrm{H}}{}{}$ production, so that, for ${{\mathrm{e}}{}{}}^{-} {{\mathrm{e}}{}{}}^{+}$ annihilation at $1.4~\text {TeV}$, a precision of $4.27\,\%$ can be achieved without beam polarization.

At FCC-*ee*, the only possible strategy to extract the Yukawa coupling is a threshold scan of the ${{\mathrm{e}}{}{}}^{+} {{\mathrm{e}}{}{}}^{-} \rightarrow {\mathrm{t}}{}{} \bar{{\mathrm{t}}{}{}}{}{}$ cross section, in order to be sensitive to Higgs exchange, besides the ${\mathrm{Z}}{}{}$ and photon contributions. The projections are about $30\,\%$, thus worse than the expectations of ILC and CLIC [129].

### Final-state kinematics

Studying kinematic distributions relying on top production and decay does provide important tests of the Standard Model and allows one to investigate several new physics scenarios. The complete differential process ${\mathrm{p}}{}{} {\mathrm{p}}{}{} \rightarrow {\mathrm{t}}{}{} \bar{{\mathrm{t}}{}{}}{}{} \rightarrow {\mathrm{W}}{}{}^+{\mathrm{b}}{}{} {\mathrm{W}}{}{}^-\bar{{\mathrm{b}}{}{}}{}{}$ has been computed to NLO accuracy, with [130, 131] and without [132] including top width effects.

Among the observables which have been investigated, the top transverse momentum spectrum has been calculated by means of resummed calculations, carried out using standard techniques [133] and in the framework of SCET [134], wherein even the ${\mathrm{t}}{}{} \bar{{\mathrm{t}}{}{}}{}{}$ invariant mass $m_{{\mathrm{t}}{}{} \bar{{\mathrm{t}}{}{}}{}{}}$ has been computed. Although such computations generally agree with the experimental data, it was found [135], by using the NLO MCFM program [136], that the uncertainty on the $p_{{\mathrm{t}}{}{}}$ spectrum in the boosted regime, i.e., the top decay products clustered into a single jet, is about twice larger than in the unboosted case. Such a result clearly calls for a full NNLO calculation in that regime.

An important final-state observable is the forward–backward asymmetry, which has represented for some time an open issue, since it exhibited a $2\sigma$ deviation at the Tevatron [137], when compared with NLO QCD predictions. However, the recent calculation [138] of the full NNLO corrections to the asymmetry, which is also the first differential NNLO computation for $2\rightarrow 2$ QCD processes, has shown agreement with the D0 data [139], whereas the disagreement with CDF [137] is reduced to 1.5 standard deviations.

At the LHC, such a measurement, which is straightforward for $${\mathrm{q}}{}{} \bar{{\mathrm{q}}{}{}}{}{} $$ initial states, is more difficult: in a $${\mathrm{p}}{}{} {\mathrm{p}}{}{} $$ collider $${\mathrm{t}}{}{} \bar{{\mathrm{t}}{}{}}{}{} $$ production is mostly driven by $${\mathrm{g}}{}{} {\mathrm{g}}{}{} $$ annihilation. In fact, ATLAS and CMS performed measurements of the asymmetry, in agreement with the Standard Model, but affected by large errors [139, 140]. Enhancing the energy to $$14~\text {TeV}$$ will increase the production of $${\mathrm{t}}{}{} \bar{{\mathrm{t}}{}{}}{}{} $$ pairs through $${\mathrm{g}}{}{} {\mathrm{g}}{}{} $$ annihilation, which does not produce any forward–backward asymmetry. However, as discussed in [135], the uncertainties due to background modelling and lepton identification scale with the luminosity as $$1/\sqrt{{\mathcal L}}$$ and therefore, after setting appropriate cuts on the $${\mathrm{t}}{}{} \bar{{\mathrm{t}}{}{}}{}{} $$ invariant mass and centre-of-mass rapidity, the fraction of $${\mathrm{q}}{}{} \bar{{\mathrm{q}}{}{}}{}{} $$ annihilation can be enhanced, thus allowing an improved measurement of the asymmetry. Two alternatives to the standard forward–backward asymmetry have been proposed in [141] in events with $${\mathrm{t}}{}{} \bar{{\mathrm{t}}{}{}}{}{} $$+jet: they are the energy and incline asymmetries, expressed in terms of the energy difference between the $${\mathrm{t}}{}{} $$ and the $$\bar{{\mathrm{t}}{}{}}{}{} $$ and of the rapidity of the $${\mathrm{t}}{}{} \bar{{\mathrm{t}}{}{}}{}{} j$$ system. After setting suitable cuts, the incline-asymmetry distribution, evaluated at NLO in QCD in [141], can reach the value of $$-4\,\%$$ at $$14~\text {TeV}$$ LHC, and can be observed with a significance of 5 standard deviations at a luminosity of 100$$~\mathrm{fb}^{-1}$$. As for the energy-asymmetry distribution, its maximum value at $$14~\text {TeV}$$ is $$-12\,\%$$ and it can be measured at $$\mathcal{L}=100$$$$~\mathrm{fb}^{-1}$$with a significance of 3$$\sigma $$.

At a linear collider, the main kinematic properties which are foreseen to be measured are the top production angle $\theta _{{\mathrm{t}}{}{}}$ and the helicity angle $\theta _h$. In this way, one will be able to determine the forward–backward asymmetry and the slope of the helicity angle $\lambda _{{\mathrm{t}}{}{}}$ with an accuracy of 2 % in semileptonic events, as obtained in the simulations at $\sqrt{s}=500~\text {GeV}$ carried out in [142]. In the ${\mathrm{t}}{}{} \bar{{\mathrm{t}}{}{}}{}{}$ threshold regime, where a number of measurements at the linear collider is planned, at present only the total cross section has been computed at NNLO, whereas the calculation of NNLO differential distributions is highly desirable, in order to take full advantage of such a machine.

## Effective field theories for the Higgs sector

### Introduction

The discovery by the ATLAS and CMS[81, 82] collaborations of a scalar boson with mass $m_{{\mathrm{H}}{}{}} \simeq 125~\text {GeV}$, has prompted unprecedented theoretical and experimental activities to accurately determine its properties, especially the strength and the structure of the coupling to the other Standard Model (SM) particles. Even though the present measurements point to production cross section and decay rates compatible with those predicted for the Higgs boson of the SM, the uncertainties are still quite large, i.e., at the level of 10–20 %. One of the aims of the next LHC runs and possibly of future linear or circular colliders, is therefore to bring down these uncertainties to the percent level [127].

This program highlights the need for a framework to systematically organize precision tests of the SM and to parametrize its plausible deformations. Here we argue that a SM EFT provides such a framework.

The essence of a bottom-up EFT approach is that, since no new physics has been observed at the LHC, we can assume that it is much heavier than the energy accessible to our experiments and expand the Lagrangian in powers of energy (derivatives) over the new physics scale, $D_\mu /\Lambda$ and in powers of (SM) fields.^2^ In this way one builds an effective description by systematically adding to the Lagrangian of the SM all possible higher-dimensional operators compatible with the SM $SU(3) \times SU(2) \times U(1)$ symmetries and containing only SM fields, see e.g.  [22, 143–157]. It is important to notice that the expansion in fields lies on a different footing w.r.t. the expansion in derivatives. In fact, while the former is necessarily associated with inverse powers of the mass-scale $\Lambda$, the latter must also involve a coupling, which we generically call $g_*$ (this is easily seen by restoring powers of $\hbar$ in the Lagrangian: since couplings scale $[g_*]\sim \hbar ^{-1/2}$ while fields scale as $[H]\sim \hbar ^{1/2}$, the genuine dimensionless building block of the Lagrangian is, e.g., $g_*H/\Lambda$). For this reason the effective description is valid as long as new physics states appear at a scale $\Lambda \gg E$ much larger than the scale at which experiments are performed (e.g $\Lambda \gg m_{{\mathrm{H}}{}{}}$), *and* as long as $\Lambda \gg g_*v$. It is worth noting that there are contexts where the former expansion is good, while this latter expansion fails, corresponding to scenarios where the BSM is directly responsible for EWSB, as in Technicolor models, and a description in terms non-linearly realized EW symmetry becomes more appropriate. In these contexts the leading-order (dimension-4) Lagrangian does not coincide with the SM, since the 125 GeV scalar has no relation with the Higgs boson and, considering that all observations made by the LHC experiments so far are in good agreement with SM predictions, it is natural to consider this option as disfavored. Moreover, if the underlying theory respects custodial symmetry and one considers only observables which involve the same number of Higgs particles, the effective description is the same, independently on whether or not we perform the expansion in powers of the Higgs field. For these reasons we will assume the validity of the expansion in fields or, equivalently, we assume that the observed Higgs scalar is part of an $SU(2)_{\mathrm L}$ doublet together with the longitudinal polarizations of the *W*, *Z* bosons.

Under this assumption the effective Lagrangian can be expanded into a sum of operators with increasing dimensionality, with only one operator of dimension five (the one associated to the Majorana mass of the neutrinos) and a set of 76 operators at dimension six (for one fermion family, counting real independent parameters in the effective Lagrangian) [152].

The use of an EFT approach brings significant advantages, above all with respect to alternative parametrizations, such as those based on generic anomalous couplings [158]. First of all, EFTs represent a consistent and flexible framework to perform precision tests of the SM, where radiative corrections can be rigorously incorporated, different assumptions (e.g. custodial symmetry, flavor symmetries, ...) can be independently tested, and it is easily and systematically improvable. Secondly, expressing precision SM tests in terms of EFTs allows us to interpret the results in terms of physics beyond the SM (BSM) in a generalization of the popular *S*, *T* parameters. This relation represents a channel to compare precision searches with direct searches and also provides one simple but important motivation for performing precision tests: if new physics resides at a scale $$\Lambda $$ and couples to the Higgs field with strength $$g_*$$, at low energy it might only induce a relative change of order $$\sim $$$$ (g_* v/\Lambda )^2$$ to some couplings, where $$v=246~\text {GeV}$$ is the Higgs vacuum expectation value. For maximally strongly coupled theories with $$g_*\lesssim 4\pi $$, a 10 % (1 %) deviation from the SM would correspond to a new physics scale $$\Lambda \sim 10 \, (40) ~\text {TeV}$$, unreachable with direct searches. Finally, another motivation for EFTs is that they provide an educated principle to organize deformations from the SM into a leading/next-to-leading hierarchy (corresponding to increasingly higher orders in the EFT expansion parameters) and, moreover, such a hierarchical scheme reflects in an (almost) generic way the low-energy behavior of large classes of BSM scenarios. This model-independence represents one further advantage of precision tests (in the form of precision searches) over direct searches for New Physics, that typically require a concrete model to extract the most out of them. Furthermore, the breakdown of the EFT description at energies $$E\simeq \Lambda $$ provides an important self-consistency check: issues such as unitarity violation (which is a major problem of any anomalous coupling description), are automatically taken into account by the EFT [159, 160]. can be clearly identified and analysed in the context of an EFT [159, 160].

The Lagrangian of the SM+higher-dimensional operators is renormalizable à la Wilson. In other words, order by order in $\Lambda$, higher order corrections in the couplings can be consistently computed. Moreover, in principle, the inclusion of higher-order $E^n/\Lambda ^n$ effects to a given observable (measured at energies $E<\Lambda$), allows to consistently incorporate BSM effects with higher and higher accuracy. All this is essential in the extraction of information from cross section measurements at hadron colliders where higher-order QCD effects are always relevant and at ${{\mathrm{e}}{}{}}^{+} {{\mathrm{e}}{}{}}^{-}$-colliders, where the precision is so high that SM EW corrections become important. An example of the utility of such a parametrization, and the importance of being able to include EW corrections, is given by the popular S,T precision parameters [161], that represent a subset of the EFT parametrization suitable for universal (i.e. where the new physics couples only to gauge bosons) BSM theories [162].

The more general EFT contains operators that affect EW precision observables as well as operators that affect Higgs physics, and, since in the SM the Higgs excitation is always accompanied by the Higgs vev, $v+h$, some operators contribute to both Higgs and EW physics. The latter can therefore be strongly constrained independently of Higgs physics. So even though the number of free parameters in the EFT seems quite large at face value, it is possible that by identifying a suitable set of observables to constrain all of them *at the same time*, by performing a global fit. Work in this direction has already started [163–167], but unexplored avenues remain, in particular in the relation between flavor observables in Higgs and non-Higgs processes.

In summary, the EFT provides a consistent and systematically improvable framework to quantify and interpret deviations from the Standard Model predictions due to physics residing at higher scales, $\Lambda$, not only in Higgs physics but for all SM particles and interactions. The key questions that we would like to address are: What are the prospects to precisely determine the Higgs couplings and parametrise possible deviations in terms of an EFT in the coming LHC runs and possibly beyond? What are the current and foreseeable theoretical and experimental challenges in pursuing a precise determination of all the parameters entering dim $=$ 6 SM Lagragian, in particular for the part concerning the Higgs?

The plan of this contribution is as follows. In the following section, the basic features and properties of the Higgs EFT’s reviewed, with the aim of clarifying the main points and presenting the state-of-the-art. In Sect. 4.3 the results of the Snowmass study are summarised. In Sect. 4.4 the main directions of theoretical and experimental activity where significant work is expected to meet the required accuracy and precision are highlighted.

### The dim $=$ 6 Standard Model Lagrangian

We start from the $SU(3)_c\times SU(2)_{\mathrm L}\times U(1)_Y$ gauge symmetry of the SM. The gauge vector fields lie in the adjoint representation of the relevant gauge subgroup,

1 $$\begin{aligned} SU(3)_c\rightarrow & {} G^a_\mu = ({\mathbf{8}},{\mathbf{1}},0) ,\quad SU(2)_{\mathrm L} \rightarrow {\mathrm{W}}{}{}^k_\mu = ({\mathbf{1}},{\mathbf{3}},0) \nonumber \\ U(1)_{\mathrm Y}\rightarrow & {} {\mathrm{B}}{}{} _\mu = ({\mathbf{1}},{\mathbf{1}},0). \end{aligned}$$

The chiral matter content of the theory is organized in three generations of left-handed and right-handed quark (${\mathrm{q}}{}{} _{\mathrm L}$, ${\mathrm{u}}{}{} _{\mathrm R}$ and ${\mathrm{d}}{}{} _{\mathrm R}$) and lepton ($L_{\mathrm L}$ and $e_{\mathrm R}$) fields (we ignore neutrino masses in this context),

2 $$\begin{aligned} {\mathrm{q}}{}{} _{\mathrm L}= & {} \begin{pmatrix}{\mathrm{u}}{}{} _{\mathrm L}\\ {\mathrm{d}}{}{} _{\mathrm L} \end{pmatrix}= \left( \mathbf{3}, \mathbf{2},\frac{1}{6}\right) , \quad {\mathrm{u}}{}{} _{\mathrm R} = \left( \mathbf{3}, \mathbf{1}, \frac{2}{3}\right) , \quad \nonumber \\ {\mathrm{d}}{}{} _{\mathrm R}= & {} \left( \mathbf{3}, \mathbf{1},-\frac{1}{3}\right) ,\\ L_{\mathrm L}= & {} \begin{pmatrix}\nu _{\mathrm L} \\ \ell _{\mathrm L} \end{pmatrix}= \left( \mathbf{1}, \mathbf{2},-\frac{1}{2}\right) , \quad e_{\mathrm R} = (\mathbf{1}, \mathbf{1},-1).\nonumber \end{aligned}$$

Finally, the scalar sector contains a single $SU(2)_{\mathrm L}$ doublet of fields,

3 $$\begin{aligned} \Phi = \begin{pmatrix}-i G^+ \\ \frac{1}{\sqrt{2}} \Big [ v + h + i G^0\Big ] \end{pmatrix}= \left( \mathbf{1}, \mathbf{2},\frac{1}{2}\right) . \end{aligned}$$

With the first equality, we show the component fields of the doublet after shifting the neutral field *h* by its vacuum expectation value *v*. Moreover, we have included the Goldstone bosons $G^{+,0}$ to be absorbed by the weak bosons to get their longitudinal degree of freedom.

In the EFT approach that we adopt, the SM is defined as the leading part (including relevant and marginal operators) of an expansion in fields and derivatives, while new interactions possibly due to non-observed heavy states, at a scale of order $\Lambda \gtrsim m_{{\mathrm{W}}{}{}}$, are parametrized by operators of higher dimension. Ignoring interactions of dimension 5, that lead to Majorana masses for the neutrinos, the next-to-leading terms in this expansion come from operators of dimension six. Here we focus on operators that contain the Higgs doublet, so that they can potentially be relevant for Higgs physics. In a convenient basis of independent operators ${\mathcal O}_i$ [151, 152, 155, 163, 166, 168] these can be written as

4 $$\begin{aligned} {\mathcal L}= & {} {\mathcal L}_{\mathrm {SM}} + \sum _i \bar{c}_i {\mathcal O}_i, \nonumber \\= & {} {\mathcal L}_{\mathrm {SM}} + {\mathcal L}_{\text {H-only}} + {\mathcal L}_{\mathrm {EW}}+ {\mathcal L}_{\mathrm {CP}} + {\mathcal L}_{\mathrm {dip}} + {\mathcal L}_{\text {no-Higgs}},\nonumber \\ \end{aligned}$$

assuming baryon and lepton number conservation (at the scales relevant for Higgs physics).

${\mathcal L}_{\text {H-only}}$ corresponds to the set of *CP*-conserving operators that contain the Higgs doublet appearing as $\Phi ^\dagger \Phi$:

5 $$\begin{aligned}&\Delta {\mathcal L}_{\text {H-only}} = \, \frac{\bar{c}_\Phi }{2v^2}\, \partial ^\mu \!( \Phi ^\dagger \Phi ) \partial _\mu \!( \Phi ^\dagger \Phi ) - \frac{\bar{c}_6\, \lambda }{v^2}( \Phi ^\dagger \Phi )^3 \nonumber \\&\quad + \left[ \left( \frac{\bar{c}_u}{v^2}\, y_{u}\, \Phi ^\dagger \Phi \, {\bar{{\mathrm{q}}{}{}}{}{}}_{\mathrm L} \Phi ^c {\mathrm{u}}{}{} _{\mathrm R} + \frac{\bar{c}_d}{v^2}\, y_{d}\, \Phi ^\dagger \Phi \, {\bar{{\mathrm{q}}{}{}}{}{}}_{\mathrm L} \Phi {\mathrm{d}}{}{} _{\mathrm R} \right. \right. \nonumber \\&\quad \left. \left. + \frac{\bar{c}_l}{v^2}\, y_{l}\, \Phi ^\dagger \Phi \, {\bar{L}}_{\mathrm L} \Phi l_{\mathrm R} \right) + { h.c.} \right] \nonumber \\&\quad +\frac{\bar{c}_{BB}\, {g'}^2}{4m_{{\mathrm{W}}{}{}}^2}\, \Phi ^\dagger \Phi {\mathrm{B}}{}{} _{\mu \nu }{\mathrm{B}}{}{} ^{\mu \nu } +\frac{\bar{c}_{WW} \, g^2}{4m_{{\mathrm{W}}{}{}}^2}\, \Phi ^\dagger \Phi {\mathrm{W}}{}{}_{\mu \nu }^a {\mathrm{W}}{}{}^{a\mu \nu }\nonumber \\&\quad +\frac{\bar{c}_{GG} \, g_{\mathrm S}^2}{4m_{{\mathrm{W}}{}{}}^2}\, \Phi ^\dagger \Phi G_{\mu \nu }^a G^{a\mu \nu } \end{aligned}$$

where the Wilson coefficients $\bar{c}$ are real free parameters, $\lambda$ stands for the Higgs quartic coupling and $y_u$, $y_d$ and $y_\ell$ are the $3\times 3$ Yukawa coupling matrices in flavor space (all flavor indices are understood for clarity). In this expression, we also denote the $U(1)_{\mathrm Y}$, $SU(2)_{\mathrm L}$ and $SU(3)_c$ coupling constants by $g'$, *g* and $g_s$, and ${\mathrm{q}}{}{} _{\mathrm L}\cdot \Phi = \epsilon _{ij}\ {\mathrm{q}}{}{} _{\mathrm L}^i\ \Phi ^j \quad \text {and}\quad \Phi ^\dag \cdot \bar{{\mathrm{q}}{}{}}{}{} _{\mathrm L} = \epsilon ^{ij}\ \Phi ^\dag _i\ \bar{{\mathrm{q}}{}{}}{}{} _{Lj}$ and $\Phi ^c\equiv \epsilon ^{ij} \Phi ^{*}_j$, with the rank-two antisymmetric tensors being defined by $\epsilon _{12}=1$ and $\epsilon ^{12}=-1$. Finally, our conventions for the gauge-covariant derivatives and the gauge field strength tensors are

6 $$\begin{aligned} {\mathrm{B}}{}{} _{\mu \nu }= & {} \partial _\mu {\mathrm{B}}{}{} _\nu - \partial _\nu {\mathrm{B}}{}{} _\mu , \nonumber \\ {\mathrm{W}}{}{}^k_{\mu \nu }= & {} \partial _\mu {\mathrm{W}}{}{}^k_\nu - \partial _\nu {\mathrm{W}}{}{}^k_\mu + g \epsilon _{ij}{}^k \ {\mathrm{W}}{}{}^i_\mu {\mathrm{W}}{}{}^j_\nu ,\nonumber \\ G^a_{\mu \nu }= & {} \partial _\mu G^a_\nu - \partial _\nu G^a_\mu + g_s f_{bc}{}^a\ G^b_\mu G^c_\nu ,\nonumber \\ D_\rho {\mathrm{W}}{}{}^k_{\mu \nu }= & {} \partial _\mu \partial _\rho {\mathrm{W}}{}{}^k_\nu - \partial _\nu \partial _\rho {\mathrm{W}}{}{}^k_\mu + g \epsilon _{ij}{}^k \partial _\rho [{\mathrm{W}}{}{}_\mu ^i {\mathrm{W}}{}{}_\nu ^j] \nonumber \\&+ g \epsilon _{ij}{}^k {\mathrm{W}}{}{}_\rho ^i [\partial _\mu {\mathrm{W}}{}{}_\nu ^j - \partial _\nu {\mathrm{W}}{}{}_\mu ^j]\nonumber \\&+ g^2 {\mathrm{W}}{}{}_{\rho i}[{\mathrm{W}}{}{}_\nu ^i {\mathrm{W}}{}{}_\mu ^k - {\mathrm{W}}{}{}_\mu ^i {\mathrm{W}}{}{}_\nu ^k] , \nonumber \\ D_\mu \Phi= & {} \partial _\mu \Phi - \frac{1}{2} i g' {\mathrm{B}}{}{} _\mu \Phi - i g T_{2k} {\mathrm{W}}{}{}_\mu ^k \Phi , \end{aligned}$$

$\epsilon _{ij}{}^k$ and $f_{ab}{}^c$ being the structure constants of *SU*(2) and *SU*(3). Notice that we have normalized the Wilson coefficients using SM scales and couplings (following the discussion above), which is equivalent to absorbing powers of $m_{{\mathrm{W}}{}{}}/\Lambda$ or $g_*v/\Lambda$ inside the Wilson coefficient: in this way (since the relevant experiments are performed at energies $E\sim m_{{\mathrm{W}}{}{}}$) we can easily keep track of the validity of the perturbative expansion by requiring that $1\gg c_i$.

The interesting feature about Eq. (5) is that it contains effects that can be studied only in physics that involves the physical Higgs particle *h*. In fact, in the vacuum $\Phi \rightarrow v$, the effect of Eq. (5) can absorbed into a redefinition of the SM parameters [155, 163]. The Wilson coefficients $\bar{c}$ of Eq. (5) can, at leading order, be mapped one-to-one with observables in the context of Higgs physics, in particular

7 $$\begin{aligned} {\kappa _{{\mathrm{u}}{}{}},\kappa _{{\mathrm{d}}{}{}},\kappa _{{\mathrm{l}}{}{}},\kappa _{{\mathrm{V}}{}{}},\kappa _{{\upgamma }{}{}},\kappa _{{\mathrm{g}}{}{}},\kappa _{{\mathrm{Z}}{}{}{\upgamma }{}{}}}, \end{aligned}$$

which have already been the subject of LHC Run1 experiments, and the Higgs self coupling $\kappa _{h^3}$, which will be measured during the next Run; (we denote $\kappa _{{\mathrm{V}}{}{}}=\kappa _{{\mathrm{Z}}{}{}}= \kappa _{{\mathrm{W}}{}{}}$). We discuss these couplings in the next section.

Contrary to Eq. (5), other operators involving the Higgs field also affect EW observables and are therefore already constrained by other experiments. In particular

8 $$\begin{aligned} \Delta {\mathcal L}_{\mathrm {EW}}= & {} \frac{i\bar{c}_{{\mathrm{W}}{}{}}\, g}{2m_{{\mathrm{W}}{}{}}^2}( \Phi ^\dagger \sigma ^i \overleftrightarrow {D^\mu } \Phi )( D^\nu {\mathrm{W}}{}{}_{\mu \nu })^i \nonumber \\&+\frac{i\bar{c}_B\, g'}{2m_{{\mathrm{W}}{}{}}^2}( \Phi ^\dagger \overleftrightarrow {D^\mu } \Phi )( \partial ^\nu {\mathrm{B}}{}{} _{\mu \nu })\nonumber \\&\times \frac{\bar{c}_T}{2v^2}(\Phi ^\dagger {\overleftrightarrow { D^\mu }} \Phi ) \!( \Phi ^\dagger {\overleftrightarrow D}_\mu \Phi ) \nonumber \\&+\frac{\bar{c}_{WB}g g^\prime }{4 m_{{\mathrm{W}}{}{}}^2} \, \Phi ^\dagger \sigma ^i \Phi \, {\mathrm{W}}{}{}_{\mu \nu }^i {\mathrm{B}}{}{} ^{\mu \nu } \nonumber \\&\times \frac{i \bar{c}_{Hq}}{v^2} (\bar{{\mathrm{q}}{}{}}{}{} _{\mathrm L} \gamma ^\mu {\mathrm{q}}{}{} _{\mathrm L}) ( \Phi ^\dagger {\overleftrightarrow D}_\mu \Phi ) \nonumber \\&+ \frac{i \bar{c}^\prime _{Hq}}{v^2} (\bar{{\mathrm{q}}{}{}}{}{} _{\mathrm L} \gamma ^\mu \sigma ^i {\mathrm{q}}{}{} _{\mathrm L}) (\Phi ^\dagger \sigma ^i {\overleftrightarrow D}_\mu \Phi ) \nonumber \\&+ \frac{i \bar{c}_{Hu}}{v^2} (\bar{{\mathrm{u}}{}{}}{}{} _{\mathrm R} \gamma ^\mu {\mathrm{u}}{}{} _{\mathrm R}) ( \Phi ^\dagger {\overleftrightarrow D}_\mu \Phi )\nonumber \\&+ \frac{i \bar{c}_{Hd}}{v^2} (\bar{{\mathrm{d}}{}{}}{}{} _{\mathrm R} \gamma ^\mu {\mathrm{d}}{}{} _{\mathrm R}) ( \Phi ^\dagger {\overleftrightarrow D}_\mu \Phi ) \nonumber \\&+\left( \frac{i \bar{c}_{Hud}}{v^2} (\bar{{\mathrm{u}}{}{}}{}{} _{\mathrm R} \gamma ^\mu {\mathrm{d}}{}{} _{\mathrm R}) ( \Phi ^{c\, \dagger } {\overleftrightarrow D}_\mu \Phi ) +{ h.c.} \right) \nonumber \\&+ \frac{i \bar{c}_{HL}}{v^2} (\bar{L}_{\mathrm L} \gamma ^\mu L_{\mathrm L}) ( \Phi ^\dagger {\overleftrightarrow D}_\mu \Phi )\nonumber \\&+ \frac{i \bar{c}^\prime _{HL}}{v^2} (\bar{L}_{\mathrm L} \gamma ^\mu \sigma ^i L_{\mathrm L}) (\Phi ^\dagger \sigma ^i {\overleftrightarrow D}_\mu \Phi )\nonumber \\&+ \frac{i \bar{c}_{Hl}}{v^2} (\bar{l}_{\mathrm R} \gamma ^\mu l_{\mathrm R}) ( \Phi ^\dagger {\overleftrightarrow D}_\mu \Phi ) , \end{aligned}$$

beside modifying Higgs physics, they also contribute to precision observables, such as those measured at LEP; here $\sigma _k$ are the Pauli matrices and we have introduced the Hermitian derivative operators ${\overleftrightarrow D}_\mu$ defined as

9 $$\begin{aligned} \Phi ^\dag {\overleftrightarrow D}_\mu \Phi = \Phi ^\dag D^\mu \Phi - D_\mu \Phi ^\dag \Phi . \end{aligned}$$

Notice that two of the operators that have been introduced are redundant and can be removed through [169]

10 $$\begin{aligned} {\mathcal O}_{\scriptscriptstyle W}= & {} -2 {\mathcal O}_{\scriptscriptstyle H} + \frac{4}{v^2} \Phi ^\dag \Phi D^\mu \Phi ^\dag D_\mu \Phi + {\mathcal O}'_{\scriptscriptstyle HQ} + {\mathcal O}'_{\scriptscriptstyle HL} , \nonumber \\ {\mathcal O}_{\scriptscriptstyle B}= & {} 2 \tan ^2\theta _{\scriptscriptstyle W} \left[ \sum \limits _\psi Y_\psi {\mathcal O}_{\scriptscriptstyle H\psi } - {\mathcal O}_{\scriptscriptstyle T} \right] , \end{aligned}$$

where we sum over the whole chiral content of the theory and $\theta _{\scriptscriptstyle W}$ stands for the weak mixing angle. Once this redundancy is accounted for, all Wilson coefficients entering Eq. (8) (at least the flavor-diagonal component) can be constrained using data from *Z*-pole observables at LEP1 or information from ${{\mathrm{e}}{}{}}^{+} {{\mathrm{e}}{}{}}^{-} \rightarrow {\mathrm{W}}{}{}^+{\mathrm{W}}{}{}^-$ at LEP2, [163, 165–167].

Similarly the operators in ${\mathcal L}_{\mathrm {dip}}$ are measured both in Higgs physics and electric dipole moments (EDMs),

11 $$\begin{aligned} \Delta {\mathcal L}_{\mathrm {dip}}&= \frac{\bar{c}_{uB}\, g'}{m_{{\mathrm{W}}{}{}}^2}\, y_u \, {\bar{{\mathrm{q}}{}{}}{}{}}_{\mathrm L} \Phi ^c \sigma ^{\mu \nu } {\mathrm{u}}{}{} _{\mathrm R} \, {\mathrm{B}}{}{} _{\mu \nu }\nonumber \\&\quad + \frac{\bar{c}_{uW}\, g}{m_{{\mathrm{W}}{}{}}^2}\, y_u \, {\bar{{\mathrm{q}}{}{}}{}{}}_{\mathrm L} \sigma ^i \Phi ^c \sigma ^{\mu \nu } {\mathrm{u}}{}{} _{\mathrm R} \, {\mathrm{W}}{}{}_{\mu \nu }^i \nonumber \\&\quad + \frac{\bar{c}_{uG}\, g_{\mathrm S}}{m_{{\mathrm{W}}{}{}}^2}\, y_u \, {\bar{{\mathrm{q}}{}{}}{}{}}_{\mathrm L} \Phi ^c \sigma ^{\mu \nu } \lambda ^a {\mathrm{u}}{}{} _{\mathrm R} \, G_{\mu \nu }^a \nonumber \\&\quad + \frac{\bar{c}_{dB}\, g'}{m_{{\mathrm{W}}{}{}}^2}\, y_d \, {\bar{{\mathrm{q}}{}{}}{}{}}_{\mathrm L} \Phi \sigma ^{\mu \nu } {\mathrm{d}}{}{} _{\mathrm R} \, {\mathrm{B}}{}{} _{\mu \nu } \nonumber \\&\quad + \frac{\bar{c}_{dW}\, g}{m_{{\mathrm{W}}{}{}}^2}\, y_d \, {\bar{{\mathrm{q}}{}{}}{}{}}_{\mathrm L} \sigma ^i \Phi \sigma ^{\mu \nu } {\mathrm{d}}{}{} _{\mathrm R} \, {\mathrm{W}}{}{}_{\mu \nu }^i \nonumber \\&\quad + \frac{\bar{c}_{dG}\, g_{\mathrm S}}{m_{{\mathrm{W}}{}{}}^2}\, y_d \, {\bar{{\mathrm{q}}{}{}}{}{}}_{\mathrm L} \Phi \sigma ^{\mu \nu } \lambda ^a {\mathrm{d}}{}{} _{\mathrm R} \, G_{\mu \nu }^a \nonumber \\&\quad + \frac{\bar{c}_{lB}\, g'}{m_{{\mathrm{W}}{}{}}^2}\, y_l \, {\bar{L}}_{\mathrm L} \Phi \sigma ^{\mu \nu } l_{\mathrm R} \, {\mathrm{B}}{}{} _{\mu \nu }\nonumber \\&\quad + \frac{\bar{c}_{lW}\, g}{m_{{\mathrm{W}}{}{}}^2}\, y_l \, {\bar{L}}_{\mathrm L} \sigma ^i \Phi \sigma ^{\mu \nu } l_{\mathrm R} \, {\mathrm{W}}{}{}_{\mu \nu }^i + {h.c.} \end{aligned}$$

with complex coefficients (where real and imaginary part correspond respectively to CP-even and CP-odd effects). Other contributions from CP-violating physics BSM involving the Higgs are captured by

12 $$\begin{aligned} {\mathcal L}_{\mathrm {CP}}= & {} \left[ \left( i \frac{\tilde{c}_{ u}}{v^2}\, y_{u}\, \Phi ^\dagger \Phi \, {\tilde{q}}_{\mathrm L} \Phi ^c {\mathrm{u}}{}{} _{\mathrm R} + i \frac{\tilde{c}_d}{v^2}\, y_{d}\, \Phi ^\dagger \Phi \, {\bar{{\mathrm{q}}{}{}}{}{}}_{\mathrm L} \Phi {\mathrm{d}}{}{} _{\mathrm R} \right. \right. \nonumber \\&\left. \left. + i\frac{\bar{c}_l}{v^2}\, y_{l}\, \Phi ^\dagger \Phi \, {\bar{L}}_{\mathrm L} \Phi l_{\mathrm R} \right) + { h.c.} \right] \nonumber \\&+\frac{\tilde{c}_{WB}g g^\prime }{4 m_{{\mathrm{W}}{}{}}^2} \, \Phi ^\dagger \sigma ^i \Phi \, {\mathrm{W}}{}{}_{\mu \nu }^i \widetilde{\mathrm{B}}{}{} ^{\mu \nu } + \frac{g'^2\ \tilde{c}_{\scriptscriptstyle BB}}{4m_{{\mathrm{W}}{}{}}^2} \Phi ^\dag \Phi {\mathrm{B}}{}{} _{\mu \nu } {\widetilde{B}}^{\mu \nu }\nonumber \\&+\! \frac{g_s^2\ \tilde{c}'_{\scriptscriptstyle g}}{4m_{{\mathrm{W}}{}{}}^2} \Phi ^\dag \Phi G_{\mu \nu }^a {\widetilde{G}}^{\mu \nu }_a+\frac{g^2\ \tilde{c}'_{\scriptscriptstyle g}}{4m_{{\mathrm{W}}{}{}}^2} \Phi ^\dag \Phi {\mathrm{W}}{}{}_{\mu \nu }^a {\widetilde{W}}^{\mu \nu }_a, \end{aligned}$$

where the dual field strength tensors are defined by

13 $$\begin{aligned} \widetilde{\mathrm{B}}{}{} _{\mu \nu }= & {} \frac{1}{2} \epsilon _{\mu \nu \rho \sigma } {\mathrm{B}}{}{} ^{\rho \sigma } , \quad \widetilde{\mathrm{W}}{}{}_{\mu \nu }^k = \frac{1}{2} \epsilon _{\mu \nu \rho \sigma } {\mathrm{W}}{}{}^{\rho \sigma k} , \nonumber \\&\widetilde{G}_{\mu \nu }^a =\frac{1}{2} \epsilon _{\mu \nu \rho \sigma } G^{\rho \sigma a} . \end{aligned}$$

and the coefficients are real. The assumption of one-family in flavor space can easily be abandoned by promoting the Wilson coefficients of the fermionic operators to tensors in flavor space.

**Table 5:** Estimations by ATLAS and CMS of the expected relative precisions on the signal strengths in different Higgs decay final states. In the last column the $95\,\%$ CL upper limit on the Higgs branching ratio to the invisible decay from the ${\mathrm{Z}}{}{}{\mathrm{H}}{}{}$ search is given. ATLAS and CMS ranges are not directly comparable due to the different treatment of the expected theoretical uncertainties. Table taken from Ref. [127]

$\int {\mathcal L}dt$ (fb$^{-1}$)	Higgs decay final state							
	${\upgamma }{}{} {\upgamma }{}{}$ (%)	${\mathrm{W}}{}{}{\mathrm{W}}{}{}^*$ (%)	${\mathrm{Z}}{}{}{\mathrm{Z}}{}{}^*$ (%)	${\mathrm{b}}{}{} \bar{{\mathrm{b}}{}{}}{}{}$ (%)	$\tau \tau$ (%)	$\mu \mu$ (%)	${\mathrm{Z}}{}{}{\upgamma }{}{}$ (%)	BR$_\mathrm{inv}$ (%)
ATLAS								
300	9–14	8–13	6–12	N/A	16–22	38–39	145–147	$<$23–32
3000	4–10	5–9	4–10	N/A	12–19	12–15	54–57	$<$8–16
CMS								
300	6–12	6–11	7–11	11–14	8–14	40–42	62–62	$<$17–28
3000	4–8	4–7	4–7	5–7	5–8	14–20	20–24	$<$6–17

So, contrary to the operators in ${\mathcal L}_{\text {H-only}}$, the ones of ${\mathcal L}_{\mathrm {EW}}+ {\mathcal L}_{\mathrm {CP}} + {\mathcal L}_{\mathrm {dip}}$ have not yet received attention in the context of Higgs physics. Although they are already constrained by other experiments, it is not clear whether, in some cases, Higgs physics could lead to a higher sensitivity (see e.g. Ref. [160] for an example).

Finally, the complete dimension-6 EFT Lagrangian also includes many operators that do not contain the Higgs, such as four-fermion interactions and operators involving three field strengths. Although these do not contain the Higgs field, some of them might interfere in the extraction of constraints for the operators mentioned here (in particular the operator $\frac{g^3\ \bar{c}'_{\scriptscriptstyle 3W}}{\Lambda ^2} \epsilon _{ijk} {\mathrm{W}}{}{}_{\mu \nu }^i {\mathrm{W}}{}{}^\nu {}^j_\rho {\mathrm{W}}{}{}^{\rho \mu k}$ enters measurements of triple gauge couplings and it might reduce the sensitivity to operators involving the Higgs [167]).

It is important to stress that the basis we proposed is not unique, as highlighted by Eq. (10). Field redefinitions proportional to the leading-order equations of motion, and integration by parts can be used to express some of the operators above in terms of others, whose physical interpretation might be slightly different. This redundancy is a feature of higher-dimensional operators that is unfamiliar from the Standard Model. Because of this redundancy, there is a great deal of flexibility in which set of operators to use. In principle, any set of independent operators constitutes a good basis and there is no physically preferred basis, as long as all operators are included in the analysis. However a particular experimental measurement generally depends on only a few of the operators (at any finite order in perturbation theory) in a given basis and it might be the case that the relation between operators and observables might be simpler in one basis than in others. In this sense the basis of *BSM primaries* [166, 168] was designed to minimize the theoretical correlation between operators and provides an almost one-to-one correspondence between operators and observables. On the other hand the *SILH* basis of Refs. [22, 151, 155, 163] is more BSM oriented and is easily matched to universal microscopic models (including SUSY and Composithe Higgs models), while the basis of Ref. [152] might be more suitable to describe UV models where the BSM couples to fermions. Now, in several instances in the literature, bounds on the coefficient of a particular operator have been put by assuming that all the other operators in that basis have vanishing coefficients: this is an ad hoc and meaningless assumption, since typically no BSM scenario gives rise to a single operator (in this sense the analyses of. e.g. Refs. [25, 151] provide an educated guess of how certain patterns of Wilson coefficients might arise from general classes of BSM models). In the absence of an underlying theory, one should always include every dimension-six operator that contributes to the calculation of a physical process and each experimental measurement will generally bound a set of dimension-six operators. Constraining the Wilson coefficients implies adopting a global approach where a sufficiently comprehensive set of observables is mapped onto the full set of operators in the EFT.

### Expected precision on the couplings strength: the Snowmass study

A first useful starting point to assess the reach of the next LHC runs and possibly at future accelerators in the determination of the Higgs couplings is studying the precision in searching for deviations in the strength of the couplings [170], which corresponds to the operators of Eq. (5) through the parameters mentioned in Eq. (7). Such a study has been completed in the Snowmass workshop in summer 2013 [127].

It is important to recall the simplified working assumptions when extrapolating in luminosity. First, the structure of the coupling is the same as of the SM and only the normalisations are let free to float and determined by a global fit on the observed rates that depend on production cross sections and branching ratios. Within this approach, shapes and distributions are unchanged with respect to the SM and are used to select signal vs background only. It is important to stress that this methodology allows to test the SM hypothesis, but not to interpret possible deviations. The experimental efficiencies are assumed to be the same as those of Run I at the LHC. The evolution of the theoretical uncertainties is treated differently by ATLAS and CMS. For ATLAS the current uncertainties where either included or not, while CMS considered two scenarios, one with the current ones and one with the theoretical uncertainties reduced by a factor of two.

The results, summarised in Tables 5 and 6, taken from [127], show that an expected relative precisions better than $10\,\%$ may be achieved within the next run and possibly improved by a factor two in the HL-LHC.

**Table 6:** Precision of Higgs boson couplings as expected by CMS and ATLAS with 300 and 3000 fb$^{-1}$ integrated luminosity at the LHC. The main assumption in the fit is that $\kappa _{{\mathrm{u}}{}{}}\equiv \kappa _{{\mathrm{t}}{}{}}=\kappa _{{\mathrm{c}}{}{}}$, $\kappa _{{\mathrm{d}}{}{}}\equiv \kappa _{{\mathrm{b}}{}{}}=\kappa _{{\mathrm{s}}{}{}}$ and $\kappa _\ell \equiv \kappa _\tau =\kappa _\mu$. The range represents spread from two different assumptions of systematic uncertainties. Table taken from Ref. [127]

Luminosity	300 fb$^{-1}$ (%)	3000 fb$^{-1}$ (%)
Coupling parameter	7-parameter fit	
$\upkappa _{{\upgamma }{}{}}$	5–7	2–5
$\upkappa _{{\mathrm{g}}{}{}}$	6–8	3–5
$\upkappa _{{\mathrm{W}}{}{}}$	4–6	2–5
$\upkappa _{{\mathrm{Z}}{}{}}$	4–6	2–4
$\upkappa _{{\mathrm{u}}{}{}}$	14–15	7–10
$\upkappa _{{\mathrm{d}}{}{}}$	10–13	4–7
$\upkappa _\ell$	6–8	2–5
$\Gamma _{{\mathrm{H}}{}{}}$	12–15	5–8
$\upkappa _{{\mathrm{Z}}{}{}{\upgamma }{}{}}$	41–41	10–12
$\upkappa _{\upmu }{}{}$	23–23	8–8
BR$_\mathrm{BSM}$	$<$14–18	$<$7–11

A summary of the final conclusions of the Snowmass study that are relevant for this discussion is:

- Higgs boson phenomenology will be studied at the LHC in the next decade. Higgs couplings to fermions and vector bosons, assuming only SM decay modes, can be determined with an estimated precision of 4–15 % for 300 fb$^{-1}$ at $14~\text {TeV}$, going to 2–10 % in the high-luminosity run (3000 fb$^{-1}$).
- Full exploitation of the LHC and HL-LHC Higgs measurements will require important improvements in precision of theoretical calculations for production as well as for branching ratios in the SM.
- At an ${{\mathrm{e}}{}{}}^{+} {{\mathrm{e}}{}{}}^{-}$ collider with sufficient integrated luminosity, SM decays and a wide range of rare Higgs boson decays, including invisible or exotic final states, will be accessible in the ${\mathrm{Z}}{}{}{\mathrm{H}}{}{}$ production through the model-independent recoil mass technique.
- Performing precision determinations of Higgs boson couplings to the 1 % level will require complementary collider programs, such as Higgs factories at linear or circular ${{\mathrm{e}}{}{}}^{+} {{\mathrm{e}}{}{}}^{-}$ colliders or even a muon collider. Only a multi-prong strategy will allow to constrain many of the couplings in a model-independent way.
- The determination of the ${\mathrm{t}}{}{} {\mathrm{t}}{}{} {\mathrm{H}}{}{}$ coupling can be done at LHC and with sufficient collision energy also at ILC. At the HL-LHC a precision of 7–10 % per experiment is expected, improving to $\sim$2–3 % at ILC with luminosity upgrade.
- The Higgs self-coupling is among the most interesting couplings still to be determined and it will remain very challenging in the coming years. At the HL-LHC a $50\,\%$ measurement per experiment could be achieved, while a linear ${{\mathrm{e}}{}{}}^{+} {{\mathrm{e}}{}{}}^{-}$ collider at $1~\text {TeV}$ could reach $13\,\%$. Further improvement would need higher collision energies, with CLIC and FCC-hh possibly going below the $10\,\%$ level.
- $C\!P$-odd couplings to vector bosons (loop induced) and to fermions will be accessible already at the LHC to a few percent precision, with further improvements from VBF production and from fermions $h\rightarrow \tau \tau$ decay and $\bar{{\mathrm{t}}{}{}}{}{} {\mathrm{t}}{}{} {\mathrm{H}}{}{}$ production.
- HL-LHC provides unique capabilities to measure rare statistically-limited SM decay modes such as ${{\upmu }{}{} ^{+}} {{\upmu }{}{} ^{-}}$, ${\upgamma }{}{} {\upgamma }{}{}$, and ${\mathrm{Z}}{}{}{\upgamma }{}{}$ and make the first measurements of the Higgs self-coupling.

Several comments are in order. First, as mentioned above, the Snowmass study is limited to the strength of the couplings and it is therefore suitable only for exploring the operators of Eq. (5). Second, results have been obtained by extrapolating measurements being performed at the time of writing. Several other opportunities for determining coupling strengths will open up with higher-energy and luminosity runs, such as constraining the Higgs-charm couplings through exclusive ${\mathrm{H}}{}{}\rightarrow J/\psi {\upgamma }{}{}$ decays [11], searching for exotic Higgs decays [171] and improving measurements of processes where the Higgs contributes off its mass shell, such as ${\mathrm{g}}{}{} {\mathrm{g}}{}{} \rightarrow {\mathrm{Z}}{}{}{\mathrm{Z}}{}{}, {\mathrm{W}}{}{}{\mathrm{W}}{}{}$ [6].

### Towards precision EFT: the road ahead

Measuring Higgs coupling strengths can be thought as the deployment of an exploration strategy. If no large discrepancies are found, such as the case now, the next logical step is to employ a model independent strategy and set limits on the new physics scale $\Lambda$ through precise determinations of the Wilson coefficients of the dim $=$ 6 SM Lagrangian. Several are the challenges, both experimental and theoretical, that such a program faces in the short- and mid-term horizon. In the following a few among the most important issues are presented, mostly related to the theoretical needs.

- *Constraints from the UV* From a UV perspective, one of the most important features of the EFT approach is its model independence: any new physics theory at high scale will generate interactions that can be fully parametrized at a given accuracy in $E/\Lambda$ by the corresponding set of operators. Given a UV theory or a general class thereof, however, one can propagate information down to lower scales and predict operator coefficients a priori. In this case RGE’s of the operators should be considered to correctly match the full theory to the effective one. Such information is now fully available at one-loop for the dim $=$ 6 SM Lagrangian [155, 172–176]. Still, it is possible to conceive large classes of UV models and imagine what structure these models could imprint in the coefficients of the EFT. We have already mentioned the power-counting in terms of couplings $g_*$, that provides a hint of what kind of effects could be enhanced, e.g., in strongly coupled theories  [25, 151]. Another example: if the underlying theory is a renormalizable gauge theory, it is possible to classify dimension-six operators as being potentially generated at tree level or at one loop [151, 177, 178]. Both the basis of Refs. [151, 152] allow this classification, while the basis of Ref. [147] does not and therefore does not offer a transparent framework to describe this type of UV models. An EFT can be thought as building a bridge to consistently collect information about deviations of the SM in order to constrain possible new physics theories lying at higher scales. Until indications become clear and if enough experimental information is available, one should keep an “agnostic” standpoint and fit the data with dimension-six operators regardless any UV-inspired classification. On the other hand, having general arguments on where to expect deviations for given classes or specific UV theories and explicit maps UV-EFT predicting the values of the most relevant Wilson coefficients will be extremely useful and necessary to characterise the information available at any given time. Furthermore, in an initial phase of the program when the limited amount of data imposes limitations to the precision and sensitivity of this types of searches, it will be important to have a hierarchical organization within the EFT description that selects some subsets of Wilson coefficients and prioritizes them.
- *Precision observables in EFT at NLO accuracy in QCD and EW* Constraining new physics via an EFT implies the ability of controlling uncertainties and therefore performing calculations at one-loop or at NLO for a wide set of observables in the EFT, including precision observables at LEP. Technical as well as conceptual challenges arise in such computations. First, the complete structure of UV operator mixing and renormalisation in both QCD and EW perturbative expansions needs to be known. In addition, suitable regularization and renormalisation schemes need to be properly defined. Another key point is that comparison with data will require the inclusion of QCD and EW corrections for the SM predictions. Understanding the pattern of higher-order QCD corrections for the dim $=$ 6 contributions will also be certainly necessary. On the other hand the inclusion of EW corrections for predictions featuring dim $=$ 6 operators might also turn out to be relevant. These issues have just began now to be explored [23].
- *The flavour structure of the dim*$=$ 6 *SM Lagrangian* The counting of the number of operators in the dim $=$ 6 SM Lagrangian strongly depends on the assumed flavoured structure. The 76 operators corresponding to one generation, become 2499 for three families. Many of these operators are four-fermion operators, that are not directly related to Higgs physics, yet can enter in precision measurements (one simple example being the muon decay width through which $G_F$ is defined). Current global studies, see e.g. [163, 165, 167] assume no special structure in flavor (flavour blindness), while Ref. [163] extends the analysis to the first order in minimal flavour violation [179] scenarios; it would be interesting to explore other patterns of flavor symmetry breaking and to include direct constraints on flavor changing neutral current (FCNC) operators.
- *Precise predictions for production and decay processes: dim*$=$ 4 *SM Lagrangian* Searching for deviations from the SM predictions can only be done if sufficiently accurate and precise predictions from the dim $=$ 4 SM Lagrangian can be obtained for the relevant observables, both for signal as well as for backgrounds. The state of the art of the predictions for SM processes in Higgs physics is often summarised as follows: NNLO in QCD and NLO in EW are known for total cross sections as well for distributions of all main Higgs production channels (barring $\bar{{\mathrm{t}}{}{}}{}{} {\mathrm{t}}{}{} {\mathrm{H}}{}{}$ which is known only at NLO in QCD). There are, however, important exceptions and notable improvements that will be needed in order to bring the precision EFT program to a success and that will keep the theoretical community busy during the LHC Run II and possibly beyond. The first important set of improvements concerns Higgs production in gluon fusion. A significant reduction of the theoretical uncertainties for the total rates is expected from the computation of the full N3LO QCD corrections on one side and from better PDF determination on the other. This latter point will drastically rely on our ability to perform an accurate an precise measurement of the gluon PDF using LHC data using NNLO predictions and the corresponding data on inclusive jet and top pair production. A full NNLO computation of the *H*+jet rates, and improvement, through resummation, of the exclusive jet rates is also expected in the coming years. Both these improvements are required already at the level of the precision on the Higgs boson signal strengths only. For the EFT program, information on the effective Higgs-gluon couplings can be gained via measurements at high $p_T(H)$, a region where top-mass effects from the loops are important and currently known only at LO. This is just one example of a rather important class of computations at NLO that will be needed, i.e., those involving loop induced processes at the Born level. Other notable examples belonging to this class are the ${\mathrm{g}}{}{} {\mathrm{g}}{}{} \rightarrow BB$ processes, with $BB={\mathrm{H}}{}{}{\mathrm{H}}{}{}, {\mathrm{Z}}{}{}{\mathrm{H}}{}{}, {\mathrm{Z}}{}{}{\mathrm{Z}}{}{}, {\mathrm{W}}{}{}^+{\mathrm{W}}{}{}^-$ which are presently known only at 1-loop level (Born). ${\mathrm{g}}{}{} {\mathrm{g}}{}{} \rightarrow {\mathrm{H}}{}{}{\mathrm{H}}{}{}$ represents the dominant Higgs pair production channel which can provide information first on the trilinear Higgs coupling, but also on the top-Higgs interactions. As already mentioned, ${\mathrm{g}}{}{} {\mathrm{g}}{}{} \rightarrow {\mathrm{Z}}{}{}{\mathrm{Z}}{}{}, {\mathrm{W}}{}{}^+{\mathrm{W}}{}{}^-$ featuring an off-shell Higgs boson, can provide complementary information and are sensitive to a wide range of new physics contributions coming from higher dimensional operators. The computation at NLO in QCD for such processes, relies on the knowledge of two-loop box amplitudes which are at the edge of the current loop technologies. A process-independent technology to obtain predictions at NNLO accuracy in QCD for final states featuring jets will also be needed for VBF. In all cases, fully differential predictions including EW effects matched to a parton shower at NLO accuracy will also be needed for all main production channels.
- *Precise and exclusive predictions for production and decay processes:dim*$=$ 6 *SM Lagrangian* Assessing deviation from the SM only needs precise predictions from the dim $=$ 4 SM itself. Interpreting them, however, needs accurate and precise predictions in the context of an EFT. Inclusion of NLO in QCD for all processes and operators of interest in Higgs physics is certainly one of the main goals of this research program. Given the large number of operators and processes to cover, only an automatic approach will be able to deliver the predictions needed. Progress in this direction has started in the context of the Higgs [15, 180, 181] and top-quark EFT. It is also important to remember that attaining NLO in QCD accuracy is mandatory for processes for which no SM mechanism is present (or is highly suppressed). A glaring example are FCN interactions involving the Higgs and quarks for which new physics appear as squared amplitudes (and not in the interference as usually is the case). All NLO predictions should be matched to a PS and available as event generators. Finally, it has to be foreseen that, were deviations found, even NNLO in QCD and EW corrections for some key observables could be needed.
- *Global approach to the determination of the Wilson coefficients* As already discussed above, UV priors should not be used to constrain operators in an independent way (unless not enough data is available). This entails that all coefficients should be directly and only constrained by data via a global fit. Identifying the optimal set of key observables, their correlations in the measurements and the mapping of each observable to a given set of operators will be part of an important and non-trivial joint theory-experimental activity. Constraints will not only come from Higgs measurements proper, but also from processes and final states that at first sight might not have any evident direct relation with Higgs physics. Well-known examples are ${\mathrm{V}}{}{}{\mathrm{V}}{}{}$ production cross sections, with ${\mathrm{V}}{}{}={\mathrm{Z}}{}{}, {\mathrm{W}}{}{}^\pm$ that test trilinear gauge couplings or top pair production cross section that constrains operators such as the chromo-magnetic/electric ones. This effort will need a coordinated action inside the groups interested in different final states/physics inside the experimental collaborations at the LHC, ATLAS and CMS, and outside with suitable working groups (such as the LHCEWWG and LEPEWWG) and theorists.
- *Advanced analysis techniques and boosted objects* Among the foreseen experimental developments, the design, test, and deployment of advanced analysis techniques will be certainly one of the directions to invest in order to maximise the information that can be obtained from data. The contribution of new physics as parametrised by the EFT never shows up as bumps (no resonances are present) but mostly as changes in the distributions and typically as enhancements in the tails. Identifying such behaviours, and quantifying them by connecting to a suitable set of operators, will need the development of dedicated tools and analyses. In this very same context, the tagging of boosted heavy objects, such as vector bosons, the top quark, and also the Higgs itself, is expected to enhance the sensitivity to dim $=$ 6 operators considerably and will play an important role.
- *Beyond EFT: the connection with dark matter (DM) searches* Evidence of physics beyond the SM comes from cosmological and astrophysical observations pointing to the existence of a form of matter, neutral and very weakly interacting. Current searches pose rather loose lower limits on its mass and simple estimates suggest a scale of the order of the EW interactions. Such states, could therefore have a mass of the order of the Higgs mass or even lighter, and more interestingly could couple to the Higgs field by the so called Higgs-portal, i.e., the $[\Phi ^\dagger \Phi ]$ term. The existence of such a state close to the Higgs mass, would therefore invalidate the straightforward use of the EFT approach outlined above. On the other hand, generalisations to the case where only the DM candidate would lie at low scales, while possible mediators and new states would all be heavier could be rather easily treated in the same framework, even though not in a completely model-independent way, as the operators would depend on the properties of the DM candidate, such as its spin and gauge representations.

**Fig. 2:**
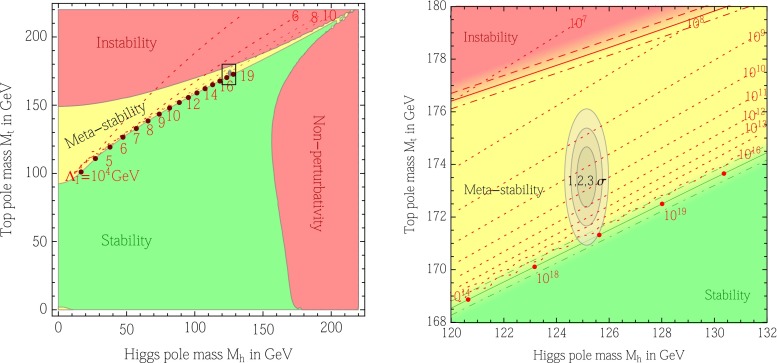
*Left* SM phase diagram in terms of Higgs and top pole masses. The plane is divided into regions of absolute stability, meta-stability, instability of the SM vacuum, and non-perturbativity of the Higgs quartic coupling. The *dotted contour-lines* show the instability scale $\Lambda _I$ in GeV assuming $\alpha _{\mathrm{s}}({M_\mathrm{Z}})=0.1184$. *Right* zoom in the region of the preferred experimental range of ${M_{\mathrm{H}}}$ and ${M_{\mathrm{t}}}$ (the *grey areas* denote the allowed region at 1, 2, and 3$\sigma$). *Plots* taken from Ref. [29]

## The Higgs potential and the electroweak vacuum

The first run of the LHC has delivered two important messages: (i) no signal of physics beyond the SM (BSM) was discovered. (ii) The Higgs boson was found exactly in the mass range 110–160 $~\text {GeV}$ predicted by the SM, using the information from precision physics and that from the direct searches at LEP and Tevatron before the turning on of the LHC.

The fact that all the mass parameters of the SM have now been experimentally determined constrains tightly the model and possibly BSM physics. New Physics (NP), if it exists, should have a marginal effect on the SM electroweak fit in order not to spoil its very good agreement with the experimental results. This fact, together with the negative result of the Run I of the LHC, indicates that BSM physics is likely to be at a high scale, possibly out of the reach of direct LHC searches.

The study of the stability of the SM Higgs potential, or if the electroweak (EW) minimum we live in is the true minimum of the SM effective potential $V^{\mathrm {eff}}$, is a general argument that can give us an indication of where the scale of NP is, or if instead the validity of the SM can be extended up to the Planck scale, $M_{ \mathrm Pl}$.

Below $M_{ \mathrm Pl}$, the appearance in $V^{\mathrm {eff}}$ of a second minimum deeper than the EW minimum, or the fact that $V^{\mathrm {eff}}$ at high scale is not bounded from below, are signals of the need (with a caveat to be discussed below) of NP to rescue the stability of the EW vacuum.

We are interested in establishing if the EW vacuum is unstable and if NP is needed, more than in pinning down the exact value of the instability scale, $\Lambda _I$, where $V^{\mathrm {eff}}$ becomes smaller than its value at the EW minimum. Therefore, it is sufficient to study the Renormalization Group (RG) evolution of the Higgs quartic coupling, $\lambda (\mu )$, with the scale $\mu$. If $\lambda (\mu )$ does not become negative up to $M_{ \mathrm Pl}$ the stability of $V^{\mathrm {eff}}$ is established.

Actually, $\lambda$ is the only SM coupling that is allowed to change sign during its RG evolution because it is not multiplicatively renormalized. Its $\beta$ function, $\beta _\lambda$, contains two competing terms, one proportional to $\lambda$ itself, i.e. the Higgs mass ${M_{\mathrm{H}}}$, and the other proportional to the fourth power of the top Yukawa coupling $y_{{\mathrm{t}}{}{}}$, i.e. the top mass ${M_{\mathrm{t}}}$, which drive the evolution of $\lambda$ towards different directions. For the present central values of ${M_{\mathrm{t}}}$ and ${M_{\mathrm{H}}}$, (and the strong coupling $\alpha _{\mathrm{s}}$ which affects $\beta _\lambda$ through its effect on the running of $y_{{\mathrm{t}}{}{}}$) a state-of-the-art computation, based on three-loop beta functions for the evolution of the couplings [182–186] and two-loop matching conditions [28, 29, 187] for the extraction of the couplings at the weak scale from the related experimental quantities, shows that the term proportional to ${M_{\mathrm{t}}}^4$ wins, driving the evolution of $\lambda$ towards smaller values and eventually going below zero at a scale of about $10^{10}~\text {GeV}$. A more refined analysis [29] shows that $\Lambda _I \sim 10^{11}~\text {GeV}$ implying that our EW minimum is not the true minimum of the Higgs potential and that there is a tunnelling probability between the EW false vacuum and the true vacuum at high field values. In this situation, we can be sure that NP must appear below $\Lambda _I$ to cure the instability of the SM potential only if the lifetime of EW vacuum is shorter than the observed age of the universe.

The rate of quantum tunnelling out of the EW vacuum is given by the probability $d\wp /dV\, dt$ of nucleating a bubble of true vacuum within a space volume *dV* and time interval *dt*. The total probability $\wp$ for vacuum decay to have occurred during the history of the universe can be computed by integrating $d\wp /dV\, dt$ over the space-time volume of our past light-cone, or

14 $$\begin{aligned} \wp \sim \tau _U^4 \Lambda _B^4\, e^{-S(\Lambda _B)}\quad S(\Lambda _B)=\frac{8\pi ^2}{3|\lambda (\Lambda _B)|}, \end{aligned}$$

where $$\tau _U$$ is the age of the universe and $$S(\Lambda _B)$$ is the action of the bounce of size $$R=\Lambda _B^{-1}$$. $$\Lambda _B$$ is determined as the scale at which $$\Lambda _B^4 e^{-S(\Lambda _B)}$$ is maximized. In practice this roughly amounts to minimizing $$\lambda (\Lambda _B)$$, which corresponds to the condition $$\beta _\lambda (\Lambda _B)=0$$ which is fulfilled for $$\Lambda _B \sim M_{ \mathrm Pl}$$. By numerical inspection of $$\wp $$ in Eq. (14) one finds that the exponential suppression wins over the large 4-volume factor if $$|\lambda (\Lambda _B)|$$ is less than $$\sim $$$$ 0.05$$.

The fact that in Eq. (14) the probability for the vacuum to decay is connected to the scale $\Lambda _B$ close to $M_{ \mathrm Pl}$ and not to $\Lambda _I$ is a signal that Planck-scale physics could affect the tunneling rate [188]. It is conceivable that at scales close to $M_{ \mathrm Pl}$ the effective potential could be sensitive to Planck-scale physics which could dramatically modify the tunneling rate. An explicit toy example of this possibility has been constructed [188]. However, we do not know anything about Planck-scale physics and therefore no conclusion can be drawn on whether the tunneling rate is modified by Planck-scale effects or not.

For the sake of our argument of looking for an unambiguous motivation for NP, possible Planck-scale effects are not relevant. Thus we discuss the lifetime of the EW vacuum assuming that unknown Planck-scale physics does not modify $\wp$ in Eq. (14). At the Planck scale one finds for $\lambda$ [29]

15 $$\begin{aligned} \lambda (M_{\mathrm{Pl}} )= & {} -0.0143 + 0.0029\left( \frac{{M_{\mathrm{H}}}}{~\text {GeV}}-125.15 \right) \nonumber \\&-0.0066 \left( \frac{{M_{\mathrm{t}}}}{~\text {GeV}}-173.34 \right) \nonumber \\&+0.0018 \left( \frac{ \alpha _{\mathrm{s}}({M_\mathrm{Z}}) -0.1184}{0.0007} \right) \end{aligned}$$

that implies that our vacuum is metastable, i.e. $\wp$ is extremely small (less than $10^{-100}$) or the lifetime of the EW vacuum is extremely long, much larger than $\tau _U$. We must then conclude that the instability of the SM Higgs potential cannot be taken as a motivation for NP. However, as shown in Fig. 2, we would have reached a different conclusion if ${M_{\mathrm{H}}}$ had been smaller, leading to a stronger instability of the Higgs potential (the red region in Fig. 2). Obviously, the vacuum stability analysis does not exclude BSM physics, which might have no impact on stability, make it worse, or ameliorate it. Examples of all the three possibilities can be easily found [189, 190].

The regions of stability, metastability, and instability of the EW vacuum are shown in Fig. 2 both for a broad range of ${M_{\mathrm{H}}}$ and ${M_{\mathrm{t}}}$, and after zooming into the region corresponding to the measured values [81–83]. The latter appear to be rather special, in the sense that the present central values of ${M_{\mathrm{H}}}$ and ${M_{\mathrm{t}}}$ place the SM vacuum in a near-critical condition at the border between stability and metastability. The NNLO computation of the stability bound , i.e. of the ${M_{\mathrm{H}}}$ value that ensures a stable potential up to $M_{ \mathrm Pl}$ (green region in Fig. 2), gives [29]

16 $$\begin{aligned} M_{{\mathrm{H}}{}{}}> & {} 129.6\, ~\text {GeV}+ 2.0 ( M_{{\mathrm{t}}{}{}} - 173.34\, ~\text {GeV}) \nonumber \\&-0.5 ~\text {GeV}\frac{ \alpha _{\mathrm{s}}(M_{{\mathrm{Z}}{}{}}) -0.1184}{0.0007} \pm 0.3{\mathrm {th}}\, ~\text {GeV}. \end{aligned}$$

Figure 2 and Eq. (16) show that to achieve an EW vacuum stable up to $$M_{ \mathrm Pl}$$ the value of top mass, identified as the pole mass, should be $$\sim $$$$ 2$$ GeV lower than the present experimental central value, $${M_{\mathrm{t}}}= 173.34$$ GeV. In terms of $${M_{\mathrm{t}}}$$ the stability bound reads

17 $$\begin{aligned} {M_{\mathrm{t}}}< (171.53\pm 0.15\pm 0.23_{\alpha _{\mathrm{s}}} \pm 0.15_{{M_{\mathrm{H}}}})\, ~\text {GeV}. \end{aligned}$$

The $\pm 0.3{\mathrm{th}}$ theoretical uncertainty in Eq. (16) is an estimate of the unknown higher order corrections. It indicates that the factor that can discriminate between a stable and a metastable EW vacuum is the exact value of the top mass, rather than a further refined computation. Figure 2, as well as the bound (17), are obtained using as renormalized mass for the top quark the pole mass, ${M_{\mathrm{t}}}^{\mathrm {pole}}$, and identifying it with the average of the Tevatron, CMS and ATLAS measurements, ${M_{\mathrm{t}}}=173.34 \pm 0.76~\text {GeV}$. This identification can be debated in two aspects. (i) From a theoretical point of view the concept of pole mass for a quark is not well defined as quarks are not free asymptotic states. Furthermore the quark pole mass is plagued with an intrinsic non-perturbative ambiguity of the order of $\Lambda _{QCD}$ due to the so-called infrared (IR) renormalon effects. (ii) The top mass extracted by the experiments, called Monte Carlo (MC) mass ${M_{\mathrm{t}}}^{MC}$, is a parameter of a MC generator determined via the comparison between the kinematical reconstruction of the top quark decay products and the MC simulations of the corresponding event. ${M_{\mathrm{t}}}^{MC}$ is sensitive to the on-shell region of the top quark but it cannot be directly identified with ${M_{\mathrm{t}}}^{\mathrm {pole}}$. The uncertianty quoted on ${M_{\mathrm{t}}}$ by the experimental collaborations refer to ${M_{\mathrm{t}}}^{MC}$ and not to ${M_{\mathrm{t}}}^{\mathrm {pole}}$. We do not know the exact relation between ${M_{\mathrm{t}}}^{MC}$ and ${M_{\mathrm{t}}}^{\mathrm {pole}}$. However, an “educated guess” is to assume that ${M_{\mathrm{t}}}^{MC}$ can be interpreted as ${M_{\mathrm{t}}}^{\mathrm {pole}}$ within the ambiguity intrinsic in the definition of ${M_{\mathrm{t}}}^{\mathrm {pole}}$, thus at the level of $\sim$250–500 GeV.

Alternative ways to get the top pole mass from the experimental determinations can be considered. The MC mass can be better related to a theoretically well defined short-distance mass, i.e. a mass defined in a renormalization scheme that avoids spurious higher-order renormalon effects, taken at a low scale of the order of the top width. The uncertainty in the translation between $${M_{\mathrm{t}}}^{MC}$$ and the short-distance mass is estimated to be $$\sim $$$$ 1~\text {GeV}$$. The short-distance mass can be then converted to the pole mass using the known relation up to $$\mathcal{O} (\alpha _{\mathrm{s}}^3)$$ with the conversion inducing a shift $$\sim $$$$ 600~\text {MeV}$$ [191].

A further possibility is to extract a short-distance mass defined in the $\overline{\text{ MS }}$ scheme, ${M_{\mathrm{t}}}^{\overline{\text{ ms }}}$, directly from the total production cross section for top quark pairs $\sigma (t \bar{t} + X)$. A recent analysis reports ${M_{\mathrm{t}}}^{\overline{\text{ ms }}}({M_{\mathrm{t}}}) = 162.3 \pm 2.3~\text {GeV}$ [191], a value that translated in terms of ${M_{\mathrm{t}}}^{\mathrm {pole}}$

18 $$\begin{aligned} {M_{\mathrm{t}}}^{\overline{\text{ ms }}}({M_{\mathrm{t}}})= & {} 162.3 \pm 2.3 ~~~\text {GeV}\rightarrow {M_{\mathrm{t}}}^{\mathrm {pole}} \nonumber \\= & {} 171.2 \pm 2.4 ~~~\text {GeV}\end{aligned}$$

gives a central value compatible with the full stability of the Higgs potential.

As already discussed, the possibility of the full stability of the SM Higgs potential requires an ${M_{\mathrm{t}}}^{\mathrm {pole}} \sim 171$ GeV. The top pole mass is the same object that enters the EW fit and it can be predicted now that we know the Higgs mass quite accurately. A recent indirect determination of ${M_{\mathrm{t}}}^{\mathrm {pole}}$ from a global fit to EW precision data, i.e. without using in the fit the experimental information on ${M_{\mathrm{t}}}$, reports ${M_{\mathrm{t}}}^{\mathrm {pole}} = 176.6 \pm 2.5~\text {GeV}$ [192]. This number shows the tendency of the EW fit to prefer high values of ${M_{\mathrm{t}}}$, therefore not supporting the possibility of an EW vacuum stable up to $M_{ \mathrm Pl}$.

It is clear that the issue of the (meta)stability of the SM Higgs potential, with its important implications for the case of NP or cosmology (like the possibility of vacuum decay during inflation), will not be fully clarified until two conditions are realized: more precise measurements of ${M_{\mathrm{t}}}$ and a better control of the uncertainty in the relation between the experimentally determined quantities and the corresponding theoretical parameters.

## Jet physics

Measurements of QCD processes are necessary to better control them in their role as backgrounds to almost all the possible channels for discovering new physics at LHC, and in order to refine our understanding of the strong sector. In the challenging task to understand the mechanism of electroweak symmetry breaking (EWSB) and to explore the TeV scales, the energy and intensity of collisions have grown in the past and will be further increased in future hadron colliders. This makes events at colliders a very busy hadronic environment.

The best reason for new physics to live anywhere near the weak scale is that it is partially responsible for the generation of this scale. New physics that is related to EWSB will naturally couple most strongly to those particles in the SM which feel EWSB most strongly, in particular the top quark and the electroweak (EW) bosons (${\mathrm{H}}{}{}$, ${\mathrm{W}}{}{}$, and ${\mathrm{Z}}{}{}$), and thus will decay preferentially into these heavy particles rather than into light quarks and leptons, which yield simpler final states. Moreover, we have compelling reasons to believe that the new particles or resonances will naturally decay to boosted SM particles [193]. Even before the LHC turned on, the lack of deviations from SM predictions for flavor or precision electroweak observables already hinted that the most-likely scale for new physics was not the vacuum expectation value $v_\mathrm{EW}$, as naturalness might have suggested, but rather $\Lambda \gtrsim$ few TeV. Evidence for this “little hierarchy” problem has of course only gotten stronger as the LHC has directly explored physics at the TeV scales. Thus many models which address the stabilization of the EW scale will naturally give rise to final states rich in boosted tops, Higgses, ${\mathrm{W}}{}{}$’s and ${\mathrm{Z}}{}{}$’s. These particles will have an appreciable cross section to be produced in a kinematic regime where they are boosted and give rise to collimated decay products. The simple picture that one hard parton corresponds to one jet breaks down badly in this scenario, and new tools are needed to separate out collimated decays from standard QCD showers.

Even in the absence of a resonance or other mechanism to preferentially populate boosted regions of phase space, looking for boosted signals can be useful for improving the signal over background ratio. In fact, any change in the reconstruction method affects both the signal and the backgrounds. Background reduction comes in two forms:

1. In high-multiplicity final states, combinatorial background is often prohibitive. When some or all of the final-state particles are boosted, the combinatorial background is greatly reduced.
2. In addition to this, it is also possible to use boosted selection techniques to identify regions where the background from other physics processes is intrinsically reduced.

We will illustrate these features using ${\mathrm {t}}\bar{{\mathrm{t}}{}{}}$ and ${\mathrm{H}}{}{}{\mathrm{V}}{}{}$ production as example.

### ${\mathrm {t}}\bar{{\mathrm{t}}{}{}}$ production

To appreciate the need for new reconstruction techniques, consider the production of a ${\mathrm {t}}\bar{{\mathrm{t}}{}{}}$ pair at fixed center-of-mass energy. If we set the jet radius $R_0$ to 0.6, a typical value used in jet reconstruction at the LHC, it is interesting to investigate the fraction of top quarks that have all three, only two, or none of their decay products (${\mathrm{b}}{}{}jj$) isolated from the others at that scale. This gives a rough estimate of how well a jet algorithm with $R=R_0$ will be able to reconstruct the three partonic top decay products as separate jets. For a centre-of-mass energy of 1.5 TeV, 20 % of the top quarks are reconstructed as three separate jets, while 20 % appear as a single jet. But at 2 TeV, only 10 % of the top quarks are reconstructed as 3 separate jets, while 45 % appear as a single jet [194]. Clearly, tops produced in the very interesting high TeV regime (>10 TeV) straddle the borderlines between several different topologies. For this reason it would be more desirable to have a flexible reconstruction method that can handle semi-collimated tops in a unified way.

### Boosted Higgs boson production

Searching for the Higgs boson in its decay to $${\mathrm{b}}{}{}\bar{{\mathrm{b}}{}{}}$$ is very difficult at the LHC, due to overwhelming QCD backgrounds. Even in associated production with a vector boson, $${\mathrm{p}}{}{} {\mathrm{p}}{}{} \rightarrow {\mathrm{H}}{}{}{\mathrm{Z}}{}{}$$ or $${\mathrm{p}}{}{} {\mathrm{p}}{}{} \rightarrow {\mathrm{H}}{}{}{\mathrm{W}}{}{}$$, the background processes $${\mathrm{Z}}{}{}{\mathrm{b}}{}{}\bar{{\mathrm{b}}{}{}}$$, $${\mathrm{W}}{}{}{\mathrm{b}}{}{}\bar{{\mathrm{b}}{}{}}$$, and even $${\mathrm {t}}\bar{{\mathrm{t}}{}{}}$$ are overwhelming. Nonetheless, thanks to Ref. [195], $${\mathrm{p}}{}{} {\mathrm{p}}{}{} \rightarrow {\mathrm{H}}{}{}{\mathrm{V}}{}{}$$, $${\mathrm{H}}{}{}\rightarrow {\mathrm{b}}{}{}\bar{{\mathrm{b}}{}{}}$$ is now an active search channel at the LHC. If we consider, for example, $${\mathrm{H}}{}{}{\mathrm{Z}}{}{}$$ production, with leptonic decay of the $${\mathrm{Z}}{}{}$$ boson, the traditional approach was to look for final states with two leptons, compatible with the $${\mathrm{Z}}{}{}$$ boson decay, and 2 $${\mathrm{b}}{}{}$$-tagged jets, and to reconstruct the invariant mass of the $${\mathrm{b}}{}{}$$-jets, and look for a peak in the distribution of $$m_{{\mathrm{b}}{}{}\bar{{\mathrm{b}}{}{}}}$$. The new approach, suggested by recent developments in jet physics, is instead to focus on events where the Higgs boson is produced with high transverse momentum, i.e. the event is characterized by $$p_{\mathrm TV}$$ > 200 GeV and cluster these events with a large jet radius ($$R= 1.2$$), such that all of the Higgs decay products are swept up in a single fat jet. The signal is now a leptonic $${\mathrm{Z}}{}{}$$$$+$$ a fat “Higgs-like” jet, and the background to this signal is now $${\mathrm{Z}}{}{}$$ plus one fat jet rather than $${\mathrm{Z}}{}{}{\mathrm{b}}{}{}\bar{{\mathrm{b}}{}{}}$$. For an unboosted search, the ultimate discriminator between signal and background is the $${\mathrm{b}}{}{}\bar{{\mathrm{b}}{}{}}$$ invariant mass: the goal is to find a resonance, a bump, in the $${\mathrm{b}}{}{}\bar{{\mathrm{b}}{}{}}$$ mass spectrum. In the boosted regime, the Higgs boson is collected into a single fat jet and the Higgs boson mass should be reflected in the invariant mass of the fat jet itself. The jet-substructure algorithms offer enough quantitative precision to discriminate between a jet from Higgs boson decay and a QCD jet. In fact, a Higgs boson that decays perturbatively into a $${\mathrm{b}}{}{}\bar{{\mathrm{b}}{}{}}$$ pair tends to generate two quarks that share in a more symmetric way the initial energy. On the contrary, QCD splitting from shower is more asymmetric. In addition, the Higgs boson decays into two (almost) massless quarks in one step, while QCD splittings prefer to share their virtualities gradually. Procedures like the “mass-drop” and “filtering” are conceived to resolve the fat jet and distinguish QCD jets from Higgs-like ones.

In this way, the background is reduced by an extent that compensates the acceptance price demanded by the high-$p_{\mathrm {T}}$ cut.

### Grooming techniques

Inspired by these new developments, a lively research field has emerged in recent years, investigating how to best identify the characteristic substructure that appears inside single “fat” jets from electroweak scale objects (see e.g. Refs. [196–198] for a review). Many “grooming” and “tagging” algorithms have been developed and are now tested in experimental analyses (in particular see Refs. [199–202] for studies on QCD jets). An example of these of new jet techniques are trimming [203] and pruning [204, 205] algorithms.

All three grooming techniques (filtering, trimming, and pruning) increase the signal to background ratio by both improving mass resolution for signal and suppressing QCD background. For example, QCD jets, whose jet masses are generated by relatively softer and less symmetric emissions, are more likely to have their masses shifted substantially downward by jet grooming than collimated perturbatively decaying particles, thus depleting the background to high-mass searches.

### Jet shapes

Another field of investigation that has seen a rapid development and will surely benefit from future investigation concerns the study of jet shapes. A jet shape is typically a function *f* defined on a jet *J* that quantifies the properties of the jet without the (explicit) use of any jet algorithm. The approach is conceptually similar to event shapes, which allow quantitative study of QCD, without requiring specific characterization of an event in terms of jets, and indeed many jet shapes are descendants of event shapes. Again their aim is to target non-QCD-like substructures in jets from QCD ones by studying the radial distribution of particles in the jet (jet broadening, differential and integrated jet shape), the spread in radiation in the plane perpendicular to the jet (planar flow [206]), the existence of subjets (*N*-subjettiness [207]), colour structure of jets (jet pull [208], dipolarity [209]), etc. We recall in the following the definition and characteristics of a few of them.

#### Radial distribution of particles within a jet

The probability of the splitting of a parton into two other partons depends on the running coupling $\alpha _{\mathrm{s}}$ evaluated at the $k_\perp$ scale of the splitting. Jet shapes which measure the angular distribution of particles in an event are therefore measuring both the strength and the running of the strong coupling constant, and are classic probes of QCD. These jet shapes are also sensitive to the colour charge of the parent parton: since $C_{\mathrm F} < C_{\mathrm A}$, an initial gluon will radiate more, and at wider angles, than an initial quark.

- *Jet broadening* given a thrust axis $\hat{n}$, we can partition the particles into two hemispheres according to the sign of $(\vec {p}_i\cdot \hat{n} )$, where $\vec {p}_i$ is the three-momentum of the *i*-th particle. For example, for dijet-like events, this is equivalent to associating each particle to a jet. Hemisphere broadening is then defined as the momentum-weighted transverse spread of the particles
  19 $$\begin{aligned} B_H = \frac{1}{\sum _{i\in H} |\vec {p}_i|} \sum _{i\in H} |\vec {p}_i \times \hat{n} | \end{aligned}$$
  where the sum runs over all particles *i* in a hemisphere *H*.
- *Differential*$\rho (r)$ and *integrated jet shapes*$\Psi (r)$ characterize the radial distribution of radiation inside a jet. Both of these shapes are defined on an ensemble of *N* jets of radius *R*. Then for $r < R$, the integrated jet shape $\Psi (r)$ is the ensemble average of the fraction of a jet $p_{\mathrm {T}}$ which is contained within a radius *r* from the jet axis. Defining $r_i$ as the distance of a constituent *i* from the jet axis
  20 $$\begin{aligned} \Psi (r) = \frac{1}{N} \sum _{J} \sum _{i\in J} \frac{p_{\mathrm {T}}(0 < r_i <r)}{p_{\mathrm T,J}}. \end{aligned}$$
  Here the second sum runs over all constituents *i* of a jet *J*. The differential jet shape $\rho (r)$ is then given by
  21 $$\begin{aligned} \rho (r) =\frac{1}{\delta r}\, \frac{1}{N}\sum _{J} \sum _{i\in J} \frac{p_{\mathrm {T}}(r <r_i < r+\delta r)}{p_{\mathrm T, J}} . \end{aligned}$$
  These variables are often included in the suite of QCD precision measurements performed by experimental collaborations, and are useful for validating parton shower models.

**Fig. 3:**
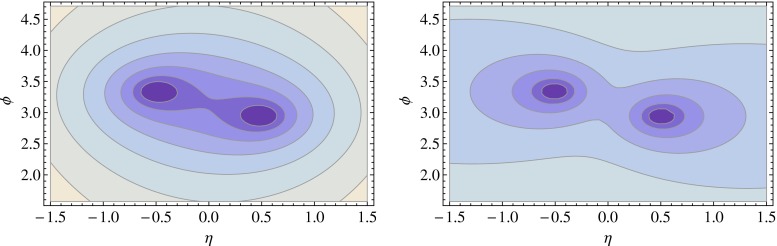
Radiation patterns in the eikonal approximation for two triplet *colour* sources *colour*-connected to each other (*left*) and to the beam (*right*). Contours are logarithmic, and the scales in the two figures are not the same. From Ref. [194]

#### Shape variables for boosted decay kinematics

The radial distribution jet shapes discussed in the previous section are geared toward probing the characteristic structure of QCD showers. Here we will recall a couple of examples of jet shapes that target evidence of non-QCD-like substructure in jets.

- *Planar flow* [206] considers the spread of the jet radiation in the plane transverse to the jet axis. Since QCD coherence gives rise to angular-ordered showers, radiation subsequent to the first emission $P\rightarrow i j$ tends to be concentrated between the clusters of energy defined by *i* and *j*, leading to a roughly linear distribution of energy in the jet. By contrast, boosted three-body decays, such as boosted tops, have a more planar distribution of energy. Planar flow is defined in terms of an auxiliary tensor
  22 $$\begin{aligned} I^{ab} = \frac{1}{m_J}\sum _{i\in J} \frac{p^a_{i,\perp } p^b_{i,\perp }}{E_i}, \end{aligned}$$
  where the indices $a,\,b$ span the plane perpendicular to the jet axis, and $\mathbf {p}_{ i,\perp }$ denotes the projection of the momentum of the *i*-th particle onto this plane. Letting $\lambda _1,\,\lambda _2$ be the eigenvalues of $I^{ab}$, the planar flow of a jet is defined by
  23 $$\begin{aligned} Pf_J=\frac{4\lambda _1\lambda _2}{ (\lambda _1+\lambda _2) ^ 2} =\frac{\det I}{(\mathrm{tr} \,I)^2}. \end{aligned}$$
  With this normalization, $Pf_J\in (0,1)$. Monte Carlo studies have demonstrated that QCD events do indeed peak at low values of *Pf*, while boosted top decays show a relatively flat distribution in *Pf*, but preliminary results show some sensitivity to shower modeling [210] and the utility of this shape in data is so far unclear. Further studies will be needed to clarify these issues.
- ${\varvec{N}}$-*subjettiness* [207] given *N* axes $\hat{n}_k$, we define *N*-subjettiness as
  24 $$\begin{aligned} \tau _N =\frac{\sum _{i\in J}p_{\mathrm T,i} \min (\Delta R_{i k} ) }{\sum _{i\in J} p_{\mathrm T,i} R_0} , \end{aligned}$$
  where $R_0$ is the jet radius, and $\Delta R_{ik}$ is the distance between particle *i* and axis $\hat{n}_k$. The smaller $\tau _N$ is, the more radiation is clustered around the chosen axes, or in other words, smaller values of $\tau _N$ indicate a better characterization of the jet *J* as having *N* (or fewer) subjets. Conversely, if $\tau _N$ is large, then a description in terms of ${>}N$ subjets is more desirable. However, as QCD alone will easily make jets with subjets, to differentiate boosted objects we need to probe not just the possible existence of subjets, but their structure. The real distinguishing power of *N*-subjettiness occurs when looking at ratios. For instance, a two-prong boosted particle such as a Higgs boson or a vector boson ${\mathrm{V}}{}{}$ will have large $\tau _1$ and small $\tau _2$. QCD jets which have small $\tau _2$ will generically have smaller $\tau _1$ than for signal, as the QCD jets are more hierarchical. Conversely, QCD jets which have large $\tau _1$ are generally diffuse, and will have larger $\tau _2$ as well than for signal. Thus the best single discriminating variable is $\tau _2/\tau _1$, or, more generally
  25 $$\begin{aligned} r_{N} = \frac{\tau _N}{\tau _{N-1}} \end{aligned}$$
  for a boosted *N*-prong particle.

#### Colour-flow variables

Beyond kinematics, boosted perturbative decays can also differ from QCD backgrounds in their colour structure. Consider a colour singlet such as a ${\mathrm{H}}{}{}$ or ${\mathrm{V}}{}{}$ boson decaying to a quark–antiquark pair. The decay quark jets form a colour dipole: they are colour-connected to each other, but not to the rest of the event. Meanwhile, the backgrounds to these processes come from QCD dijets, which necessarily have different colour connections, as shown in Fig. 3 [194], where the radiation patterns for a colour-singlet signal are plotted on the left and for a typical background on the right, as computed in the eikonal (soft) approximation. This observation has motivated work on variables which can add colour flow to the suite of variables which can discriminate signal from background.

### Conclusions

Until very recently, nearly all theoretical studies of jet substructure have been performed using Monte Carlo parton shower programs (see for instance Ref. [197] and references therein), with tools such as Herwig and Pythia. While these are powerful general purpose tools, their numerical nature masks insight into the dependence on tagger and jet algorithm parameters, which should ideally be optimised for the purposes of detecting new physics. Such a detailed level of understanding, manifested as accurate analytic QCD predictions, is a key ingredient for substructure analyses to reach their full potential. However it is far from obvious that, given their inherent complexity, jet-substructure observables can be understood to a high level of accuracy analytically.

Future progress on analytic calculations, along the lines of what has been done in Refs. [211, 212] (see for instance Ref. [193] for a review), and on the merging of high-precision fixed-order calculations with parton shower algorithms, as in POWHEG and MC@NLO, will doubtless shed more light in regards to jet substructure in the near future.

## Higher order QCD corrections and resummation

### Introduction

It is clear that precision QCD calculations are a necessary ingredient for future discoveries. The argument, which has been implicit in most of the previous discussion in this document, can be summarized as follows.

- The first run of the LHC successfully discovered a scalar boson which closely matches the properties of the Standard Model Higgs boson. This was largely expected, although by no means guaranteed. On the other hand, while operating at an energy four times higher than previously achieved, the LHC failed to uncover any signs of “new physics”: in fact, all results to date are in impressive agreement with Standard Model (SM) predictions. This is true not only for the experiments probing the high-energy end of the spectrum (roughly ‘top and Higgs’ at ATLAS and CMS), but also for intensity/precision experiments (witness the spectacular measurements [213–215] of the branching ratio of the decay $B_s \rightarrow {{\upmu }{}{} ^{+}} {{\upmu }{}{} ^{-}}$ by CMS and LHCb).
- In the next run, the LHC will extend its energy reach by a factor of roughly 1.6. Spectacular discoveries in this new energy range (typically new resonances directly produced in the *s* channel) are possible, and should indeed be hoped for. One must however be realistic: the impressive agreement of all existing data with the SM, and the relatively modest increase in the available energy, make such discoveries unlikely, in the following limited sense: we have at this point no compelling reason to expect new physics to become directly accessible between 8 and $13~\text {GeV}$.
- Such a situation is not new nor exceptional. From Kepler’s Laws to Bohr’s atomic model, disruptive physics discoveries have more often come from increased precision in the measurements of existing phenomena than from the opening of new energy ranges. It is likely that we will find ourselves, in the next several years, once again in a situation in which our best available option for discovery will be increasing the accuracy and precision of experimental measurements, and of theoretical predictions based on existing theories.
- More specifically, in collider physics language, even if we don’t have the energy to directly access new very massive states via ‘*s*-channel’ production, we can still (hopefully) measure their contributions to low-energy observables via virtual exchanges. These can be for example *t*-channel exchanges, which would induce deviations from SM predictions in the tails of energy distributions, or loop-level exchanges affecting SM parameters such as couplings or mixing angles. Such small deviations from SM predictions can only be reliably observed if the SM-based theoretical prediction is sufficiently precise and accurate. In this limited sense, it is quite possible that future discoveries in high-energy physics may hinge on the degree of accuracy that our calculations can reach.

With these general premises, one hardly needs to emphasize the relevance of advanced QCD calculations for future experiments. The LHC is a hadron collider, and any precision measurement in such a collider requires, one way or another, a detailed understanding of the underlying QCD phenomenology. Future linear (or circular) ${{\mathrm{e}}{}{}}^{+} {{\mathrm{e}}{}{}}^{-}$ colliders may focus part of their program on the production of electroweak final states (${\mathrm{Z}}{}{}, {\mathrm{Z}}{}{}{\mathrm{Z}}{}{}, {\mathrm{H}}{}{}, {\mathrm{H}}{}{}{\mathrm{H}}{}{}\dots$), however many crucial inputs and outputs from such searches will be driven by QCD (top production, jet studies, $\alpha _{\mathrm{s}}$ measurements, to name a few).

The focus of this section will be on QCD resummations: the question is then how resummations, given the context described above, can be useful for selected phenomenological applications. A related question is what kind of resummation technology we may expect to be available on the time scale of several years. We will first summarize some basic facts about the classic formalism of soft-gluon resummation, and then turn to more recent developments, by tackling the above two questions in reverse order: first tentatively outlining the likely future developments in QCD resummations, and then making a few observations on possible phenomenological applications.

### Sudakov resummation

The all-order summation of the logarithmically-enhanced perturbative corrections produced by soft-gluon radiation, also known as Sudakov resummation [216–219], is a very important topic for physics studies within (and beyond) the Standard Model, at present and future accelerator energies.

The origin of the Sudakov logarithms is well known. In particular kinematical regions, where the contributions of real and virtual parton emissions are highly unbalanced, the reliability of the standard perturbative expansion (i.e., order-by-order in powers of the QCD coupling $\alpha _{\mathrm{s}}$) is spoiled by the presence of large double-logarithmic terms, which are finite residual effects of the cancellation of infrared (soft and collinear) singularities in IR-safe QCD cross sections. The predictivity of the perturbative expansion can be restored by summing these logarithmically-enhanced contributions to all order in $\alpha _{\mathrm{s}}$.

Sudakov resummation can be performed with analytical techniques by exploiting dynamics and kinematics factorizations. While dynamical factorization is a general property of multi-gluon QCD amplitudes in the soft limit, phase-space factorization strongly depends on the observable under consideration. If the phase-space in the soft limit factorizes, multi-gluon emissions can be written in the form of a generalized exponentiation of the single-gluon emission probability. In such cases it is possible to perform an improved perturbative expansion that systematically resums, to all orders in $\alpha _{\mathrm{s}}$, the leading (LL), next-to-leading (NLL), next-to-next-to-leading (NNLL) (and so forth) logarithmic contributions.

In the following we briefly discuss Sudakov resummation in the case of two of the most important hard-scattering observables in hadronic collisions: inclusive cross sections in the threshold region and transverse-momentum ($q_{\mathrm T}$) distributions at low $q_{\mathrm T}$.

#### Threshold resummation

Threshold logarithms appear in the perturbative expansion of inclusive cross sections when the observed high mass ($M^2$) system is forced to carry a very large fraction *x* of the available (partonic) centre-of-mass energy $\sqrt{s}$. The kinematical variable $x=M^2/s$ parametrises the distance from the partonic threshold $x=1$. In the kinematical region close to the partonic threshold ($x\rightarrow 1$) the emission of real radiation at higher orders is strongly suppressed. As a result, large logarithms of the type $L = \ln (1-x)$ appear order-by-order in the perturbative expansion, in the form

26 $$\begin{aligned} c_{nm} \alpha _{\mathrm{s}}^n L^{m},\quad \text{ with }\; 1\le m\le 2n. \end{aligned}$$

In order to get reliable perturbative predictions, these logarithmic corrections (which diverge in the $x \rightarrow 1$ limit) have to be resummed to all orders [216, 217, 220]. In the case of inclusive cross sections near threshold, phase-space factorization is obtained by working in the conjugated Mellin (*N*-moment) space where soft-gluon resummation can be systematically performed (see, however, [221] for an alternative viewpoint).

Due to finite experimental acceptances, theoretical predictions in Mellin space cannot be compared directly with data, and the inversion to the physical *x*-space has to be performed. Resummed expressions, however, diverge at very large *N*, where the Landau singularity in the QCD running coupling signals the onset of non-perturbative phenomena. The Mellin inversion can be performed only after the introduction of a prescription which regularizes the Landau singularity [222–225].

The formalism to perform threshold resummation was first developed in the case of processes involving two QCD partons at the Born level [216–220] and successively extended to the more general case of inclusive cross sections in multiparton processes [226–231]. More recently threshold resummation techniques based on effective theories have been developed [232–238].

Some processes in hadronic collision where threshold resummation is particularly important are the production of vector and Higgs bosons [219, 239–247], prompt photons [229, 230, 237, 248, 249], heavy quarks [226, 227, 229, 236, 250–252], and jet and single-hadron inclusive production [222, 253–256].

#### Transverse-momentum resummation

Transverse-momentum logarithms occur in the transverse-momentum ($q_{\mathrm T}$) distribution of high invariant mass systems (*M*) in the region of small $q_{\mathrm T}$ ($q_{\mathrm T} < < M$). Also in this case the suppression of real emissions gives rise to large double-logarithms of the type $L = \ln M^2/q_{\mathrm T}^2$, order-by-order in perturbation theory.

Transverse-momentum resummation for the hadroproduction of an arbitrary system of colourless particles, first developed in the series of papers [257–265], is nowadays well understood [266–269]. Some examples of such systems are DY lepton pairs [65, 270], Higgs boson [271–273] and diboson production [274–276].

On the contrary, in the case of hadroproduction of systems that involve coloured QCD partons, the structure of colour correlations and coherence effects lead to theoretical complications which have still prevented a fully general extension of the resummation formalism. Nonetheless the phenomenological importance of multiparton scattering processes together with the high precision experimental data, strongly demand generalizations of the transverse-momentum resummation formalism for such processes.

Recent theoretical progress in this direction was obtained in Refs. [277, 278] by considering the specific case of the hadroproduction of a heavy-quark pair ($Q\bar{Q}$) with a small $q_{\mathrm T}\ll m_Q$.

Transverse-momentum resummation has also been reformulated in the framework of effective theories [279–284] and it can also be performed by using approaches beyond the customary QCD framework of collinear factorization, that use transverse-momentum dependent (TMD) factorization and introduce transverse-momentum dependent parton distributions functions (TMD PDFs) (see Ref. [286] and references therein).

#### Universality of Sudakov resummation

An important aspect of Sudakov resummation is related to its universality (i.e., process independence). Resummed cross sections can be expressed in a factorized form which involves a *process-independent form factor* which resums to all orders the corrections due to soft and collinear parton emissions, and a *process-dependent hard factor* which takes into account hard-virtual contributions. The all-order resummation of the logarithmic corrections is controlled by these factors which are expressed in terms of perturbative functions with coefficients computable order-by-order in perturbation theory.

In the case of hadroproduction of colourless particles, it has been shown that the hard factor, despite its intrinsic process dependence, has an all-order universal structure [269]. The process-dependent information encoded in the hard factor can be entirely extracted by the scattering amplitude of the Born-level partonic subprocess and its virtual radiative corrections [269]. The hard resummation factor is directly determined by a universal (process-independent) all-order factorization formula, that originates from the factorization properties of soft and collinear parton radiation, and by the knowledge of the corresponding scattering amplitude. This factorization formula has been explicitly evaluated, in the case of hadroproduction of an arbitrary system of colourless particles, up to the next-to-next-to-leading order (NNLO) in the case of $q_{\mathrm T}$-resummation [269] and N$^3$LO in the case of threshold resummation [58]. Results in the case of the production of coloured objects have been obtained in Refs. [256, 278].

#### Other aspects

Sudakov resummation techniques have nowadays reached a high level of accuracy and resummed calculations up to NNLL order are available for various observables in many different processes. This increasing precision is of fundamental importance to fully exploit the discovery potential provided by the high quality of the collected and forthcoming collider data. For instance the successful accomplishment of the LHC physics program will depend on the ability to provide precise theoretical predictions. Many experimental results are indeed sensitive to soft-gluon effects and resummed calculations (consistently matched to standard fixed-order results) allow us to enlarge the applicability of precise perturbative QCD predictions.

Let us finally stress that analytic techniques to perform all-order Sudakov resummation are also important for other aspects of perturbative QCD. The parton shower algorithms which are implemented in Monte Carlo event generators resum to all-order leading-logarithmic corrections due to collinear and soft emissions. Analytic resummation techniques can thus be used to improve parton showers beyond their present logarithmic accuracy. Another important aspect is related to fixed-order computations. This is the case, for instance, of the *subtraction formalism* of Ref. [287] which permits to perform fully-exclusive NNLO calculations using the knowledge of the transverse-momentum distributions in the small $q_{\mathrm T}$ region.

#### Conclusions

Sudakov resummation techniques have nowadays reached a high level of accuracy and resummed calculations up to NNLL order are available for various observables in many different processes. This increasing precision is of fundamental importance to fully exploit the discovery potential provided by the high quality of the collected and forthcoming accelerator data. For instance the successful accomplishment of the LHC physics program will depend on the ability to provide precise theoretical predictions. Many experimental results are indeed sensitive to soft-gluon effects and resummed calculations (consistently matched to standard fixed-order results) allow us to enlarge the applicability of precise perturbative QCD predictions.

Let us finally stress that analytic techniques to perform all-order Sudakov resummation are also important for other aspects of perturbative QCD. The parton shower algorithms which are implemented in Monte Carlo event generators resum to all-order leading-logarithmic corrections due to collinear and soft emissions. Analytic resummation techniques can thus be used to improve parton showers beyond their present logarithmic accuracy. Another important aspect is related to fixed-order computations. This is the case, for instance, of the *subtraction formalism* of Ref. [287] which permits to perform fully-exclusive NNLO calculations using the knowledge of the transverse-momentum distributions in the small $q_{\mathrm T}$ region.

### Resummations: future developments

The development of resummation technology proceeds mainly in two ways. On the one hand, there are well-established theorems (see, for example, Ref. [288]) stating that, for certain inclusive cross sections, *all* logarithms associated with soft and collinear emissions exponentiate. For such cross sections progress comes in the form of increased accuracy: new finite-order calculations provide the values for the relevant anomalous dimensions, and the contributions of more towers of logarithms become explicitly known. On the other hand, resummation theorems can be extended in various directions, for example to less inclusive or more complicated observables, or to new classes of logarithms. Let us tackle these two lines of progress in turn.

#### Towards greater logarithmic accuracy

Recent years have seen remarkable progress in high-order perturbative calculations (see for example [289]). Further progress is to be expected in the next several years, as new techniques are brought to fruition. While it is very difficult to predict developments on a time scale of 5–10 years, it is perhaps useful to attempt to list what might happen. The following is a tentative list of finite order perturbative calculations, relevant for resummations, which might be completed within the time frame we are considering.

- Fully inclusive electroweak final state process. The three-loop ($\mathrm{N}^3$LO) corrections to the inclusive cross sections for the Drell–Yan process, for W and Z production, and for Higgs production via gluon fusion, are being computed [290, 291] (time scale: 1 year^3^). The contributions at this order which are relevant for threshold resummation are already known [241, 242, 293] and being put to use by several groups [57, 58, 246, 247, 294, 295].
- It is to be expected that simple distributions for these processes ($p_{\mathrm T}$, rapidity) at the same accuracy will become known in the medium term (time scale: 3 years), since the required techniques are known and the increase in complexity is incremental.
- The fully subtracted $\mathrm{N}^2$LO cross section for two-jet production in QCD is being computed [296] (time scale: 2 years). This is only marginally useful for resummation since all relevant anomalous dimensions are known at this accuracy, but virtual corrections (which have been known for some time) provide necessary matching conditions for possible NNLL resummations.
- A fully exclusive analysis of three-jet production at NNLO may require significant refinements of current subtraction techniques (witness the time scale of the two-jet calculation). It is however likely that virtual two-loop corrections, necessary for matching conditions, will become known on a time scale of 3–5 years.
- The techniques for the calculation of DGLAP splitting functions at four loops ($\mathrm{N}^3$LO), and in fact of the complete DIS structure functions at $\mathrm{N}^4$LO are in principle available, and the calculation could be performed on a time scale of several years.
- In the meantime, techniques are becoming available to compute the anomalous dimensions relevant for resummations directly, without resorting to fitting these values from finite order calculations of specific processes. The three-loop soft anomalous dimension matrix for generic multi-parton scattering processes in QCD is being computed (time scale of 1 year for the massless case,^4^ and 2–3 years for the massive case, see for example Refs. [298–301] for a review of recent progress).

This (potential) wealth of new finite order results would almost automatically lead to a considerable refinement of existing resummation techniques. Here’s a list of what could become available within the stated timescale.

- The inclusive cross sections and simple inclusive distributions (such as $p_{\mathrm T}$ and rapidity) for electroweak annihilation processes will be available with $\mathrm{N}^3$LL accuracy, fully matched to exact $\mathrm{N}^3$LO calculations. The processes include Drell–Yan, ${\mathrm{W}}{}{}$ and ${\mathrm{Z}}{}{}$ production, Higgs production in gluon fusion, but also for example di-boson production (two ${\mathrm{Z}}{}{}$’s, two Higgses, ...) where however the matching to $\mathrm{N}^3$LO will remain incomplete for some time.
- Sufficiently inclusive jet distributions (single inclusive jet $p_{\mathrm T}$, dijet mass, ...) will be known to $\mathrm{N}^2$LO, with $\mathrm{N}^2$LL threshold resummation. This involves some subtle issues of non-universality w.r.t. jet algorithms, and the existence of non-Sudakov logarithms, which however are likely to have been tackled within our stated time frame.
- The calculation of four-loop DIS structure functions (supplemented with a value for the five-loop light-like cusp anomalous dimension) would lead to a fairly stunning resummed prediction at $\mathrm{N}^4$LL. Given the rather detailed knowledge of power-suppressed corrections near threshold in this case [302], structure functions would stand to remain the best predicted quantities in perturbative QCD for quite some time, with likely effects on the accuracy of PDF fits.
- If the need were to arise, typically if a Linear Collider or CLIC is built, much of the knowledge of space-like QCD processes will not be too difficult to transfer to time-like kinematics, and we can expect more detailed calculations of event shape distributions, resummed with $\mathrm{N}^3$LL (and perhaps at some point $\mathrm{N}^4$LL) accuracy, matched to NNLO (and perhaps in future at $\mathrm{N}^3$LO), and with detailed QCD-motivated models of power corrections.

#### Theoretical developments

The second line of development in resummations is the extension of existing techniques to new observables or new classes of logarithms. This is of course much more difficult to predict, since it involves fundamental theoretical progress. Here are some examples of what can be expected to happen.

- Studies are under way to extend threshold resummations to logarithms suppressed by a power of the threshold variable, or ‘next-to-leading-power’ (NLP) threshold logarithms, see for example Refs. [303–309]). Partial resummed formulas already exist for some inclusive cross sections and a systematic treatment is likely to be available within a few years. The phenomenological impact of these logarithms is not yet clear [291, 310–312], but experience suggests that further reductions in scale uncertainties are a likely effect.
- Anomalous dimensions required for $\mathrm{N}^2$LL resummations for multi-leg processes have been available for some time, and those needed at $\mathrm{N}^3$LL will become available within a few years. Here the issues are: the selection of appropriate observables, involving only a limited number of scales and not affected (or affected in a controlled way) by non-Sudakov logarithms, and the availability of matching conditions to preserve an adequate finite-order accuracy.
- Jet cross sections, and in general less inclusive cross sections, are affected by new classes of potentially large logarithms arising from phase space cuts and constraints. Examples are non-global logarithms [313] and clustering logarithms [314, 315]. These logarithms typically enter at NLL level in the threshold counting, they can be numerically important and contain interesting physical information. Several groups are engaged in studying the resummation of these logarithms, or the optimization of observables in order to minimize their effects (see, for example, [316]).
- In view of the complexity of typical LHC observables, and also of the flexibility required to consider many possible cross sections, a very important development in resummation techniques is going to be the extension of existing numerical codes (such as Caesar [317]) to $\mathrm{N}^2$LL accuracy [318]. This is non-trivial, since the logarithms involved are to some extent non universal, and one should ultimately include non-Sudakov logarithms as well, which are not known at this accuracy. Solving these problems would however provide a tool applicable to a vast array of processes.
- In a similar vein, an important development, which would be to a large extent numerical, is the matching/merging of the analytic resummation techniques, so far applied to highly inclusive cross sections at high accuracies, with the parton shower language, which is much more flexible but not easy to extend to higher logarithmic accuracy. Work in this direction is in progress by several groups, the difficulty to a large extent being the very different languages spoken by the two communities.

### Resummations: applications

Aside from generic claims that greater theoretical accuracy is important, and resummations are likely to help to achieve it, one should ask what specific processes/quantities are most likely to be relevant for new physics searches, and are also affected by resummations. A negative example would be the Higgs mass, which is of course very important, but can be precisely determined from very clean processes such as ${\mathrm{H}}{}{}\rightarrow {\mathrm{Z}}{}{}{\mathrm{Z}}{}{}\rightarrow 4 \mu$ where QCD corrections and extra QCD radiation are not relevant.

Let us consider briefly two situations where resummations can be important. On the one hand, the precise determination of (certain) Standard Model parameters and input data, which are of great relevance because they enter almost all theoretical predictions;^5^ on the other hand, a few classes of processes where resummed predictions are likely to make a significant impact.

#### Resummations for precision in SM parameters

- Although the strong coupling has been quoted with an uncertainty of the order of $0.7\,\%$ [191], there is evidence of unsolved theoretical problems that might lead to an upwards revision of the stated uncertainty. On the one hand, there are tensions between determinations from different process: for example, early values extracted at the LHC tend to be significantly lower than the world average [191]. On the other hand, some of the best controlled predictions, which involve event shapes at ${{\mathrm{e}}{}{}}^{+} {{\mathrm{e}}{}{}}^{-}$ colliders, give different results depending on the detailed treatment of resummation effects and power corrections [319–321]. An improved prediction for jet cross sections at the LHC, involving NNLO and NNLL contributions, as well as a treatment of non-Sudakov logarithms and an improved understanding of power corrections, is very likely to make an impact on the determination of $\alpha _{\mathrm{s}}$ in hadron-hadron collisions. Further studies of resummation and power correction effects are also probably needed to shed light on the tensions between different methods to determine $\alpha _{\mathrm{s}}$ at lepton colliders.
- Somewhat similarly, the top quark mass has an official uncertainty well below $1\,\%$ [191], which is almost certainly underestimated. In fact, when the stated uncertainty goes below $1~\text {GeV}$, it becomes inevitable to deal with the theoretical details of the definition of the mass parameter [191, 322]. The most precise determination of the top mass is likely to come ultimately from a lepton collider, and (as shown by existing studies), improvements in resummation technology have been and will be important ingredients in achieving the impressive goal of an uncertainty in the per mil range (here both threshold and Coulomb enhancements need to be addressed [236, 323]).
- Another ubiquitous ingredient for precision LHC predictions is the determination of Parton Distribution Functions (PDFs). These are currently determined by means of global fits to DIS and collider data by several collaborations, and the standard is NNLO accuracy. It was observed already some years ago [324] that the technology exists to determine PDFs with resummed NLL+NNLO accuracy, which could soon be extended to NNLL+$\mathrm{N}^3$LO. Since, for several cross sections of interest at the LHC, PDFs are now, or could become, a dominant source of uncertainty, such improvements are likely to play an important role. In some cases (for example the Higgs production cross section) the uncertainty on matrix elements is accidentally still large enough to compete with the one associated with PDFs. Matrix element uncertainties are however going to decrease with time as new calculations become available, and the general need for more precise PDFs is likely to increase. One must finally consider the fact that PDFs determined at finite orders are routinely being used in conjunction with resummed matrix elements, an inconsistency which could be physically relevant in some cases, and which can be corrected with existing techniques: indeed, first steps towards a global PDF fit including resummation effects were taken in [325], where this issue is discussed in greater detail.

#### Resummations for new physics

- Threshold resummations are playing a role in setting limits for the masses of heavy new physics states, both colorless [326–328] and colored [329–332], which could be produced at hadron colliders. The reason is simple: if the states are heavy as compared to the available center-of-mass energy, they must be produced (if at all) near threshold. In that case, typically threshold logarithms are large and enhance the production cross section. If this enhancement is known, it leads to sharper mass limits in the case of non-observation. This technique has already been applied to a selection of supersymmetric models, for the production of sleptons, gauginos, and colored SUSY partners such as gluinos and squarks. Of course, if in due course some of these states are observed, resummed calculations will help a precise determination of their quantum numbers and interactions.
- A fashionable topic of investigation is the subtle relation between the SM parameters (most notably the top mass and the Higgs mass) and the stability of the electroweak broken-symmetry vacuum, also discussed here in Sect. 5. Renormalization-group arguments suggest that current experimental values of $m_{{\mathrm{t}}{}{}}$ and $m_{{\mathrm{H}}{}{}}$ place the universe close to the edge between stability and metastability [29], and various possibilities are being explored as to why it should be so. The universality of this conclusion has recently been challenged [84, 188], with the argument that new physics effects even at the Planck scale are likely to drastically alter the scenario. These studies however remain a strong motivation for a precision determination of $m_{{\mathrm{t}}{}{}}$ and $m_{{\mathrm{H}}{}{}}$: even if the location of the stability boundary is not universal, for a given new physics model it can in principle be determined. A precise knowledge of the parameters can then be used to sharpen the limits on the new physics arising from the requirements of stability. This provides extra motivation for accurate determinations of $m_{{\mathrm{t}}{}{}}$, which, as discussed, crucially involve resummation techniques.
- A broad and quickly developing field of investigation is the study of jet shapes (see, for example, Refs. [333, 334]), with special emphasis on the detection of heavy, but also heavily boosted, objects, which are produced and then decay inside a jet cone (for a review of recent developments, see Ref. [335]). These techniques are already being used to explore Higgs and top properties in channels previously thought to be inaccessible due to large backgrounds [195]. Most of the currently available techniques are numerical, and make use of showering algorithms. Work is however starting on analytic techniques [211, 212], which involve the resummation of logarithms of scales arising from the jet substructure (for example the ratio of a jet radius to the radius of a selected subjet). Analytic control on such resummations is likely to improve our understanding also of power-suppressed corrections linked to hadronization, which are known to become large for small jet radii [336, 337]. Furthermore, this field sees parallel developments in numerical, shower-driven techniques, and resummations: it would be an interesting area for cross-fertilization between the two methods.

## Monte Carlo tools

The development of high energy physics experiments carried out at colliders with increasing centre-of-mass energy, has seen a parallel development in the tools for the calculation and simulation of hard processes. In the 1980’s, calculation of collider processes were typically performed at tree level, and full simulation of the events relied upon the leading-log (LL) shower approximation. Next-to-leading order calculations were only available for a handful of processes.

In the last 15 years, prompted by the perspective of the LHC runs, a remarkable progress has taken place in several areas. Fully automated techniques have been developed for the calculation of next-to-leading order (NLO) cross sections, by several collaborating and competing groups. Techniques for combining fixed order calculations with parton shower generators have appeared, and have been widely applied to collider processes. Intensive work on next-to-next-to-leading order (NNLO) calculations has been carried out by several groups, with several new NNLO results having appeared since a little more than a year. Methods for interfacing NNLO calculations to shower Monte Carlo generators have also appeared for relatively simple processes.

This section summarizes what is available at present, and illustrates what can be considered to be frontier research in this field. Although it is impossible to predict what will be available 10 years from now, it may be safely assumed that current frontier research will have turned into commonly used tools by that time.

### Presently available results

Parton Shower Monte Carlo generators (PS) fully simulate hadronic production processes by merging together a QCD component (the Shower itself) and a model for hadron formation. The QCD component is typically given in the collinear approximation. When applied to infrared finite observables, PS generators are accurate only in the collinear and soft regions, failing to predict hard, large angle emissions even at leading order. In Ref. [338] a procedure was developed for matching matrix element calculations with PS generators (ME+PS), such that the production of hard, widely separated jets could be improved to LO accuracy. This prompted the application of ME+PS techniques to various ME generation tools, like, for example in ALPGEN with the MLM matching procedure (for a list of available ME+PS generators see Ref. [339]).

In the past 10 years, considerable effort has gone in building NLO-improved PS generators (NLO+PS). Methods like Mc@Nlo [59] and POWHEG [340, 341] allow to interface fixed order NLO calculations to parton shower generators like PYTHIA [342, 343] and HERWIG [95, 344]. In essence, for a given process, these techniques extend the precision of the generator to NLO QCD accuracy for inclusive processes, and to tree level for the given process in association with one jet. For example, an NLO+PS generator for Higgs production (a process of order $\alpha _{\mathrm{s}}^3$ at the Born level) will yield distributions accurate up to order $\alpha _{\mathrm{s}}^4$. That amounts to NLO accuracy for inclusive quantities (i.e. quantities that do not depend upon the emission of associated jets, like the rapidity distribution of the Higgs, and already receive contributions at order $\alpha _{\mathrm{s}}^2$), and to LO accuracy for processes involving the emission of an associated jet that start at order $\alpha _{\mathrm{s}}^3$. Recently, these techniques have seen considerable progress, due to the appearance of computer frameworks that automatize some or all aspects of the calculation: the virtual contributions, the implementation of a subtraction framework for the real corrections, and the interface to a PS. In the MadGraph5_aMC@NLO framework [107], all aspects of an NLO calculation are automatized, starting from the generation of the LO and NLO matrix elements, down to the event generation interfaced to a PS program. The GoSam [345], Recola [346] and Open Loops [347] frameworks deal with the automatic generation of general-purpose virtual amplitudes. The BlackHat [348] generator provides virtual corrections for selected processes (vector boson production in association with jets) and is capable to deal with fairly high jet multiplicities. In fact it was recently used to compute *W* production with five associated jets at NLO [349]. The Sherpa generator [350] implements a framework for NLO calculations and for NLO+PS generation based upon a variant of the Mc@Nlo method. The so called MatchBox framework [351] implements NLO+PS generators within the Herwig++ [344] PS generator. The POWHEG BOX framework automatizes all aspects of the NLO calculation interfaced to a PS generator, except for the computation of the matrix elements. For these it relies upon other programs, like MadGraph, for the real matrix elements, and GOSAM for the virtual corrections.

Electroweak corrections are not presently included in any publicly available automatic NLO calculators. It is however clear that the same techniques that have been applied for automated NLO QCD can be extended to the full Standard Model, as well as to any renormalizable model. Interfacing calculations including electro-weak corrections to Shower Monte Carlo requires the ability to handle together QED and QCD collinear showers, but it does not present new conceptual problems with respect to QCD corrections alone. In fact, in few simple cases NLO calculation matched with Shower generators have appeared in the literature [74, 75].

### NNLO calculations

Next-to-next-to-leading order calculations (NNLO) for collider processes have first appeared in 1990 for the Drell–Yan process [352], followed more than 10 years later by the NNLO computation of the total Higgs cross section in gluon fusion [353–355], and of Higgs differential distributions [287, 356]. We have witnessed since then a steady increase in the complexity of the processes for which NNLO calculations have become available: 3 jet cross sections in ${{\mathrm{e}}{}{}}^{+} {{\mathrm{e}}{}{}}^{-}$ annihilation [357], ${\mathrm{W}}{}{}{\mathrm{H}}{}{}$ and ${\mathrm{Z}}{}{}{\mathrm{H}}{}{}$ production [358, 359], ${\upgamma }{}{} {\upgamma }{}{}$ production [360]. In a little more than a year from now, several new results for complex $2 \rightarrow 2$ processes have become available: Higgs production in association with a jet [361], ${\mathrm{t}}{}{} \bar{{\mathrm{t}}{}{}}{}{}$ production [102], a partial result on inclusive jets production [296], ${\mathrm{Z}}{}{}/{\mathrm{W}}{}{}+{\upgamma }{}{}$ production [362], ${\mathrm{Z}}{}{}{\mathrm{Z}}{}{}$ production [363], ${\mathrm{W}}{}{}^+{\mathrm{W}}{}{}^-$ production [364] and *t*-channel single top production [365]. Important results have also been obtained for decay processes [366–368].

There are several components that make up a NNLO calculation, besides the two loop corrections. One must also supply the square of 1-loop contribution (double virtual), the virtual correction to one real emission (real-virtual) and the two-real-emission contributions. Each contribution contains soft and collinear divergences, that must cancel in the sum. This also constitutes a challenging aspect of NNLO calculations. There are several techniques currently developed for implementing these cancellations. The $q_{\mathrm T}$ subtraction method [287] has been used for Higgs, Drell–Yan, ${\upgamma }{}{} {\upgamma }{}{}$, ${\mathrm{W}}{}{}{\mathrm{H}}{}{}$, ${\mathrm{Z}}{}{}{\mathrm{H}}{}{}$ and ${\mathrm{Z}}{}{}{\mathrm{Z}}{}{}$ production processes. It is particularly useful for processes where the final state is a colour neutral system. The Antenna subtraction method [369] has been used for the computation of ${{\mathrm{e}}{}{}}^{+} {{\mathrm{e}}{}{}}^{-} \rightarrow 3\,\mathrm{jets}$ and for dijets, and is presently also used in an effort to compute fully differential ${\mathrm{t}}{}{} \bar{{\mathrm{t}}{}{}}{}{}$ production at NNLO [370] (now including only the $q\bar{q}$ initial state). The so-called STRIPPER method (sector improved phase sPaCe for real radiation) [371, 372] has been used for ${\mathrm{t}}{}{} \bar{{\mathrm{t}}{}{}}{}{}$, ${\mathrm{H}}{}{}+j$ and *t*-channel single top production. Another method being developed is described in a series of publications (see [373] and references therein).

The computation of the double virtual contribution is very demanding. Recent progress with integrals including massive particles [374–376] have opened the possibility of computing NNLO corrections to pairs of massive vector bosons. In general, it seems that today two-loop virtual corrections to generic $2\rightarrow 2$ processes are feasible. A recent groundbreaking technique introduced by Henn [377] is among the developments that have made this possible. ${\mathrm{W}}{}{}+\,$jet is now know at NNLO [378] and there is a phenomenologically complete calculation of ${\mathrm{H}}{}{}+\,$jet through NNLO in Ref. [379].

### Current developments: NLO+PS merging and NNLO+PS generators

NLO+PS merging deals with the merging of NLO+PS generators of different associated jet multiplicity. Consider for example Higgs production in gluon fusion, a process of order $\alpha _{\mathrm{s}}^2$ at the Born level. Let us call H, HJ and HJJ the NLO+PS generators for the production of a Higgs, of a Higgs in association with a jet, and of a Higgs in association with two jets respectively. The H generator will yield $\alpha _{\mathrm{s}}^3$ accuracy; that is to say NLO accuracy for observable that are inclusive in the emission of associated jets, like the Higgs rapidity distribution, that include terms of order $\alpha _{\mathrm{s}}^2$ (LO terms) plus terms of order $\alpha _{\mathrm{s}}^3$ (NLO terms), and LO accuracy for observables requiring an associated jet, that are given at the lowest order by terms of order $\alpha _{\mathrm{s}}^3$. Observables requiring more than two associated jets will be generated by the shower Monte Carlo in the collinear approximation. The HJ generator is capable of yielding NLO accuracy (i.e., $\alpha _{\mathrm{s}}^4$ accuracy) for observables involving the Higgs plus one jet, that are inclusive in the emission of further jets, and LO accuracy for those requiring two jets. It would be however unpredictive for fully inclusive observables. A merged H-HJ generator would have, in addition, NLO (i.e. $\alpha _{\mathrm{s}}^3$) accuracy for fully inclusive observables. In general one may ask to merge even more NLO+PS generators, for example H+HJ+HJJ, in order to have NLO accuracy (i.e. $\alpha _{\mathrm{s}}^5$ accuracy) also for observables involving two associated jets, and thus LO accuracy for those involving three associated jets.

Notice that NLO+PS merging can be seen as an intermediate step in the construction of NNLO+PS generators. Thus, for example, if we have an H+HJ merged generator, we know that it is already accurate at the $\alpha _{\mathrm{s}}^4$ level for all observables, except for those that are totally inclusive in the emission of associated partons, where the accuracy is instead $\alpha _{\mathrm{s}}^3$. If we could reach $\alpha _{\mathrm{s}}^4$ accuracy for inclusive observabes, we would have full NNLO accuracy.

Several methods have been proposed for NLO+PS merging, although the accuracy that they really achieve is still a debated matter [64, 380–384]. In particular, in the calculations of Refs. [380, 381], carried out in the frameworks of the Sherpa and Mc@Nlo collaborations respectively, merging is performed using a merging scale. One clusters the event using some jet clustering procedure, characterized by a merging scale $Q_0$, and uses the generator with the appropriate number of jets. In [381], stability under variations of the merging scale is interpreted as an indication of accuracy. In Ref. [383], NLO accuracy is adjusted by forcing the inclusive distribution to agree with the NLO one. This is achieved by subtracting appropriate terms, with a procedure dubbed UNLOPS (standing for “Unitary” NLOPS). In Ref. [64], within the so called GENEVA framework, the merging scale is defined in such a way that resummation can be carried out up to the NNLL level. In Ref. [385] a method (called MiNLO) was proposed to improve the accuracy of generators involving the production of associated jets, in such a way that it becomes reliable also after integrating out the associated jets.

In particular, in Ref. [386] it was shown that in certain simple cases the MiNLO method applied to generators for a boson (Higgs, *Z* or *W*) plus one jet, can be refined in such a way that observables that are inclusive in the associated jet (i.e. such that the associated jet is integrated ou) becomes NLO accurate.

In Ref. [387] a first NNLO+PS accurate generator for Higgs production in gluon fusion was presented, based upon the MiNLO procedure of Ref. [386]. The same method discussed above was also applied recently to the Drell–Yan process [63]. In Refs. [53, 388] NNLOPS generators were built for the Drell–Yan process and for Higgs production respectively.

In Ref. [389], a general strategy for NNLO+PS generators based upon the GENEVA framework was outlined. No complete application of this method to physical processes has been published, although preliminary results on the Drell–Yan process have been presented at conferences [390].

## Conclusions

At present generators for NLO calculations matched to parton shower are obtainable with a certain ease for processes with up to four particles in the final state. It is conceivable to imagine that automated generators for electroweak corrections for generic processes may become available soon. While generators for merged mutltijet samples (i.e. for processes with an arbitrary number of associated jets), with LO accuracy, have been available for quite some time, NLO-accurate merged generators are now beginning to appear. NNLO calculation for processes with up to two particles in the final state have recently appeared for a considerable number of processes, and NNLO calculation matched to shower generators have appeared only for Higgs production in gluon fusion and Drell–Yan processes. It is concievable that within the next decade NNLO calculations matched to shower will become generally available, and that the problem of merging for NLO generators will be solved.

## Tools for precision electroweak physics

In this section we give a brief overview of the state of the art of the tools for precision electroweak physics, in view of the forthcoming experiments at the LHC Run-II and the prospects of developments for future experiments at very high energy colliders, like the FCC-hh and FCC-ee. Some emphasis will be put on codes for hadronic collisions, while for ${{\mathrm{e}}{}{}}^{+} {{\mathrm{e}}{}{}}^{-}$ colliders we will refer to the state of the art at the end of the LEP data analysis, discussing some issues and prospects relevant for future high luminosity/energy machines.

### Hadron colliders

As already noted in Sect. 8, the experimental precision foreseen for LHC Run-II will require the inclusion of the complete SM, both the QCD and the electroweak part, in the evaluation of quantum corrections for accurate simulations. The processes that have been most accurately measured, where the inclusion of electroweak radiative corrections is already mandatory, are charged and neutral Drell–Yan, in addition to Higgs channels for the precise determination of its properties. In the past, i.e. at Tevatron and LHC Run-I, the simulations and analyses have been performed by exploiting the dominance of QED LL photonic emission from external leptons and the relative suppression of QED radiation from quarks with respect to gluon radiation. In practice this was achieved by describing final state leptonic QED radiation by means of process-independent codes such as PHOTOS [391] or internal algorithms provided by the shower MC itself, as for instance in HERWIG++ [392], PYTHIA(8) [343] and SHERPA [350, 393]. With the ultimate precision reached at Tevatron measurements, in particular the combined CDF and D0 ${\mathrm{W}}{}{}$-boson mass measurement [394], a more precise theoretical description of Drell–Yan processes became necessary, at least for the estimate of the systematic uncertainties induced by the approximate factorized QCD$\otimes$QED$+$PS approach of the simulation tools. In fact, several complete fully-differential electroweak NLO calculations are available in the literature and implemented in corresponding simulation codes, such as HORACE [49, 50], RADY [47, 395, 396], SANC [48, 397], WGRAD [45], WINHAC [398, 399], and ZGRAD [46]. These codes share the common feature of LO QCD and NLO electroweak accuracy. Several detailed comparisons exist in the literature [400–403], which allow to understand the level of technical as well as physical precision reached on the electroweak side of the calculations. Among the fixed order codes, it is worth mentioning that SANC can calculate the NLO contributions of ${\mathcal O}(\alpha _{\mathrm{s}})$ and ${\mathcal O}(\alpha )$, while the code FEWZ [404] adds up the EW NLO corrections to the QCD NNLO corrections for the neutral Drell–Yan process. The HORACE generator includes also the effect of all order photonic effects, consistently matched to the NLO calculation without double counting, in analogy with the QCD NLOPS codes such as MC@NLO and POWHEG. Only recently a consistent merging of NLO EW and NLO QCD corrections within a single event generator, matched with higher order QED and QCD emissions has been achieved within the POWHEG framework [74, 405]. An independent implementation has been presented in Ref. [73], where the higher order shower corrections are given by the QCD shower only. In this way also terms of ${\mathcal O}(\alpha \alpha _{\mathrm{s}})$ are included. In particular, terms of ${\mathcal O}(\alpha )$ dressed with soft/collinear QCD radiation and terms of ${\mathcal O}(\alpha _{\mathrm{s}})$ dressed with soft/collinear QED radiation are correctly accounted for. The remaining ${\mathcal O}(\alpha \alpha _{\mathrm{s}})$ terms are a source of theoretical uncertainty which can be assessed by comparison with a complete two-loop ${\mathcal O}(\alpha \alpha _{\mathrm{s}})$ calculation. At present such a calculation has been carried out in the pole approximation for the charged and neutral Drell–Yan processes [76]. A solid estimate of these and NNLO EW perturbative contributions will be crucial for future precision measurements of the ${\mathrm{W}}{}{}$-boson mass at the LHC (see Sect. 2). The complete NNLO calculation, beyond the pole approximation, will be a challenge for future theoretical advances.

Besides the Drell–Yan processes, exact NLO EW calculations exist for a limited number of final states, such as dijets, ${\mathrm{V}}{}{}+ 1$ jet, ${\mathrm{t}}{}{} \bar{{\mathrm{t}}{}{}}{}{}$, single-top, ${\mathrm{V}}{}{}({=}{\mathrm{W}}{}{},{\mathrm{Z}}{}{},{\upgamma }{}{}) + 3$ jets, ${\mathrm{H}}{}{}+ {\mathrm{V}}{}{}$, ${\mathrm{H}}{}{}+ 1$ jet, and ${\mathrm{H}}{}{}+ 2$ jets. The recent progress in the automation of NLO QCD calculations, described in Sect. 8 is being extended to include also the calculation of NLO EW corrections. There are in principle no obstacles to this, even if the EW corrections are more involved due to the presence of different mass scales circulating in the loops, together with the presence of unstable particles and chiral interactions. Several groups are working in this direction: GoSaM [345, 406], HELAC-NLO [407], MadLoop [107, 408], OpenLoops [347] and Recola [346]. First complete results obtained with automated tools appeared in Refs. [409–412]. During the 2013 edition of the Les Houches Workshop on Physics at TeV Colliders a “High Precision Wish List” has been proposed [413], which can be considered as the goal of the high precision calculations for the coming years. By inspection of Tables 1 through 3 of Ref. [413], we can see that the NLO EW corrections, consistently added to the (N)NLO QCD ones and matched with higher order QCD/QED PS contributions are required for all the processes in the tables. This list of processes will allow to fully exploit the LHC Run-II data in understanding the Standard Model. It is worth noticing that, in addition to the already discussed Drell–Yan processes, the consistent matching of NLO EW corrections with higher order QED PS is only available for Higgs decay to four leptons [414].

**Table 7:** Are we in the electroweak Sudakov zone yet? Taken from Ref. [426]

Process	$\sqrt{s}=8~\text {TeV}$	$\sqrt{s}=14~\text {TeV}$	$\sqrt{s}=33, 100~\text {TeV}$
Inclusive jet, dijet	Yes	Yes	Yes
Inclusive ${\mathrm{W}}{}{}/{\mathrm{Z}}{}{}$ tail	$\sim$Yes	Yes	Yes
${\mathrm{W}}{}{}{\upgamma }{}{}$, ${\mathrm{Z}}{}{}{\upgamma }{}{}$ tail ($\ell {\upnu } {\upgamma }{}{}, \ell \ell {\upgamma }{}{}$)	No	$\sim$Yes	Yes
${\mathrm{W}}{}{}/{\mathrm{Z}}{}{}$ + jets tail	$\sim$Yes	Yes	Yes
${\mathrm{W}}{}{}{\mathrm{W}}{}{}$ leptonic	Close	$\sim$Yes	Yes
${\mathrm{W}}{}{}{\mathrm{Z}}{}{}$, ${\mathrm{Z}}{}{}{\mathrm{Z}}{}{}$ leptonic	No	No	Yes
${\mathrm{W}}{}{}{\mathrm{W}}{}{}, {\mathrm{W}}{}{}{\mathrm{Z}}{}{}, {\mathrm{Z}}{}{}{\mathrm{Z}}{}{}$ semileptonic	$\sim$Yes	Yes	Yes

Usually the size of the “genuine” EW corrections (i.e. excluding the leading terms of electromagnetic origin) is moderate, at the few percent level. However, when the scales involved in the considered scattering process become large with respect to $M_{{\mathrm{W}}{}{}}$, the NLO EW corrections can be particularly enhanced, because of the presence of logarithmic terms of the form $\alpha \ln ^2(Q^2/M_{{\mathrm{W}}{}{}}^2)$ and $\alpha \ln (Q^2/M_{{\mathrm{W}}{}{}}^2)$, where $Q^2$ is a typical energy scale of the process. These terms are known as Sudakov logarithms and correspond to the soft and collinear singularities of QCD and QED, induced by the presence of massless particles. In the case of the EW corrections, however, the ${\mathrm{W}}{}{}$ boson mass acts as a physical cutoff so that the virtual corrections can be considered separately from the real contributions,^6^ giving rise to large negative corrections in the phase space regions where $Q^2 \gg M_{{\mathrm{W}}{}{}}^2$. Moreover, on pure theoretical grounds, the cancellation of Sudakov logarithms in the EW sector can only be partial, due to the incomplete summation of the contribution of *SU*(2) doublets in the initial state. The Sudakov logarithmic structure of the electroweak corrections has been studied in detail in the literature [415–421] and a general algorithm able to extract, in a process-independent way, the coefficients of the double and single logarithms has been presented in Refs. [422, 423]. Such an algorithm has been recently implemented in the ALPGEN event generator, with first phenomenological results for ${\mathrm{Z}}{}{}/{\upgamma }{}{} +$ jets production [424], a particularly important background for the search of new physics in the kinematic regime at the LHC. Further studies at the energies of 33 and $100~\text {TeV}$, typical reference energies for future hadronic colliders, have been carried out within the 2013 Snowmass Community Summer Study [425]. For example, for a few selected processes, such as dijet production, inclusive vector boson production, ${\mathrm{V}}{}{}$ + jets, and vector boson pair production, it has been shown that, in the extreme regions probed at the LHC with $\sqrt{s} = 8~\text {TeV}$, the electroweak effects on the tails of some distributions become of the same order of magnitude of the experimental accuracy. This means that with the future Run-II of the LHC we will enter the Sudakov zone, where the EW corrections are relevant for data analysis and will be even more important for higher energies, as shown in Table 7, where the size of the corrections can reach several tens of percent [426]. With such large effects also the issue of the resummation of EW corrections should be addressed, as suggested in Refs. [420, 427–430].

### Lepton colliders

The simulation tools for lepton colliders can be grouped in two different classes, according to the physics purpose: generators for the precise luminosity determination on the one side and programs for the analysis of the large angle data. These two kinds of theoretical tools allow for the completion of a high precision physics program of an ${{\mathrm{e}}{}{}}^{+} {{\mathrm{e}}{}{}}^{-}$ collider.

#### Event generators for luminosity

The luminosity can be determined through a counting measurement of a process which has a large cross section and is calculable to a high accuracy, such as the small angle Bhabha scattering. This process is in fact largely dominated by QED *t*-channel photon exchange and its cross section can be calculated perturbatively with a high level of accuracy. During the LEP1 and LEP2 eras the reference generator for small angle Bhabha scattering was BHLUMI [431, 432], which was based on QED NLO corrections to *t*-channel scattering, supplemented with higher-order corrections in the Yennie–Frautschi–Suura exponentiation approach. The physical precision of BHLUMI was scrutinized by means of independent calculations, such as for instance SABSPV [433], mainly based on QED NLO precision plus higher-orders photonic corrections in the QED structure function approach. The final theoretical accuracy on Bhabha scattering at LEP1 was at the level of $0.05\,\%$.

The experience gained at LEP has been fruitful for the development of Monte Carlo tools for the luminosity determination at the low-energy flavour factories by means of large angle Bhabha scattering, cross-checked with ${{\mathrm{e}}{}{}}^{+} {{\mathrm{e}}{}{}}^{-} \rightarrow {\upgamma }{}{} {\upgamma }{}{}$ measurements. In this context the first QED parton shower matched to the NLO fixed order calculation for the QED processes ${{\mathrm{e}}{}{}}^{+} {{\mathrm{e}}{}{}}^{-} \rightarrow {{\mathrm{e}}{}{}}^{+} {{\mathrm{e}}{}{}}^{-}$, ${{\mathrm{e}}{}{}}^{+} {{\mathrm{e}}{}{}}^{-} \rightarrow {{\upmu }{}{} ^{+}} {{\upmu }{}{} ^{-}}$ and ${{\mathrm{e}}{}{}}^{+} {{\mathrm{e}}{}{}}^{-} \rightarrow {\upgamma }{}{} {\upgamma }{}{}$, BABAYAGA@NLO, has been realized [434–436]. In parallel, an impressive effort has been devoted to the calculation of the exact NNLO QED corrections to Bhabha scattering, see for example Ref. [437] and references therein. A future consistent inclusion of these results into Monte Carlo generators could push the accuracy to the level of a few $0.01\,\%$, at least as far as QED corrections are concerned.

A source of theoretical uncertainty (driven by experimental uncertainties) is the hadronic contribution to the running of the QED coupling constant $\Delta \alpha _{\mathrm {had}}(q^2)$, which is derived from low energy data through dispersion relations. In this context, the present measurements at low energy machines are extremely important to reduce the dominant uncertainties at LEP.

It is worth mentioning that an alternative process to Bhabha scattering for luminometry is ${{\mathrm{e}}{}{}}^{+} {{\mathrm{e}}{}{}}^{-} \rightarrow {\upgamma }{}{} {\upgamma }{}{}$, which is not affected, at least up to NNLO order, by the error on $\Delta \alpha _{\mathrm {had}}$ and thus, in principle, it could be calculated with higher theoretical precision.

#### Simulation tools for ${\mathrm{Z}}{}{}$ and ${\mathrm{W}}{}{}$ bosons at FCC-ee

Given the available statistics at LEP1, a $0.1\,\%$ precision level was reached for most of the observables. With such a level of precision, the necessary ingredients for the simulation tools (event generators and seminalitical programs, such as for instance KORALZ [438, 439], TOPAZ0 [440–443] and ZFITTER [444–447]) were the exact NLO EW corrections to the ${{\mathrm{e}}{}{}}^{+} {{\mathrm{e}}{}{}}^{-} \rightarrow \hbox {f}{}{}\bar{\hbox {f}{}{}}{}{}$ hard scattering, convoluted with QED final and initial state radiation. Since around the ${\mathrm{Z}}{}{}$ resonance the latter contribution is very large, of the order of $30\,\%$, higher order effects were included through the Yennie–Frautschi–Suura formalism or the QED structure function approach. In order to match the target accuracy, also higher order effects of weak and QCD origin, contributing for instance to the $\rho$ and $\Delta r$ parameters, had to be included in the computational tools. It is clear that a future GigaZ run of an ${{\mathrm{e}}{}{}}^{+} {{\mathrm{e}}{}{}}^{-}$ collider will require complete EW NNLO calculations, supplemented with improved higher order QED corrections. While the theoretical framework of Standard Model two-loop renormalization has been set in Refs. [448–450], the calculation of observables at NNLO accuracy for the processes ${{\mathrm{e}}{}{}}^{+} {{\mathrm{e}}{}{}}^{-} \rightarrow \hbox {f}{}{}\bar{\hbox {f}{}{}}{}{}$ is still a challenge for the future.

The high luminosity run of a future ${{\mathrm{e}}{}{}}^{+} {{\mathrm{e}}{}{}}^{-}$ collider at energies close and above the ${\mathrm{W}}{}{}{\mathrm{W}}{}{}$, ${\mathrm{Z}}{}{}{\mathrm{Z}}{}{}$ and ${\mathrm{Z}}{}{}{\mathrm{H}}{}{}$ thresholds will be very challenging for the development of Monte Carlo codes able to provide precise theoretical predictions. In fact, at LEP2 most of the tree-level predictions for four-fermion final states were based on tree-level matrix element, supplemented with convolution with initial state radiation effects and leading electroweak corrections in the form of running couplings, together with a scheme for the treatment of the unstable virtual bosons (see Refs. [451–453] for a review). Complete NLO predictions for ${{\mathrm{e}}{}{}}^{+} {{\mathrm{e}}{}{}}^{-} \rightarrow 4$ fermions final states appeared only after the end of LEP2 operations [454–457]. Most probably the required theoretical accuracy at FCC-ee will be NNLO EW corrections to ${{\mathrm{e}}{}{}}^{+} {{\mathrm{e}}{}{}}^{-} \rightarrow 4$ fermion final states, interfaced with algorithms for the treatment of QED higher order initial state radiation, a very challenging task for the presently available theoretical knowledge.

## Conclusions

The SM is always the background to all of our experimental explorations. The discovery of the SM-like Higgs boson is a milestone in particle physics. Direct study of this boson will hopefully shed light on the mysteries surrounding the origin of the electroweak scale, and possibly provide insight into observations that remain unexplained by the SM.

In this report we have taken the viewpoint that in the next several years an important window to explore the theory space of physics beyond the Standard Model – perhaps the only window – will be provided by precision physics. This expectation is based on the twin observations that EFT provides the general framework for consistent calculation of higher orders in studying deviations from the standard model, and that ongoing and near future experiments can achieve an estimated per mille accuracy on precision Higgs and EW observables.

Effective field theory is superior to a generic parametrization of higher-dimensional operators (such as the so-called $\kappa$-framework of Ref. [17]) in that it automatically implements gauge symmetry and unitarity, and, as discussed in the introduction and then in Sect. 4, it may point to the ultraviolet completion which provides hints for the underlying theory. However, EFT itself is subject to assumptions and limitations that one should be aware. Firstly, in principle EFT is defined in a Wilsonian approach, in which heavy degrees of freedom are integrated above a cutoff. In practice, however, computations beyond leading order are performed in a continuum (cutoff-independent) EFT, in which heavy degrees of freedom are not integrated out, but rather compensated for through an appropriate matching calculation [458]. This implies that decoupling of heavy degrees of freedom is assumed. Furthermore, while being the only approach that can be systematically improvable by including higher dimension operators and higher-order corrections in QCD and EW, in practice the EFT will be compared to data at a given accuracy. For example, the impact of ${\mathrm {dim}} = 8$ operators in some key observables will need to be evaluated as well as possibly the effect of NLO EW corrections. Finally, the most common EFT parametrisations are based on a linear realisation of the gauge symmetry. Work on non-linear realisation can be found in Ref. [459].

This then raises the question of whether results from LHC should be cast in a language which is as much as possible independent of our current conceptual framework. Theoretical and phenomenological developments are currently making this increasingly possible at the level of data analysis and of comparison between data and theory. For instance, it is now increasingly clear that cross-sections should be published as differential as possible, at the fiducial level, without the subtraction of electroweak corrections, and so on. Old hadron collider data are often obsolete because, say, they were analyzed using outdated parton distributions and leading-order theory, or infrared-unsafe QCD definitions, and this should surely be avoided.

However, this it is not enough: LEP results, which were free of these problems, could be stored in the form of Pseudo-Observables (PO), see Refs. [460–462], thereby allowing experimentalists and theorists to meet half way, without theorists having to run full simulation and reconstruction and experimentalists not having to fully unfold to model-dependent parameter spaces. The situation at the LHC is harder not only because it is a hadron collider, with the corresponding aforementioned problem (so that at the LHC fiducial cross section’s should always be reported), but also because $4\hbox {f}{}{}$ decays are $40\,\%$ of $2\hbox {f}{}{}$ decays, so most of the time we face off-shell unstable particles, even at the ${\mathrm{H}}{}{}$ peak cross-section, and signal and background are then inextricably tangled and interfering.

It is thus important to build a simple platform to bridge between realistic observables and theory parameters working in the space of signals but having in mind the space of theories. Realistic proposals will necessarily involve a combination of fiducial observables, and pseudo-observables [463–465], linked through the language of effective Lagrangians [466–468].

## Footnotes

1 The $\beta$-version of the code that includes the gauge boson decay is available from the authors of the code.

2 The scope of the EFT approach assumes that no other new state of mass $m<\Lambda$ exists. While scenarios with light states are obviously interesting and worth investigating, a model-independent approach is not really suitable there. Interactions between SM particles could still be affected by loops where light new physics states could be propagating, leading to fully model-dependent dynamical features. Were this the case, a model dependent (possibly simplified) approach should be employed.

3 The calculation of the Higgs production cross section in the gluon fusion channel at N$^3$LO in QCD has recently been made available as a power series in the threshold expansion in Ref. [292].

4 After the completion of this manuscript, the missing ingredient for the computation of the three-loop massless multi-parton soft anomalous dimension matrix, a quadrupole contribution, was presented in [297].

5 For an in-depth and up-to-date discussion of these issues, see Ref. [191], and references therein.

6 The real corrections produce different final states, which usually in the experimental analysis are considered as different processes with respect to the one under consideration.
